# Synthesis of ergostane-type brassinosteroids with modifications in ring A

**DOI:** 10.3762/bjoc.13.229

**Published:** 2017-11-02

**Authors:** Vladimir N Zhabinskii, Darya A Osiyuk, Yuri V Ermolovich, Natalia M Chaschina, Tatsiana S Dalidovich, Miroslav Strnad, Vladimir A Khripach

**Affiliations:** 1 Institute of Bioorganic Chemistry, National Academy of Sciences of Belarus, Kuprevich st., 5/2, 220141 Minsk, Belarus; 2 Laboratory of Growth Regulators, Centre of the Region Haná for Biotechnological and Agricultural Research, Institute of Experimental Botany ASCR & Palacký University, Šlechtitelů 27, CZ-783 71 Olomouc, Czech Republic

**Keywords:** biosynthetic precursors, brassinosteroids, diols, epibrassinolide, epicastasterone, metabolites

## Abstract

Herein, we present a new strategy for the preparation of a broad range of brassinosteroid biosynthetic precursors/metabolites differing by the ring A fragment. The protocol is based on the use of readily available phytohormones of this class bearing a 2α,3α-diol moiety (epibrassinolide or epicastasterone) as starting materials. The required functionalities (Δ^2^-, 2α,3α- and 2β,3β-epoxy-, 2α,3β-, 2β,3α-, and 2β,3β-dihydroxy-, 3-keto-, 3α- and 3β-hydroxy-, 2α-hydroxy-3-keto-) were synthesized from 2α,3α-diols in a few simple steps (Corey–Winter reaction, epoxidation, oxidation, hydride reduction, etc.).

## Introduction

The group of steroid plant hormones called brassinosteroids (BS) currently comprises about 70 compounds [1]. It is generally accepted that only few of them (such as brassinolide, castasterone, epibrassinolide, etc.), possessing 2α,3α-, (22*R*,23*R*)-diol groups, B-lactone, and 6-ketone moieties, exhibit hormonal activity in plants, whereas other BS are considered to be either biosynthetic precursors or metabolites of the “real” phytohormones [2]. All these compounds are part of a multidimensional biosynthetic metabolic network, the functioning of which is still far from being completely understood. Evidently, the identification of as much as possible elements of this network would contribute to its better knowledge.

Because of the extremely low BS content in natural sources, studies on their identification almost always included a preliminary chemical synthesis of the compounds in question as standards for identification purposes. Typically, such syntheses were carried out by sequential introduction of functional groups into the starting molecule leading to more complex products [3]. This is a suitable approach for the preparation of one or a limited number of BS, but it rapidly becomes laborious if the synthesis of a large set of compounds is desired. Recently, we have proposed an alternative methodology to minor BS constituents that is based on the transformation of more complex compounds into simpler ones [4–7]. This strategy has now become even more attractive as some naturally occurring BS (e.g., epibrassinolide [8]) have found practical application in agriculture and thus are commercially available and a cheap source for further chemical modifications.

**Scheme 1:**
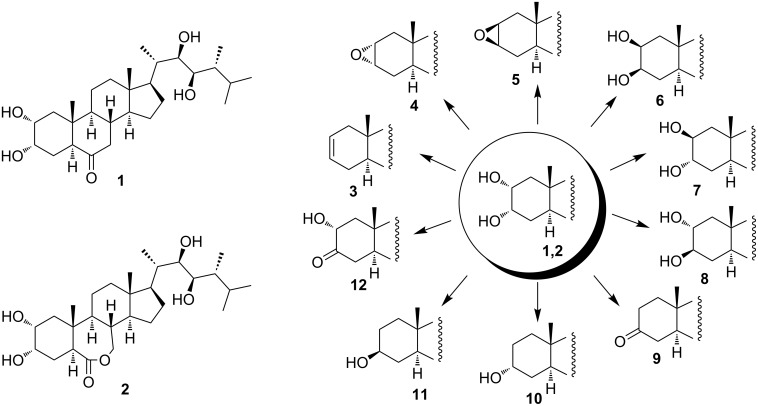
Structural features of epicastasterone (**1**), epibrassinolide (**2**) and A-ring units **3**–**12** of BS biosynthetic precursors/metabolites.

As a part of our programme aimed at the study of BS biosynthesis, we focussed in the present investigation on the preparation of a set of minor ergostane-type BS bearing A-ring structural units **3**–**12** (Scheme 1). The required functionalities were thought to be realized via a relatively short synthetic route starting from 2α,3α-diol fragment **13** of epicastasterone (**1**) or epibrassinolide (**2**).

## Results and Discussion

### Δ^2^-Steroids of type **3**

**Scheme 2:**
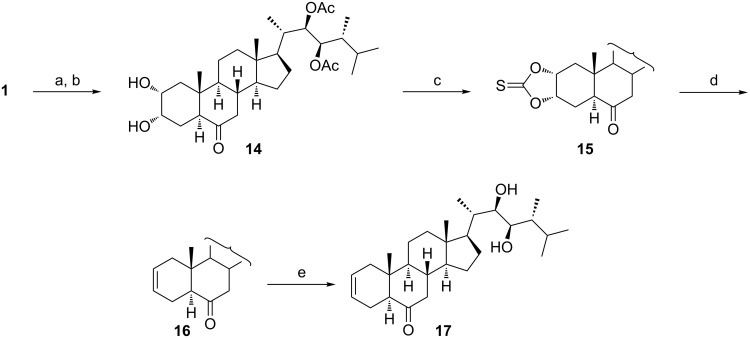
(a) Ac_2_O, Py, DMAP, 60 ^°^C; (b) K_2_CO_3_, MeOH, 20 °C (97% over 2 steps); (c) TCDI, DMAP, THF, 65 °C (76%); (d) (EtO)_3_P, 150 °C (94%); (e) KOH, MeOH, 65 °C (89%).

The possible existence of Δ^2^-steroids of type **3** (Scheme 1) as biosynthetic intermediates of BS was proposed in 1981 [9], but it lasted until 2003 when the corresponding compound named secasterol was found in seedlings of *Secale cereale* [10]. In subsequent years, a number of related Δ^2^-6-keto-22,23-diols was synthesized and assessed for biological activities in non-plant models. Some of the studied compounds showed a marked cytotoxicity against human cancer cell lines MCF-7 and LNCaP [11–13]. We tested two approaches for the transformation of epicastasterone (**1**) and epibrassinolide (**2**) into the corresponding Δ^2^-steroids. The first route comprised the selective protection of the side chain diol in **1** and **2** through exhaustive acetylation followed by saponification of the intermediate tetraacetates under controlled conditions [14]. Next, a Corey–Winter reaction [15] of the cyclic thiocarbonate **15** as a key reaction step gave the expected Δ^2^-olefin **16** in an excellent yield (Scheme 2). Subsequent deacetylation of **16** then afforded 24-episecasterol (**17**).

**Scheme 3:**
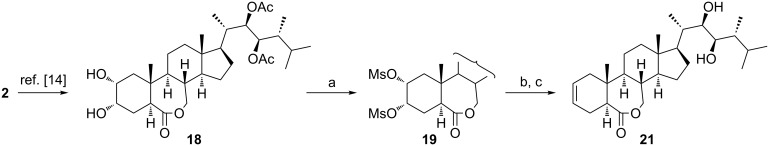
(a) MsCl, Py, 20 °C (95%); (b) Zn, NaI, DMF, 150 °C (83%); (c) KOH, MeOH, 65 °C (96%).

An alternative procedure to Δ^2^-steroids was applied for the preparation of the B-ring lactone derivative **21** (Scheme 3). The mesylation of diol **18** produced dimesylate **19**, which was treated with zinc dust and sodium iodide in refluxing DMF (Tipson–Cohen reaction [16]) to give, after deacetylation, B-lactone olefin **21** in 96% yield.

### 2α,3α- and 2β,3β-epoxides of type **4** and **5**

**Scheme 4:**
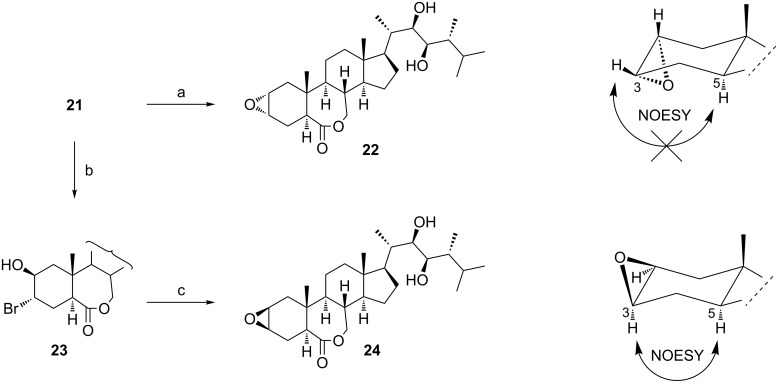
(a) MCPBA, CH_2_Cl_2_, 20 °C (90%); (b) NBS, DME, 20 °C; (c) KOH, MeOH, 20 °C (85% over 2 steps).

Olefins of type **3** are evident intermediates for the preparation of BS **4** and **5** with a 2,3-epoxide moiety (Scheme 1). To date, only the corresponding 6-ketones were found in natural sources (2,3-diepisecasterone [10] with an α-oriented epoxide **4** and two BS with a β-oriented epoxide **5**: secasterone [10,17] and 24-episecasterone [18]). The synthesis of both isomeric 2,3-epoxides **22** and **24** with a 7-membered B-ring lactone was accomplished starting from the olefin **21** (Scheme 4).

The reaction of peracids with Δ^2^-steroids possessing a six-membered B ring is known to proceed from the less hindered side of the molecule and results in the formation of 2α,3α-epoxides [19]. The 7-membered B ring in **21** had no influence on the stereochemical outcome of the reaction with MCPBA which resulted in the formation of the α-epoxide **22**. The same was true for the electrophilic addition of Br^+^ to the olefinic unit of **21**. It produced the trans-diaxial bromohydrine **23** that was transformed, on treatment with KOH, into the epoxide **24**. The structures of isomeric epoxides **22** and **24** were confirmed by their NOESY spectra as shown in Scheme 4. The obvious NOE correlation between H-3 and H-5 in **24** suggested the spatial vicinity of these two protons, thus the epoxide ring was β-oriented. On the contrary, no correlation was observed between the corresponding protons in compound **22**, which is consistent with the α-orientation of the epoxide ring.

### 2β,3β-, 2β,3α- and 2α,3β-diols of type **6**–**8**

**Scheme 5:**
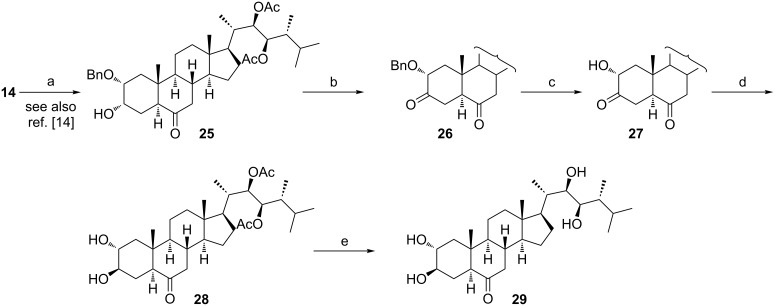
(a) BnBr, DMAP, Bu_2_SnO, TBAI, DIPEA, 110 °C (94%); (b) PCC, CH_2_Cl_2_, 20 °C (84%); (c) H_2_, Pd/C, 20 °C (99%); (d) NaBH_4_, EtOH, −25 °C (61%); (e) KOH, MeOH, 65 °C (96%).

Experiments on the identification of steroidal phytohormones in immature seeds of *Phaseolus vulgaris* revealed the existence of all possible configurations of vicinal hydroxy groups among the 2,3-stereoisomers of castasterone [20]. Minor BS constituents with structural fragments of types **6**–**8** (Scheme 1) showed reduced growth promoting biological activity when compared to castasterone, thus indicating that the epimerization of the hydroxy group at C-2/C-3 is the inactivation process contributing to a constant level of the active phytohormone. 2β,3β-Diols **6** are available through Woodward–Prévost *cis*-dihydroxylation of Δ^2^-steroids **3** [21]. The diaxial diols **7** can be easily synthesized by an acid-catalyzed opening of the 2α,3α-epoxides **4** [22–24] or 2β,3β-epoxides **5** [24]. The preparation of the diequatorial derivatives **8** requires more effort. The synthesis of 3,24-diepicastasterone (**29**), identified in immature seeds of *Phaseolus vulgaris* as a natural BS [25], started from the diol **14** (Scheme 5). The selective benzylation of its equatorial hydroxy group [14] followed by chlorochromate oxidation gave, after removal of the benzyl protecting group in **26**, the diketone **27**. Its reduction proceeded regio- and stereoselectively to afford the 2α,3β-diol **28**. Finally, treatment of this compound with KOH in MeOH led to the deprotected tetraol **29**.

### 3-Ketones of type **9**

**Scheme 6:**
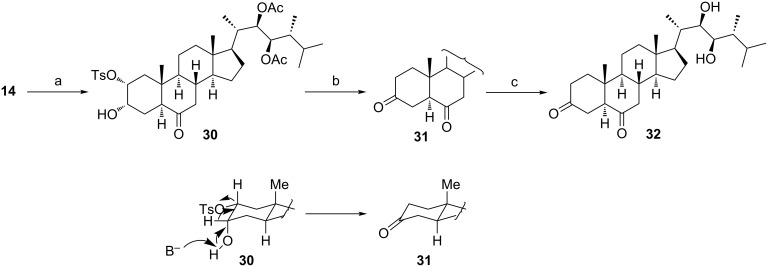
(a) TsCl, DMAP, Py, 30 °C (91%); (b) Py, 115 °C (65%); (c) KOH, MeOH, 20 °C (52%).

Three compounds of this type are known among natural BS: 3-dehydroteasterone [26–27], 3-dehydro-6-deoxoteasterone [28], and 3-dehydro-6-deoxo-28-norteasterone [29]. Our proposed approach to ketones of type **9** (Scheme 1) is based on a stereospecific C(3) → C(2)-hydride shift/elimination process [30]. So, tosylation of the diol **14** occurred regioselectively at the equatorial C-2 hydroxy group to give monotosylate **30**, which upon heating in pyridine, yielded compound **31** (Scheme 6). Its treatment with KOH in methanol led to 24-epi-3-dehydroteasterone **32**, which is unknown as a natural phytohormone.

### 3α- and 3β-alcohols of type **10** and **11**

**Scheme 7:**
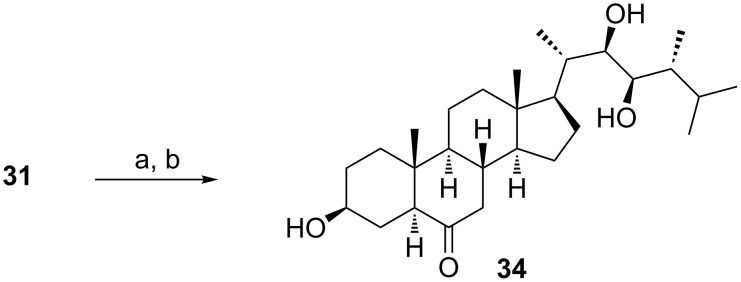
(a) NaBH_4_, EtOH, −25 °C (49%); (b) KOH, MeOH, 65 °C (85%).

The hydride reduction of 3,6-diketones proceeds in a regioselective manner at the C-3 position [19]. Both 3α- and 3β-alcohols of type **10** and **11** (Scheme 1) can be obtained in this way depending on the reducing agent used. Thus, the reaction of 3,6-diketones with K-selectride was shown to afford 3α-hydroxy-6-ketones [31]. On the other hand, treatment of the diketone **31** with NaBH_4_ followed by acetate deprotection led to 24-epiteasterone (**34**) having a 3β-hydroxy group on the A-ring (Scheme 7).

### 2α-Hydroxy-3-ketones of type **12**

**Scheme 8:**
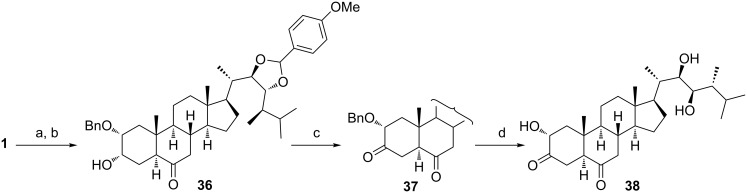
(a) Anisaldehyde, TMSCl, MeOH, 20 °C; (b) BnBr, DMAP, Bu_2_SnO, TBAI, DIPEA, 110 °C (86% over 2 steps); (c) PCC, CH_2_Cl_2_, 20 °C (81%); (d) H_2_, Pd/C, 20 °C (80%).

The only natural compound of type **12** (Scheme 1) known to date is 3-dehydro-24-epicastasterone (**38**) which was identified as a metabolite of 24-epicastasterone (**1**) in cell suspension cultures of *Lycopersicon esculentum* [32]. All attempts to prepare **38** through the intermediate **27** gave poor results, probably due to the low stability of the α-ketol moiety [24,33–34] under the conditions employed for the removal of the acetates at C-22 and C-23. The solution to this problem was the *p*-methoxybenzylidene protection of the 22,23-diol in epicastasterone (**1**). Its reaction with anisaldehyde proceeded regioselectively at the diol group of the side chain and gave, after benzylation at position 2, the alcohol **36** (Scheme 8). The oxidation of the latter product afforded compound **37**, which after deprotection, delivered the desired 3-dehydro-24-epicastasterone (**38**).

## Conclusion

In conclusion, we have developed a new strategy for synthesizing minor constituents of the class of BS phytohormones which is based on the use of readily available brassinosteroids of this class. Its advantage is that it allows preparing a full set of A-ring units (Δ^2^-, 2α,3α- and 2β,3β-epoxy-, 2α,3β-, 2β,3α-, and 2β,3β-dihydroxy-, 3-keto-, 3α- and 3β-hydroxy-, 2α-hydroxy-3-keto-) which are characteristic for biosynthetic precursors/metabolites of BS. The 2α,3α-diol function of ergostane BS was shown to be transformable into the required functionalities in only two to four standard chemical transformations.

## Supporting Information

File 1 General information, experimental details, characterization data and copies of ^1^H and ^13^C NMR spectra.
