# Supplementary material for: Synthesis of ergostane-type brassinosteroids with modifications in ring A
Source: Beilstein J Org Chem. 2017 Nov 2;13:2326–31. doi: 10.3762/bjoc.13.229 (PMC5687004; doi:10.3762/bjoc.13.229)
Supplement: File 1 — General information, experimental details, characterization data and copies of 1H and 13C NMR spectra. [file Beilstein_J_Org_Chem-13-2326-s001.pdf]

Supporting information

for

Synthesis of ergostane-type brassinosteroids with modifications in ring A

Vladimir N. Zhabinskii*[1], Darya A. Osiyuk[1], Yuri V. Ermolovich[1], Natalia M. Chaschina[1], Tatsiana S. Dalidovich[1], Miroslav Strnad[2], and Vladimir A. Khripach[1]

Address: [1]Institute of Bioorganic Chemistry, National Academy of Sciences of Belarus, Kuprevich st., 5/2, 220141 Minsk, Belarus and [2]Laboratory of Growth Regulators, Centre of the Region Haná for Biotechnological and Agricultural Research, Institute of Experimental Botany ASCR & Palacký University, Šlechtitelů 27, CZ-783 71 Olomouc, Czech Republic

Email: Vladimir N. Zhabinskii* - vz@iboch.bas-net.by

*Corresponding author

General information, experimental details, characterization data and copies of ^{1}H and ^{13}C NMR spectra

Contents

General remarks..S2

Procedures and spectroscopic analytical data S2

References..S14

NMR Spectra..S15

Experimental section

General remarks

All reactions that required anhydrous conditions were carried out under a positive argon flow with appropriately dried glassware, reagents and solvents. Commercially available reagents were used as received. Petroleum ether (PE) used had a boiling range of 60–90 °C. Reactions were monitored by TLC on silica gel GF_{254} plates (Merck). Column chromatography was performed using silica gel (200–300 mesh). One and two-dimensional nuclear magnetic resonance (NMR) spectra were obtained in $CDCl_3$ on a Bruker AVANCE 500 spectrometer. Chemical shift values are given in δ (ppm) relative to the residual solvent peaks: δ_H 7.26 and δ_C 77.0. Multiplicity was indicated as follows: s (singlet), d (doublet), t (triplet), q (quartet), m (multiplet), dd (doublet of doublet), ddd (doublet of doublet of doublets. High resolution mass spectra were recorded on a LTQ Orbitrap mass spectrometer coupled to an Accela HPLC System (HPLC column: Hypersyl GOLD, 50 mm × 1 mm, 1.9 µm).

Procedures and spectroscopic analytical data

(22*R*,23*R*,24*R*)-22,23-Diacetoxy-2α,3α-dihydroxy-24-methyl-5α-cholestan-6-one (14)

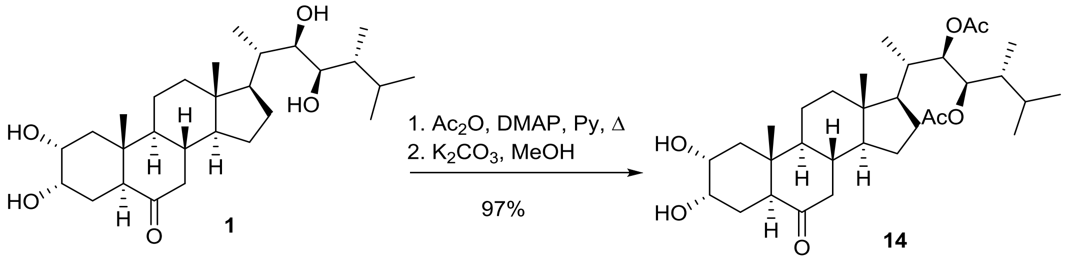

To a solution of epicastasterone (**1**, 406 mg, 0.874 mmol) in pyridine (2 mL), Ac_2O (495 µL, 5.24 mmol) and DMAP (8.5 mg, 0.070 mmol) were added. The mixture was kept at 60 °C for 16 h, then the solvent was evaporated in vacuo, the residue was dissolved in $CHCl_3$ (10 mL) and washed with saturated $NaHCO_3$ (3 × 3 mL). The organic layers were dried over Na_2SO_4 and evaporated. The crude tetraacetate (609 mg) was dissolved in MeOH (8.6 mL), and then a solution of K_2CO_3 (483 mg, 3.49 mmol) in water (3 mL) was added. The mixture was stirred at 20 °C for 1 h, diluted with water (10 mL), and extracted with $CHCl_3$ (3 × 20 mL). The combined organic extracts were dried over Na_2SO_4 and evaporated. The residue was chromatographed on SiO_2 ($CHCl_3$/MeOH 30:1→24:1) to give diacetate **14** (467 mg, 97%) as white crystals. Mp 104–107°C (hexane-EtOAc). ^{1}H NMR (500 MHz, $CDCl_3$) δ 5.22 (dd, J = 7.4, 0.8 Hz, 1H, 22-H), 5.05 (dd, J = 7.3, 4.9 Hz, 1H, 23-H), 4.03 (d, J = 2.2 Hz, 1H, 3H), 3.74 (ddd, J = 11.3, 4.3, 3.2 Hz, 1H, 2-H), 2.65 (dd, J = 12.5, 2.6 Hz, 1H, 5-H), 2.27 (dd, J = 13.1, 4.5 Hz, 1H), 2.04 (s, 3H, OAc), 2.02 (s, 3H, OAc), 0.98 (d, J = 6.7 Hz, 3H), 0.91 (d, J = 6.9 Hz, 3H), 0.84 (d, J = 6.8 Hz, 3H), 0.81 (d, J = 7.1 Hz, 3H), 0.73 (s, 3H, 19-H), 0.65 (s, 3H, 18-H). ^{13}C NMR (125 MHz, $CDCl_3$) δ 212.0, 170.6, 170.6, 74.6, 68.3, 68.2, 56.4, 53.6, 52.9, 50.7, 46.6, 42.8, 42.5, 40.1, 39.3, 38.7, 37.7, 37.6, 27.8, 26.8, 26.3, 23.8, 22.4, 21.1, 20.9, 20.9, 17.1, 13.5, 13.4, 11.7, 10.8. HRMS (ESI): calcd for $C_{32}H_{52}NaO_7^+$ $[M+Na]^+$ 571.3605, found 571.3608.

(22*R*,23*R*,24*R*)-2α,3α-*O*-Thionocarbonyl-22,23-diacetoxy-24-methyl-5α-cholestan-6-one (15)

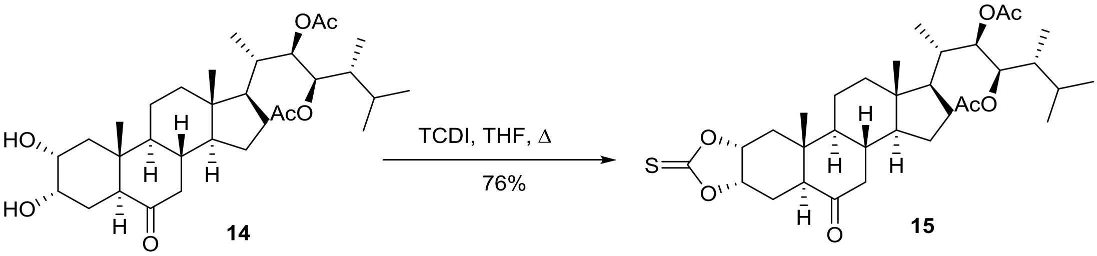

A mixture of diacetate **14** (959 mg, 1.75 mmol), 1,1'-thiocarbonyldiimidazole (934 mg, 5.24 mmol), DMAP (32 mg, 0.262 mmol), and dry THF (20 mL) was heated at 65 °C for 24 h. The solvent was evaporated and the residue was chromatographed on SiO_2 (PE/EtOAc 7:1→1:1) to give cyclic thionocarbonate **15** (783 mg, 76%) as white crystals. Mp 244-248°C (hexane-EtOAc). ^{1}H NMR (500 MHz, $CDCl_3$) δ 5.21 (d, *J* = 7.1 Hz, 1H, 22-H), 5.03 (dd, *J* = 6.9, 5.2 Hz, 1H, 23-H), 4.97 – 4.93 (m, 1H, 2- or 3-H), 4.89 (dt, *J* = 10.0, 6.7 Hz, 1H, 3- or 2-H), 2.51 (dd, *J* = 12.7, 3.7 Hz, 1H, 5-H), 2.03 (s, 3H, OAc), 2.01 (s, 3H, OAc), 0.97 (d, *J* = 6.7 Hz, 3H), 0.90 (d, *J* = 6.8 Hz, 3H), 0.83 (d, *J* = 6.7 Hz, 3H), 0.79 (d, *J* = 7.0 Hz, 3H), 0.68 (s, 3H, 19-H), 0.64 (s, 3H, 18-H). ^{13}C NMR (125 MHz, $CDCl_3$) δ 209.1, 191.6, 170.5, 79.8, 78.2, 74.4, 56.2, 52.8, 52.5, 50.5, 46.4, 42.6, 41.1, 38.9, 38.7, 38.5, 37.6, 37.3, 27.7, 26.8, 23.8, 22.4, 21.5, 21.0, 20.9, 20.8, 17.0, 13.3, 12.8, 11.5, 10.7. HRMS (ESI): calc for $C_{33}H_{50}NaO_7S^+$ [M+Na]+ 613.3169, found 613.3171.

(22*R*,23*R*,24*R*)-22,23-Diacetoxy-24-methyl-5α-cholest-2-en-6-one (16)

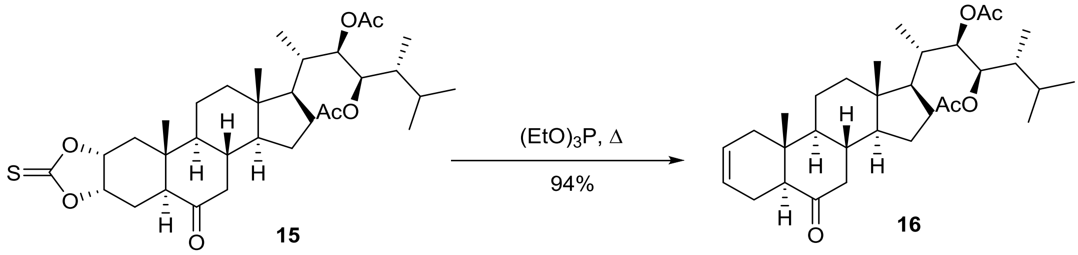

A mixture of thionocarbonate **15** (55 mg, 0.093 mmol) and $(EtO)_3P$ (1 mL) was heated at 150 °C for 6 h. After cooling to 20 °C, the mixture was transferred to a column filled with SiO_2. The column was eluted with a gradient of PE/EtOAc 8:1→5:1 to give olefin **16** (45 mg, 94%) as white crystals. Mp 185–189°C (hexane). ^{1}H NMR (500 MHz, $CDCl_3$) δ 5.66 (dd, *J* = 10.0, 2.4 Hz, 1H, 2-H), 5.54 (dd, *J* = 9.5, 1.5 Hz, 1H, 3-H), 5.23 (d, *J* = 7.3 Hz, 1H, 22-H), 5.05 (dd, *J* = 7.2, 4.9 Hz, 1H, 23-H), 2.03 (s, 3H, OAc), 2.01 (s, 3H, OAc), 0.99 (d, *J* = 6.7 Hz, 3H), 0.91 (d, *J* = 6.9 Hz, 3H), 0.84 (d, *J* = 6.7 Hz, 3H), 0.81 (d, *J* = 7.0 Hz, 3H), 0.69 (s, 3H, 19-H), 0.66 (s, 3H, 18-H). ^{13}C NMR (125 MHz, $CDCl_3$) δ 211.8, 170.52, 170.51, 124.9, 124.4, 74.6, 56.5, 53.8, 53.3, 52.9, 46.8, 42.7, 39.9, 39.4, 39.3, 38.7, 37.7, 37.6, 27.8, 26.8, 23.8, 22.4, 21.7, 21.0, 20.9, 20.8, 17.1, 13.5, 13.4, 11.6, 10.8. HRMS (ESI): calc for $C_{32}H_{50}NaO_5^+$ $[M+Na]^+$ 537.3550, found 537.3551.

(22*R*,23*R*,24*R*)-22,23-Dihydroxy-24-methyl-5α-cholest-2-en-6-one (17)

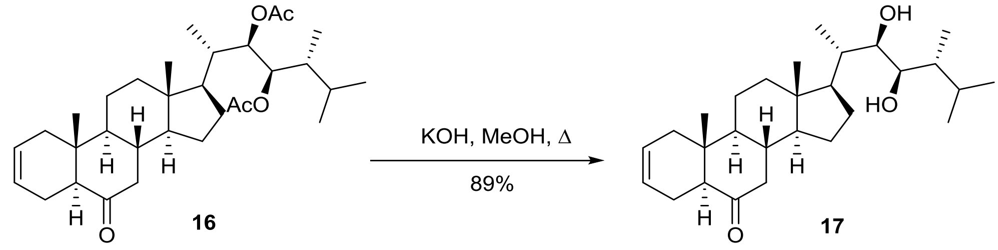

The diacetate **16** (44 mg, 0.085 mmol) was dissolved in a 5% KOH in MeOH (1 mL) and the solution was heated under heated at 65 °C for 1 h. The mixture was then cooled to 20 °C, acidified with 2 N HCl to pH 3 and extracted with $CHCl_3$ (3 × 5 mL). The combined organic extracts were dried over Na_2SO_4 and evaporated. The residue was chromatographed on SiO_2 (PE/EtOAc 5:1→1:1) to give diol **17** (33 mg, 89%) as white crystals. Mp 154–159°C (hexane-EtOAc). Lit. mp 166-168°C [1], 136-138°C [2]. ^{1}H NMR (500 MHz, $CDCl_3$) δ 5.67 (ddd, *J* = 9.7, 4.6, 1.9 Hz, 1H, 2-H), 5.56 (dd, *J* = 9.5, 1.9 Hz, 1H, 3-H), 3.69 (d, *J* = 3.5 Hz, 1H, 22-H), 3.40 (t, *J* = 5.2 Hz, 1H, 23-H), 0.97 (d, *J* = 6.6 Hz, 3H), 0.91 (d, *J* = 6.9 Hz, 3H), 0.86 (d, *J* = 6.8 Hz, 3H), 0.84 (d, *J* = 7.0 Hz, 3H), 0.70 (s, 3H, 19-H), 0.68 (s, 3H, 18-H). ^{13}C NMR (125 MHz, $CDCl_3$) δ 212.1, 124.9, 124.5, 76.3, 72.6, 56.6, 53.8, 53.3, 52.6, 46.9, 42.7, 41.4, 40.2, 40.0, 39.4, 39.3, 37.7, 27.7, 27.0, 23.9, 22.1, 21.7, 21.1, 17.3, 13.5, 12.4, 11.7, 10.8. HRMS (APCI): calc for $C_{28}H_{43}O^+$ $[M-2H_2O+H]^+$ 395.3308, found 395.3314.

(22*R*,23*R*,24*R*)-22,23-Diacetoxy-2α,3α-di(methylsulfonyloxy)-24-methyl-7a-homo-7-oxa-5α-cholestan-6-one (19)

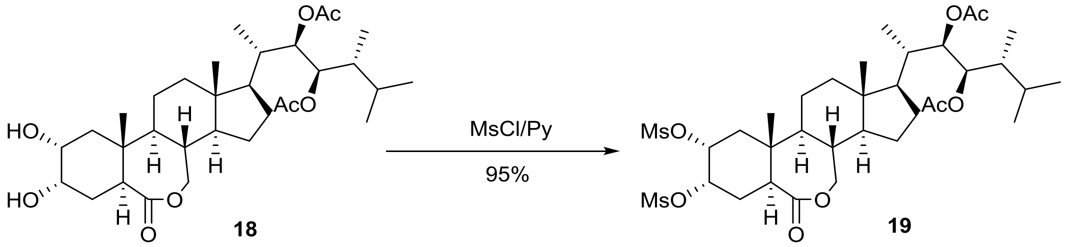

A mixture of diacetate **18** (302 mg, 0.535 mmol, prepared according to [3]), MsCl (0.206 mL, 2.68 mmol) and pyridine (1.5 mL) was kept at 20 °C overnight. The reaction mixture was diluted with water (3 mL) and extracted with $CHCl_3$ (3 × 3 mL). The combined extracts were dried over Na_2SO_4 and concentrated in vacuo. The residue was purified on SiO_2 ($CHCl_3$/MeOH 40:1→36:1) to give dimesylate **19** (365 mg, 95%) as a white foam. ^{1}H NMR (500 MHz, $CDCl_3$) δ 5.20 (d, *J* = 6.9 Hz, 1H, 22-H), 5.10 (br.s, 1H, 3-H), 5.04 (dd, *J* = 7.1, 5.0 Hz, 1H, 23-H), 4.73 (ddd, *J* = 12.7, 4.3, 2.4 Hz, 1H, 2-H), 4.10 (d, *J* = 12.0 Hz, 1H, 7-H), 4.04 (dd, *J* = 12.4, 9.3 Hz, 1H, 7-H), 3.09 (s, 3H, OMs), 3.08 (s, 3H, OMs), 2.03 (s, 3H, OAc), 2.01 (s, 3H, OAc), 0.96 (s, 3H, 19-H), 0.96 (d, *J* = 6.3 Hz, 3H), 0.91 (d, *J* = 6.9 Hz, 3H), 0.83 (d, *J* = 6.7 Hz, 3H), 0.80 (d, *J* = 7.0 Hz, 3H), 0.68 (s, 3H, 18-H). ^{13}C NMR (125 MHz, $CDCl_3$) δ 174.2, 170.5, 74.9, 74.4, 70.4, 57.6, 52.8, 51.0, 42.4, 41.2, 39.7, 39.2, 39.0, 38.8, 38.7, 38.7, 38.5, 37.6, 30.4, 29.6, 27.7, 26.8, 24.6, 22.4, 22.2, 20.9, 20.8, 17.0, 15.5, 13.3, 11.4, 10.7. HRMS (ESI): calc for $C_{34}H_{56}NaO_{12}S_2^+$ $[M+Na]^+$ 743.3105, found 743.3124.

(22*R*,23*R*,24*R*)-22,23-Diacetoxy-24-methyl-7a-homo-7-oxa-5α-cholest-2-en-6-one (20)

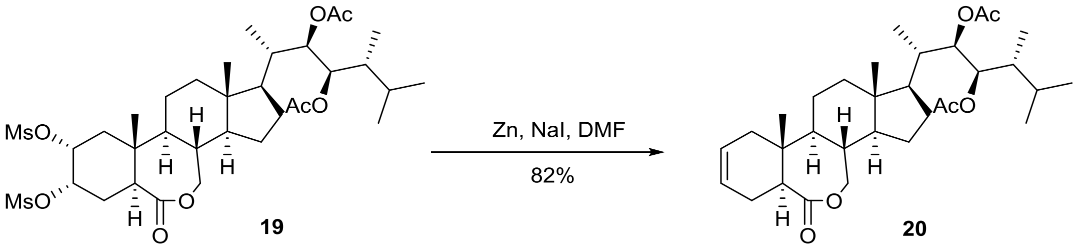

A mixture of dimesylate **19** (390 mg, 0.541 mmol), NaI (2.027 g, 13.5 mmol), Zn dust (884 mg, 13.5 mmol) and DMF (5.4 mL) was stirred at 150 °C for 2 h. After cooling to 20 °C, the precipitate was separated by filtration. The filtrate was diluted with water (50 mL) and extracted with EtOAc (2 × 40 mL). The combined organic layers were washed with saturated $Na_2S_2O_3$ (2 × 40 mL), dried over Na_2SO_4 and concentrated in vacuo. The residue was chromatographed on SiO_2 (PE/EtOAc 4:1→1:1) to give olefin **20** (235 mg, 82%) as white crystals. Mp 184–186 °C (PE-EtOAc). ^{1}H NMR (500 MHz, $CDCl_3$) δ 5.67 (m, 1H, 3-H), 5.56 (m, 1H, 2-H), 5.22 (m, *J* = 7.2 Hz, 1H, 22-H), 5.06 (dd, *J* = 7.0, 5.0 Hz, 1H, 23-H), 4.10 (d, *J* = 12.4 Hz, 1H, 7-H), 4.02 (dd, *J* = 12.0, 9.1 Hz, 1H, 7-H), 2.91 (dd, *J* = 10.2, 5.5 Hz, 1H, 5-H), 2.85 (m, 1H), 2.03 (s, 3H, OAc), 2.03 (s, 3H, OAc), 0.99 (d, *J* = 6.7 Hz, 3H), 0.93 (d, *J* = 6.8 Hz, 3H), 0.89 (s, 3H, 19-H), 0.86 (d, *J* = 6.7 Hz, 3H), 0.82 (d, *J* = 7.0 Hz, 3H), 0.70 (s, 3H, 18-H). ^{13}C NMR (125 MHz, $CDCl_3$) δ 176.4, 170.6 (x2), 123.6, 123.3, 74.6, 70.3, 59.1, 52.9, 51.4, 44.2, 42.5, 41.3, 39.8, 39.6, 38.7, 37.7, 35.7, 29.7, 27.8, 26.9, 25.9, 24.7, 22.5, 20.9, 20.9, 17.2, 16.4, 13.3, 11.4, 10.8. HRMS (ESI): calc for $C_{32}H_{50}NaO_6^+$ $[M+Na]^+$ 553.3500, found 553.3506.

(22*R*,23*R*,24*R*)-22,23-Dihydroxy-24-methyl-7a-homo-7-oxa-5α-cholest-2-en-6-one (21)

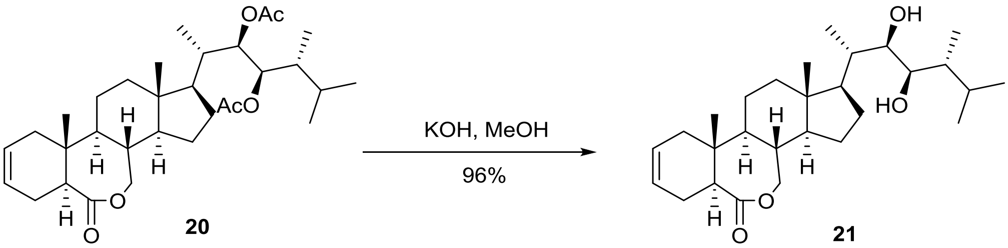

The title compound **21** (177 mg) was prepared as white crystals in 96% yield starting from diacetate **20** as described above for the preparation of **17**. Mp 177–179 °C (hexane-EtOAc). ^{1}H NMR (500 MHz, $CDCl_3$) δ 5.71 – 5.64 (m, 1H, 3-H), 5.59 – 5.52 (m, 1H, 2-H), 4.11 (d, *J* = 12.2 Hz, 1H, 7-H), 4.03 (dd, *J* = 12.3, 8.7 Hz, 1H, 7-H), 3.67 (d, *J* = 3.6 Hz, 1H, 22-H), 3.39 (t, *J* = 5.2 Hz, 1H, 23-H), 2.92 (dd, *J* = 10.3, 5.7 Hz, 1H, 5-H), 2.88 – 2.77 (m, 1H), 0.95 (d, *J* = 6.7 Hz, 3H), 0.91 (d, *J* = 6.9 Hz, 3H), 0.88 (s, 3H, 19-H), 0.86 (d, *J* = 6.8 Hz, 3H), 0.83 (d, *J* = 7.0 Hz, 3H), 0.70 (s, 3H, 18-H). ^{13}C NMR (125 MHz, $CDCl_3$) δ 176.5, 123.5, 123.4, 76.2, 72.5, 70.4, 59.0, 52.6, 51.3, 44.2, 42.4, 41.4, 41.2, 40.2, 39.8, 39.6, 35.7, 27.6, 27.0, 25.9, 24.7, 22.5, 22.1, 17.3, 16.4, 12.3, 11.6, 10.8. HRMS (ESI): calc for $C_{28}H_{46}NaO_4^+$ $[M+Na]^+$ 469.3288, found 469.3291.

(22*R*,23*R*,24*R*)-22,23-Dihydroxy-2α,3α-epoxy-24-methyl-7a-homo-7-oxa-5α-cholestan-6-one (22)

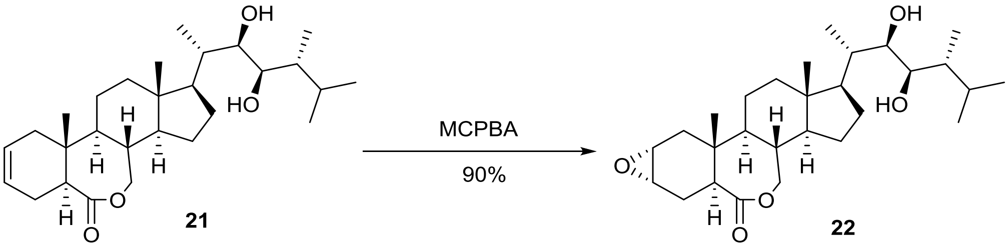

A solution of olefin **21** (56 mg, 0.125 mmol) and MCPBA (65 mg, 0.375 mmol) in CH_2Cl_2 (2 mL) was stirred at 20 °C for 1 h. The reaction mixture was washed with saturated $NaHCO_3$ (2 × 2 mL), brine (2 × 2 mL), dried over Na_2SO_4 and the solvent was evaporated under reduced pressure. The residue was purified on SiO_2 ($CHCl_3$/MeOH 100:1) to give epoxide **22** (52 mg, 90%) as white crystals. Mp 198-200 °C (hexane-EtOAc). ^{1}H and ^{13}C NMR data are given in Table 1. HRMS (ESI): calc for $C_{28}H_{47}O_5^+$ $[M+H]^+$ 463.3418, found 469.3412.

(22*R*,23*R*,24*R*)-22,23-Dihydroxy-2β,3β-epoxy-24-methyl-7a-homo-7-oxa-5α-cholestan-6-one (24)

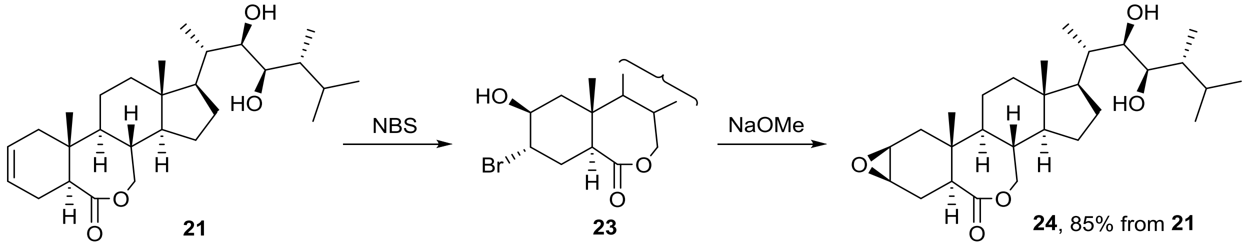

NBS (45 mg, 0.255 mmol) and water (1 mL) were added to a stirred solution of olefin **21** (57 mg, 0.128 mmol) in dimethoxyethane (6 mL). The reaction mixture was stirred at 20 °C for 1.5 h, then treated with saturated $Na_2S_2O_3$ (1 mL), diluted with water (3 mL) and extracted with EtOAc (3 × 5 mL). The combined organic extracts were dried over Na_2SO_4 and concentrated under reduced pressure. The crude bromohydrine **23** (75 mg) was dissolved in MeOH (12 mL) and treated with a 0.12 M MeONa in MeOH (2.4 mL, 0.281 mmol). The mixture was stirred at 20 °C for 15 min, then treated with saturated NH_4Cl (2 mL), diluted with water (2 mL) and extracted with EtOAc (2 × 10 mL). The combined organic layers were dried over Na_2SO_4 and concentrated under reduced pressure. The residue was chromatographed on SiO_2 ($CHCl_3$/MeOH 69:1→52:1) to give epoxide **24** (50 mg, 85%) as white crystals. Mp 200–203 °C (hexane-EtOAc). ^{1}H and ^{13}C NMR data are given in Table 1. HRMS (ESI): calc for $C_{28}H_{46}NaO_5^+$ $[M+Na]^+$ 485.3237, found 485.3234.

(22*R*,23*R*,24*R*)-22,23-Diacetoxy-3α-hydroxy-2α-benzyloxy-24-methyl-5α-cholestan-6-one (25)

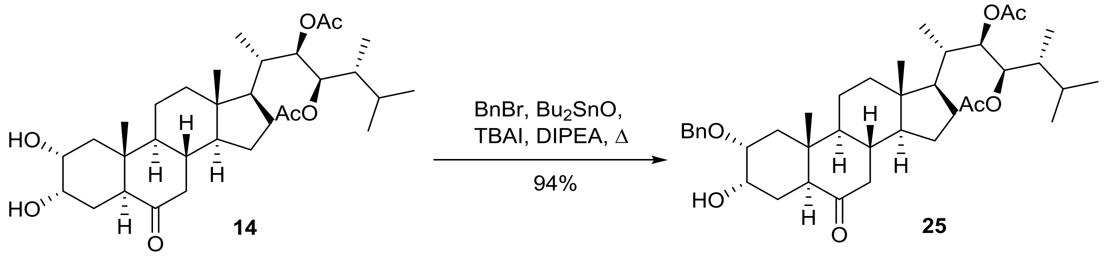

A mixture of diacetate **14** (148 mg, 0.270 mmol), Bu_2SnO (76.4 mg, 0.307 mmol), TBAI (38.5 mg, 0.104 mmol), DIPEA (118 μL, 0.675 mmol), BnBr (80.3 μL, 0.675 mmol) and dry toluene (3 mL) was stirred at 110 °C for 7 h. The reaction mixture was concentrated in vacuo and the residue was chromatographed on SiO_2 (PE/EtOAc 5:1→1:3) to give benzyl ether **25** (162 mg, 94%) as a yellow oil. Its ^1H and ^{13}C NMR spectra were identical to those described by us previously [3].

(22*R*,23*R*,24*R*)-22,23-Diacetoxy-2α-benzyloxy-24-methyl-5α-cholestan-3,6-dione (26)

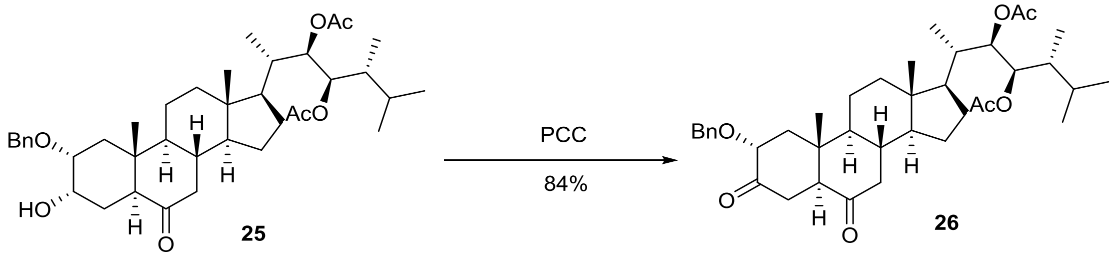

PCC (432 mg, 2.00 mmol) was added to a stirred solution of **25** (160 mg, 0.250 mmol) in CH_2Cl_2 (2 mL). The mixture was stirred at 20 °C overnight and the reaction mixture was transferred to a column with SiO_2. The column was eluted with PE/EtOAc 5:1→1:1 to give diketone **26** (134 mg, 84%) as white crystals. Mp 180–184 °C (hexane-EtOAc). ^1H NMR (500 MHz, $CDCl_3$) δ 7.39 – 7.27 (m, 5H, -OCH_2<u>Ph</u>), 5.22 (d, *J* = 7.2 Hz, 1H, 22-H), 5.05 (dd, *J* = 7.2, 5.0 Hz, 1H, 23-H), 4.88 (d, *J* = 11.6 Hz, 1H, -OC<u>H_2</u>Ph), 4.46 (d, *J* = 11.6 Hz, 1H, -OC<u>H_2</u>Ph), 4.04 (dd, *J* = 12.2, 6.5 Hz, 1H, 2-H), 2.03 (s, 3H, OAc), 2.02 (s, 3H, OAc), 0.99 (d, *J* = 6.7 Hz, 3H), 0.96 (s, 3H, 19-H), 0.92 (d, *J* = 6.9 Hz, 3H), 0.85 (d, *J* = 6.8 Hz, 3H), 0.81 (d, *J* = 7.1 Hz, 3H), 0.67 (s, 3H, 18-H). ^{13}C NMR (125 MHz, $CDCl_3$) δ 208.9, 208.2, 170.5 (x2), 137.7, 128.4 (x2), 127.8 (x3), 78.4, 77.4, 74.5, 72.4, 58.4, 56.2, 53.4, 52.9, 46.2 (x2), 42.8, 42.6, 39.1, 38.7, 37.7, 37.6, 36.6, 27.8, 26.8, 23.8, 22.4, 21.7, 20.9, 20.8, 17.1, 14.0, 13.4, 11.7, 10.7. HRMS (ESI): calc for $C_{39}H_{56}NaO_7^+$ $[M+Na]^+$ 659.3918, found 659.3926.

(22*R*,23*R*,24*R*)-22,23-Diacetoxy-2α-hydroxy-24-methyl-5α-cholestan-3,6-dione (27)

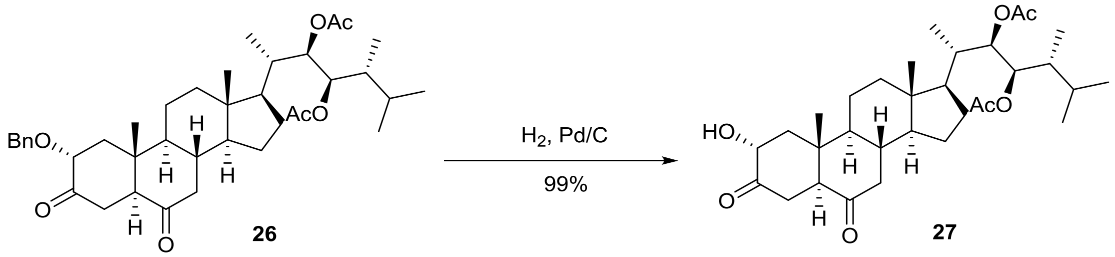

A solution of **26** (105 mg, 0.165 mmol) in THF (3.3 mL) was hydrogenated over Pd/C (10%, 17.5 mg) at 20 °C and atmospheric pressure. After 12 h, the mixture

was filtered through Celite, the solvent removed and the crude material was purified by chromatography on SiO_2 (PE/EtOAc 4:1→1:4) to give compound **27** (89 mg, 99%) as a colorless oil. ^{1}H NMR (500 MHz, $CDCl_3$) δ 5.21 (d, *J* = 7.1 Hz, 1H, 22-H), 5.04 (dd, *J* = 7.1, 5.1 Hz, 1H, 23-H), 4.23 (dd, *J* = 11.8, 7.1 Hz, 1H, 2-H), 2.02 (s, 3H, OAc), 2.01 (s, 3H, OAc), 1.02 (s, 3H, 19-H), 0.98 (d, *J* = 6.7 Hz, 3H), 0.91 (d, *J* = 6.9 Hz, 3H), 0.84 (d, *J* = 6.8 Hz, 3H), 0.80 (d, *J* = 7.0 Hz, 3H), 0.67 (s, 3H, 18-H). ^{13}C NMR (125 MHz, $CDCl_3$) δ 210.9, 207.9, 170.6 (x2), 74.5, 71.9, 58.5, 56.2, 53.3, 52.9, 47.7, 46.2, 42.8, 42.6, 39.1, 38.7, 37.7, 37.4, 35.0, 27.8, 26.8, 23.8, 22.4, 21.7, 20.9, 20.8, 17.1, 13.8, 13.4, 11.6, 10.7.

(22*R*,23*R*,24*R*)-22,23-Diacetoxy-2α,3β-dihydroxy-24-methyl-5α-cholestan-6-one (28)

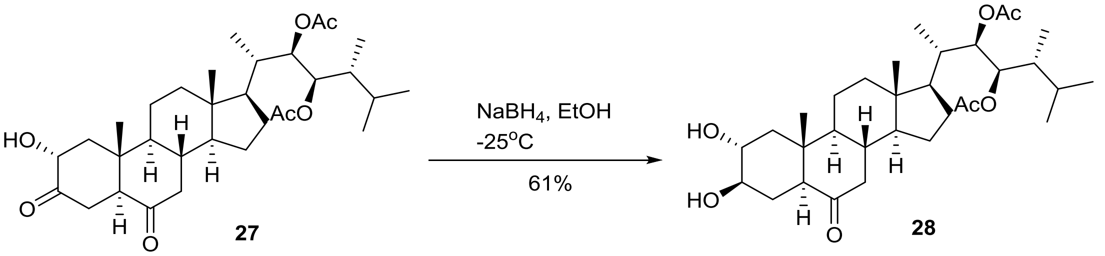

To a cooled (−25 °C) and stirred solution of diketone **27** (32 mg, 0.059 mmol) in absolute EtOH (19.5 mL), $NaBH_4$ (2.4 mg, 0.064 mmol) was added. Stirring was continued for 30 min at the same temperature, then water (10 mL) was added and the mixture was extracted with $CHCl_3$ (3 × 20 mL). The combined extracts were dried over Na_2SO_4 and concentrated in vacuo. The residue was chromatographed on SiO_2 ($CHCl_3$/MeOH 70:1→10:1) to give compound **28** (19.5 mg, 61%) as a white solid. ^{1}H NMR (500 MHz, $CDCl_3$) δ 5.23 (d, *J* = 7.1 Hz, 1H, 22-H), 5.06 (dd, *J* = 7.2, 5.0 Hz, 1H, 23-H), 3.66-3.54 (m, 1H, 2-H), 3.43-3.34 (m, 1H, 3-H), 2.04 (s, 3H, OAc), 2.02 (s, 3H, OAc), 0.99 (d, *J* = 6.7 Hz, 3H), 0.92 (d, *J* = 6.9 Hz, 4H), 0.85 (d, *J* = 6.8 Hz, 4H), 0.81 (d, *J* = 7.1 Hz, 4H), 0.79 (s, 3H, 19-H), 0.66 (s, 3H, 18-H). ^{13}C NMR (125 MHz, $CDCl_3$) δ 210.0, 170.6 (x2), 77.5, 75.7, 74.6, 72.0, 56.6, 56.4, 53.8, 52.9, 46.4, 44.2, 42.8, 39.3, 38.7, 37.7, 37.5, 29.7, 27.9, 27.8, 26.8, 23.8, 22.4, 21.6, 20.9, 20.9, 17.1, 14.3, 13.4, 11.7, 10.8.

(22*R*,23*R*,24*R*)-2α,3β,22,23-Tetrahydroxy-24-methyl-5α-cholestan-6-one (29)

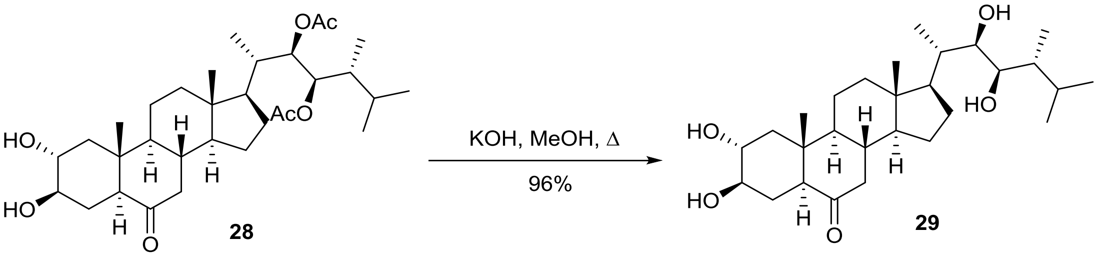

The title compound **29** (13 mg) was prepared as white crystals in 96% yield starting from diacetate **28** as described above for the preparation of **17**. Mp 210–213 °C (EtOAc). Lit. mp 209-212 °C [4], 213-215 °C [5]. ^{1}H NMR (500 MHz, C_5D_5N) δ 4.06 (dd, *J* = 14.2, 7.0 Hz, 2H, 3- and 22-H), 3.86 (ddd, *J* = 11.8, 8.9, 4.9 Hz, 1H, 2-H), 3.71 (dd, *J* = 5.9, 4.8 Hz, 1H, 23-H), 1.31 (d, *J* = 6.7 Hz, 3H), 1.07 (d, *J* = 6.8 Hz, 3H), 1.02 (d, *J* = 6.9 Hz, 3H), 0.98 (d, *J* = 6.9 Hz, 3H), 0.85 (s, 3H, 19-H), 0.70 (s, 3H, 18-H). ^{13}C NMR (125 MHz, C_5D_5N) δ 210.5, 76.7, 76.4, 72.8, 72.7, 57.3, 57.1, 54.3, 53.7, 47.1, 46.3, 43.4, 43.2, 42.6, 41.7, 40.3, 38.1, 29.8, 28.7, 27.6, 24.5, 22.9, 22.3, 17.9, 14.6, 13.7, 12.4, 11.8. HRMS (ESI): calc for $C_{28}H_{48}NaO_5^+$ $[M+H]^+$ 487.3394, found 487.3392.

(22*R*,23*R*,24*R*)-22,23-Diacetoxy-3α-hydroxy-2α-tosyloxy-24-methyl-5α-cholestan-6-one (30)

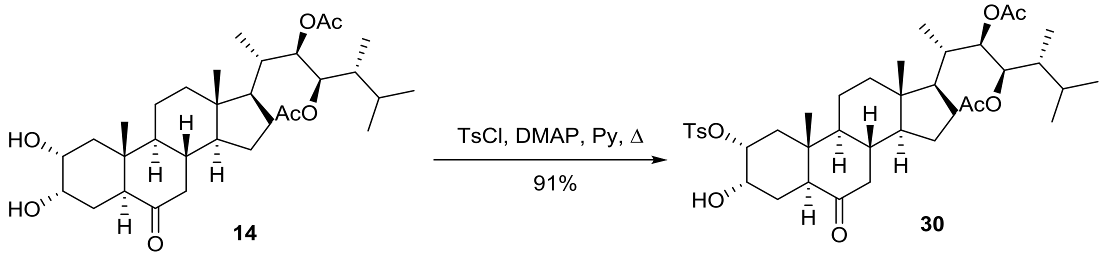

A mixture of diacetate **14** (206 mg, 0.375 mmol), pyridine (2 mL), TsCl (214 mg, 1.126 mmol) and DMAP (9 mg, 0.075 mmol) was kept overnight at 30 °C. Then water (10 mL) was added and the mixture was extracted with EtOAc (3 ×10 mL). The combined organic extracts were washed with water (2 × 10 mL), dried over Na_2SO_4 and evaporated. The residue was chromatographed on SiO_2 (PE/EtOAc 3:1→1:3) to give tosylate **30** (240 mg, 91%) as a colorless oil. ^{1}H NMR (500 MHz, $CDCl_3$) δ 7.78 (d, *J* = 8.2 Hz, 2H, OTs), 7.35 (d, *J* = 8.2 Hz, 2H, OTs), 5.22 (d, *J* = 7.2 Hz, 1H, 22-H), 5.05 (dd, *J* = 7.2, 5.0 Hz, 1H, 23-H), 4.54 (d, *J* = 11.7 Hz, 1H, 2-H), 4.07 (d, *J* = 2.2 Hz, 1H, 3-H), 2.65 (dd, *J* = 12.5, 2.8 Hz, 1H, 5-H), 2.45 (s, 3H, OTs), 2.25 (dd, *J* = 13.2, 4.5 Hz, 1H), 2.03 (s, 3H, OAc), 2.02 (s, 3H, OAc), 0.97 (d, *J* = 6.7 Hz, 3H), 0.91 (d, *J* = 6.9 Hz, 3H), 0.84 (d, *J* = 6.8 Hz, 3H), 0.80 (d, *J* = 7.0 Hz, 3H), 0.68 (s, 3H, 19-H), 0.63 (s, 3H, 18-H). ^{13}C NMR (125 MHz, $CDCl_3$) δ 210.8, 170.5, 145.1, 133.9, 129.9, 127.6, 80.7, 74.5, 66.7, 56.3, 53.4, 52.9, 50.1, 46.4, 42.7, 42.7, 39.2, 38.8, 37.7, 37.5, 37.3, 27.8, 26.8, 26.1, 23.8, 22.4, 21.6, 21.1, 20.9, 20.8, 17.1, 13.4, 13.3, 11.6, 10.8. HRMS (ESI): calc for $C_{39}H_{58}NaO_9S^+$ $[M+Na]^+$ 725.3694, found 725.3694.

(22*R*,23*R*,24*R*)-22,23-Diacetoxy-24-methyl-5α-cholestan-3,6-dione (31)

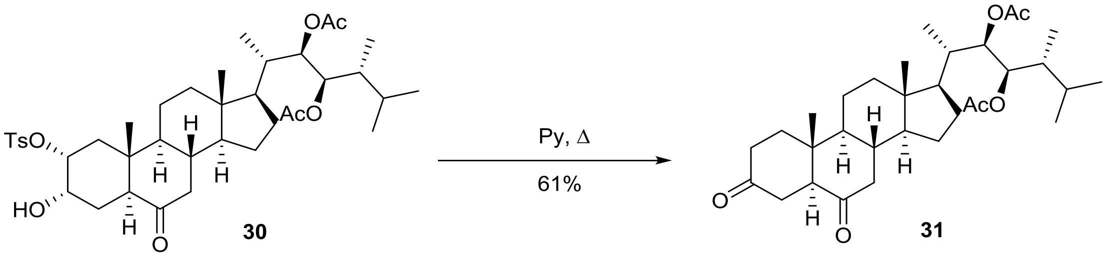

A solution of tosylate **30** (240 mg, 0.342 mmol) in pyridine (3 mL) was heated at 115 °C for 6 h. The solvent was removed in vacuo and the residue was purified by column chromatography on SiO_2 (PE/EtOAc 7:1→1:1) to give less polar diketone **31** (110 mg, 61%) as a colorless oil and starting tosylate **30** (17 mg, 7%). ^{1}H NMR (500 MHz, $CDCl_3$) δ 5.24 (dd, *J* = 7.3, 0.9 Hz, 1H, 22-H), 5.06 (dd, *J* = 7.3, 5.0 Hz, 1H, 23-H), 2.04 (s, 3H, OAc), 2.03 (s, 3H, OAc), 1.00 (d, *J* = 6.8 Hz, 3H), 0.95 (s, 3H, 19-H), 0.93 (d, *J* = 6.9 Hz, 3H), 0.85 (d, *J* = 6.8 Hz, 3H), 0.82 (d, *J* = 7.1 Hz, 3H), 0.69 (s, 3H, 18-H). ^{13}C NMR (125 MHz, $CDCl_3$) δ 211.1, 208.8, 170.5, 74.6, 57.5, 56.4, 53.4, 53.0, 46.5, 42.9, 41.2, 39.3, 38.8, 38.1, 38.0, 37.7, 37.3, 36.9, 27.9, 26.9, 23.9, 22.4, 21.6, 20.9, 20.8, 17.1, 13.4, 12.6, 11.7, 10.8. HRMS (ESI): calc for $C_{32}H_{50}NaO_6^+$ $[M+Na]^+$ 553.3500, found 553.3485.

(22*R*,23*R*,24*R*)-22,23-Dihydroxy-24-methyl-5α-cholestan-3,6-dione (32)

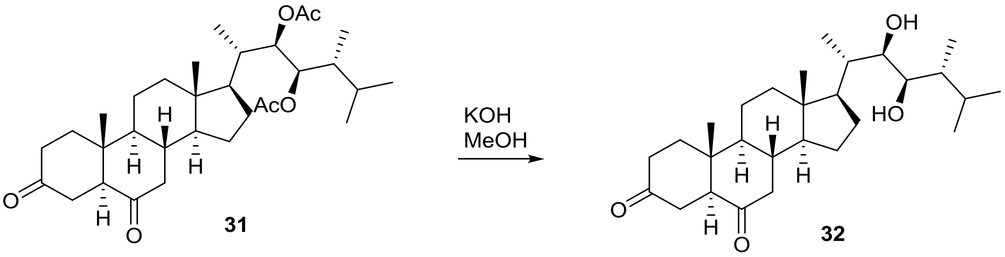

The diacetate **31** (32 mg 0.0603 mmol) was dissolved in a 5% KOH in MeOH (1 mL) and the solution was kept under argon at 20 °C for 5 h. The mixture was then acidified with 2 N HCl to pH 3 and extracted with $CHCl_3$ (3 × 3 mL). The combined organic extracts were dried over Na_2SO_4 and evaporated. The residue was chromatographed on SiO_2 (PE/EtOAc 3:1) to give diol **32** (14 mg, 52%) as an amorphous white powder. ^{1}H NMR (500 MHz, $CDCl_3$) δ 3.70 (dd, *J* = 4.4, 1.3 Hz, 1H, 22-H), 3.43 – 3.39 (m, 1H, 23-H), 0.98 (d, *J* = 6.7 Hz, 3H), 0.96 (s, 3H, 19-H), 0.92 (d, *J* = 6.9 Hz, 3H), 0.87 (d, *J* = 6.8 Hz, 3H), 0.85 (d, *J* = 7.0 Hz, 3H), 0.70 (s, 3H, 18-H). ^{13}C NMR (125 MHz, $CDCl_3$) δ 211.2, 209.0, 76.4, 72.6, 57.5, 56.4, 53.4, 52.6, 46.5, 42.9, 41.4, 41.2, 40.2, 39.3, 38.1, 37.3, 37.0, 27.7, 27.0, 23.9, 22.1, 21.7, 17.3, 12.6, 12.4, 11.9, 10.8. HRMS (ESI): calc for $C_{28}H_{46}NaO_4^+$ $[M+Na]^+$ 469.3288, found 469.3289.

(22*R*,23*R*,24*R*)-22,23-Diacetoxy-3β-hydroxy-24-methyl-5α-cholestan-6-one (33)

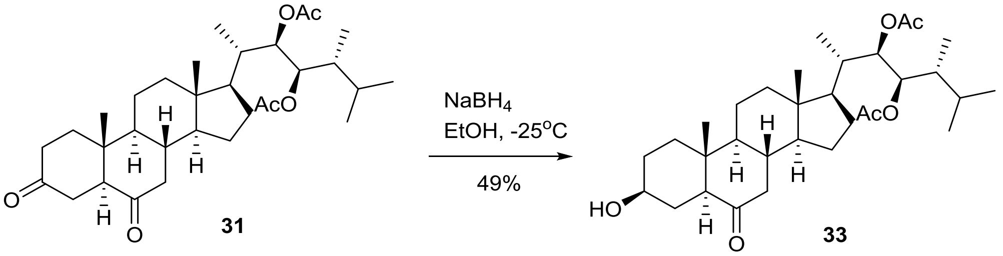

$NaBH_4$ (1 mg, 0.0456 mmol) was added to a cooled (−25 °C) solution of diketone **31** (22 mg, 0.0415 mmol) in absolute EtOH (13.5 mL) and the mixture was stirred at the same temperature for 30 min. Water (5 mL) was added and the mixture was extracted with $CHCl_3$ (3 × 10 mL). The combined extracts were dried over Na_2SO_4 and concentrated under reduced pressure. The residue was chromatographed on SiO_2 ($CHCl_3$/MeOH 100:1→94:1) to give alcohol **33** (9 mg, 49%) as white crystals. Mp 221 – 224°C ($CHCl_3$-MeOH). ^{1}H NMR (500 MHz, $CDCl_3$) δ 5.24 (dd, *J* = 7.4, 1.1 Hz, 1H, 22-H), 5.06 (dd, *J* = 7.3, 4.9 Hz, 1H, 23-H), 3.61 – 3.53 (m, 1H, 3-H), 2.04 (s, 3H, OAc), 2.03 (s, 3H, OAc), 0.99 (d, *J* = 6.8 Hz, 3H), 0.92 (d, *J* = 6.9 Hz, 3H), 0.85 (d, *J* = 6.8 Hz, 3H), 0.82 (d, *J* = 7.1 Hz, 3H), 0.75 (s, 3H, 19-H), 0.67 (s, 3H, 18-H). ^{13}C NMR (125 MHz, $CDCl_3$) δ 210.6, 2x170.6, 77.5, 74.6, 70.6, 56.7, 56.6, 53.8, 53.0, 46.6, 42.9, 40.9, 39.4, 38.8, 37.9, 37.7, 36.7, 30.7, 30.0, 27.9, 26.9, 23.9, 22.5, 21.5, 20.9, 20.9, 17.2, 13.4, 13.1, 11.7, 10.8. HRMS (ESI): calc for $C_{32}H_{52}NaO_6^+$ $[M+Na]^+$ 555.3656. Найдено 555.3636.

(22*R*,23*R*,24*R*)-3β,22,23-Trihydroxy-24-methyl-5α-cholestan-6-one (34)

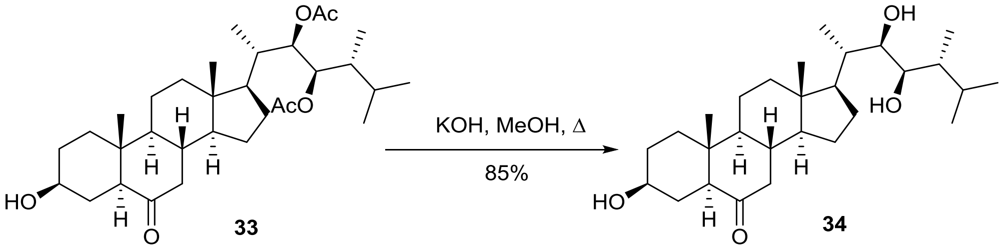

The title compound **34** (10 mg) was prepared as white crystals in 85% yield starting from diacetate **33** as described above for the preparation of **17**. Mp 233–236 °C ($CHCl_3$:MeOH). Lit. mp 233-234 °C [6]. ^{1}H NMR (500 MHz, C_5D_5N) δ 4.06 (br.s, 1H, 22-H), 3.85 (m, 1H, 3-H), 3.72 (br.s, 1H, 23-H), 1.33 (d, *J* = 5.9 Hz, 5H), 1.07 (d, *J* = 6.2 Hz, 3H), 1.03 (d, *J* = 6.4 Hz, 4H), 0.98 (d, *J* = 6.7 Hz, 3H), 0.79 (s, 3H, 19-H), 0.73 (s, 3H, 18-H). ^{13}C NMR (125 MHz, Pyr) δ 210.7, 76.7, 72.8, 70.4, 57.3, 57.2, 54.2, 53.8, 47.2, 43.4, 42.6, 41.7, 41.3, 40.4, 38.4, 37.4, 32.2, 31.6, 28.7, 27.7, 24.6, 22.9, 22.2, 17.9, 13.7, 13.6, 12.4, 11.8. HRMS (APCI): calc for $C_{28}H_{45}O_2^+$ $[M-2H_2O+H]^+$ 413.3413, found 413.3417.

(22*R*,23*R*,24*R*)-2α-Benzyloxy-3α-hydroxy-22,23-(*p*-methoxybenzylidenedioxy)-24-methyl-5α-cholestan-6-one (36)

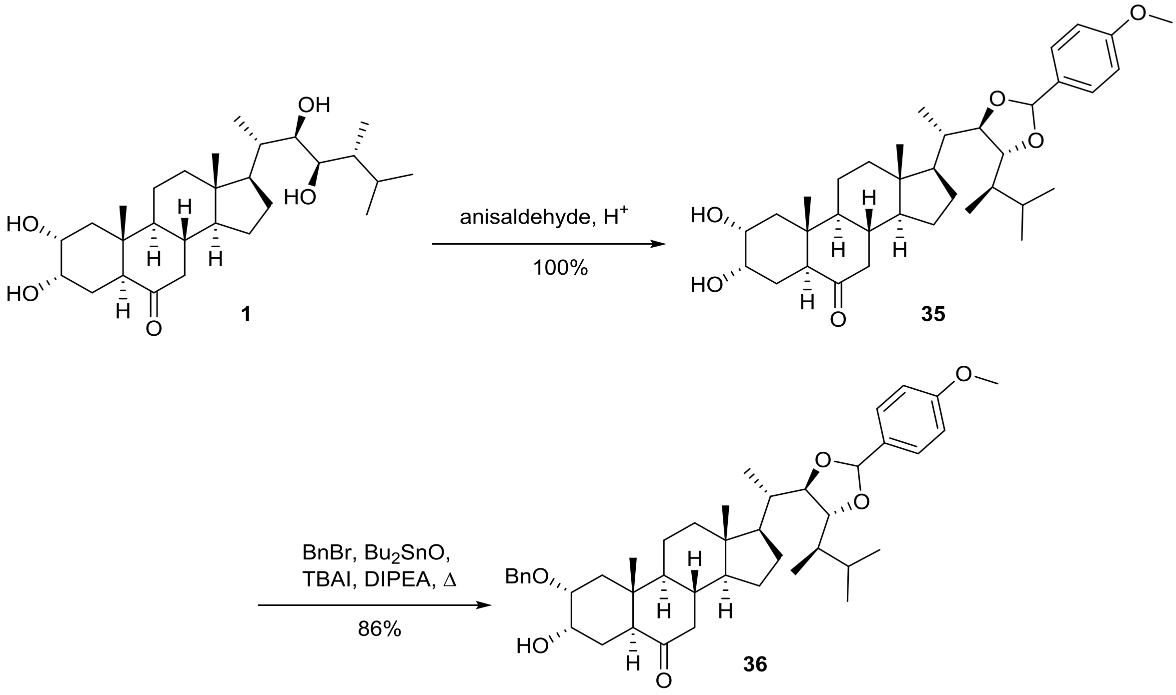

TMSCl (28 μL, 0.221 mmol) was added to MeOH (17 mL) and the solution was stirred at 20 °C for 10 min. Then, epicastasterone **1** (205 mg, 0.441 mmol) and anisaldehyde (161 μL, 1.323 mmol) were added and the mixture was stirred for further 40 min. The mixture was then treated with saturated $NaHCO_3$ (10 mL) and extracted with $CHCl_3$ (3 × 20 mL). The combined extracts were dried over Na_2SO_4 and concentrated in vacuo to give 257 mg of crude **35**. Its benzylation was performed according to the procedure described above for the preparation of **25**. The benzyl ether **36** (256 mg, 86%) was isolated as a yellow oil. ^{1}H NMR (500 MHz, $CDCl_3$) δ 7.44 (d, *J* = 8.6 Hz, 2H, arom.), 7.41 – 7.27 (m, 5H, arom.), 6.89 (dd, *J* = 8.4, 6.8 Hz, 2H, arom.), 4.59 (d, *J* = 11.7 Hz, 1H, -OC$\underline{H}_2$Ph), 4.54 (d, *J* = 11.7 Hz, 1H, -OC$\underline{H}_2$Ph), 4.18 (br.s, 1H, 22-H), 4.01 (m, 1H, 23-H), 3.799 and 3.796 (s, 3H, OMe), 3.70 (dd, *J* = 10.2, 5.3 Hz, 1H, 2-H), 3.53 – 3.46 (m, 1H, 3-H), 2.72 (d, *J* = 12.4 Hz, 1H), 1.17 – 0.62 (m, 18H). ^{13}C NMR (125 MHz, $CDCl_3$)

δ 212.1, 160.5, 138.0, 129.6, 128.5, 128.0, 127.8, 127.5, 113.7, 113.6, 104.1, 101.9, 83.4, 82.6, 80.9, 80.8, 75.6, 70.4, 65.4, 56.3, 55.3, 53.6, 53.1, 50.8, 46.6, 42.8, 42.4, 41.6, 40.0, 39.2, 39.0, 37.7, 37.4, 27.8, 27.7, 27.4, 25.4, 23.8, 21.2, 21.0, 20.9, 16.2, 15.7, 13.6, 13.2, 12.9, 11.7, 10.1, 8.8. HRMS (ESI): calc for $C_{43}H_{61}O_6^+$ $[M+H]^+$ 673.4463, found 673.4459.

(22*R*,23*R*,24*R*)-2α-Benzyloxy-22,23-(*p*-methoxybenzylidenedioxy)-24-methyl-5α-cholestan-3,6-dione (37)

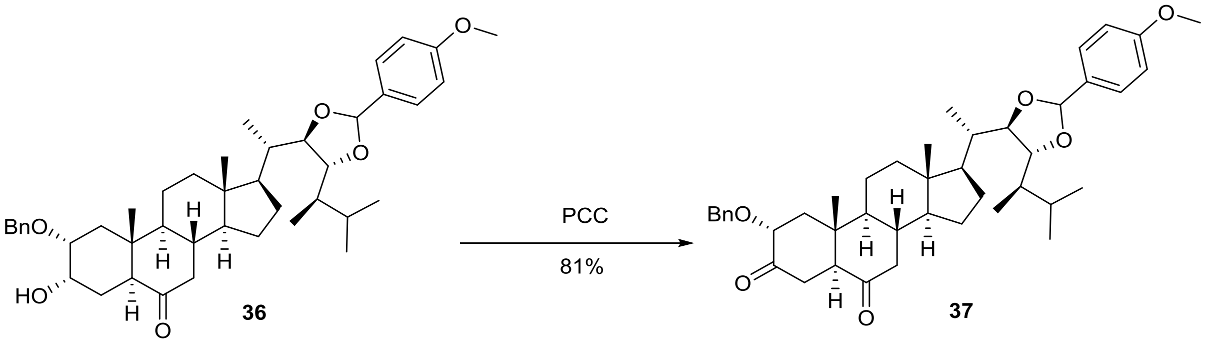

A mixture of **36** (256 mg, 0.381 mmol), PCC (657 mg, 3.05 mmol) and CH_2Cl_2 (3 mL) was stirred at 20 °C overnight. The reaction mixture was filtered through a plug of SiO_2 (EtOAc) and concentrated in vacuo. The residue was purified by column chromatography on SiO_2 (PE/EtOAc 7:1→1:1) to give ketone **37** (208 mg, 81%) as white crystals. Mp 184–188 °C (hexane-EtOAc). ^{1}H NMR (500 MHz, $CDCl_3$) δ 7.47 – 7.28 (m, 7H, arom.), 6.92 – 6.86 (m, 2H, arom.), 5.85 and 5.69 (s, 1H, -C<u>H</u>-C_6H_4OMe), 4.89 (d, *J* = 11.6 Hz, 1H, -C<u>H</u>$_2$Ph), 4.47 (d, *J* = 11.6 Hz, 1H, -C<u>H</u>$_2$Ph), 4.10 – 3.97 (m, 2H, 22- and 23-H), 3.796 and 3.792 (s, 3H, OMe), 3.70 (dd, *J* = 10.3, 5.3 Hz, 1H, 2-H), 1.12 (dd, *J* = 12.6, 6.5 Hz, 3H), 0.97 (s, 3H, 19-H), 0.95 – 0.71 (m, 9H), 0.70 (s, 3H, 18-H). ^{13}C NMR (125 MHz, $CDCl_3$) δ 208.8, 208.3, 160.5, 160.2, 137.7, 131.1, 129.6, 128.5, 128.4 (x2), 128.0, 127.8 (x4), 113.70, 113.65, 104.2, 101.9, 83.3, 82.6, 80.9, 80.8, 78.4, 77.3, 77.0, 76.7, 72.3, 58.3, 56.1, 55.3, 53.3, 53.1, 46.3, 46.2, 42.9, 42.6, 41.6, 40.0, 39.0, 38.9, 37.7, 36.6, 27.8, 27.6, 27.4, 23.9, 21.8, 21.0, 20.9, 16.2, 15.7, 13.9, 13.2, 12.9, 11.7, 10.1, 8.9. HRMS (ESI): calc for $C_{43}H_{58}NaO_6^+$ $[M+Na]^+$ 693.4126, found 693.4132.

(22*R*,23*R*,24*R*)-2α,22,23-Trihydroxy-24-methyl-5α-cholestan-3,6-dione / 3-dehydro-24-epicastasterone/ (38)

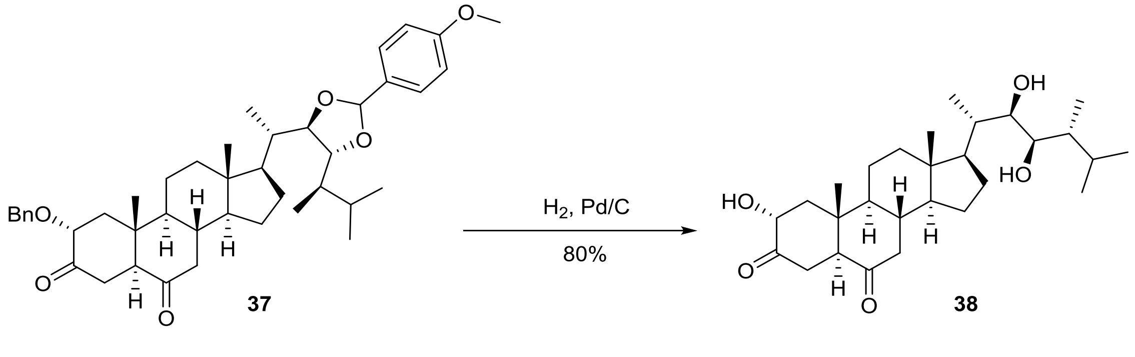

The title compound **38** (115 mg) was prepared as white crystals in 80% yield starting from **37** as described above for the preparation of **27**. Mp 190–193 °C (EtOAc). ^{1}H NMR (500 MHz, $CDCl_3$) δ 4.24 (dd, *J* = 11.9, 7.0 Hz, 1H, 2-H), 3.67 (br.s, 1H, 22-H), 3.39 (br.s, 1H, 23-H), 1.03 (s, 3H, 19-H), 0.96 (d, *J* = 6.3 Hz, 3H), 0.90 (d, *J* = 6.8 Hz, 3H), 0.85 (d, *J* = 6.7 Hz, 3H), 0.83 (d, *J* = 6.9 Hz, 3H),

0.69 (s, 3H, 18-H). ^{13}C NMR (125 MHz, $CDCl_3$) δ 211.0, 208.1, 76.3, 72.5, 72.0, 58.5, 56.3, 53.3, 52.5, 47.7, 46.3, 42.8, 42.7, 41.4, 40.2, 39.2, 37.5, 35.0, 27.7, 27.0, 23.9, 22.1, 21.8, 17.2, 13.8, 12.4, 11.8, 10.8. HRMS (ESI): calc for $C_{28}H_{43}O_3^+$ $[M-2H_2O+H]^+$ 427.3207, found 427.3210.

Table 1 – ^1H and ^{13}C NMR spectroscopic data for epoxides **22** and **24**[a,b,c]

Position	Compound			
	epoxide **22**		epoxide **24**	
	δ, H	δ, C	δ, H	□, C
1	1.62 m; 2.02	39.8	1.97 m; 2.77 m	24.5
2	3.29 m	52.0	3.21 m	52.1
3	3.13 dd (4.1, 4.1)	50.4	3.22 m	49.7
4	2.12 ddd (16.0, 4.5, 2.6); 2.51 ddd (16.0, 11.6, 1.0)	25.2	1.60 m; 2.38 d (15.6)	38.7
5	3.08 dd (11.5, 4.7)	41.2	2.72 m	42.8
6	-	176.0	-	175.5
7	3.99 dd (12.3, 8.8); 4.05 dd (12.3, 1.2)	70.1	3.98 dd (12.5, 9.0); 4.08 (12.5)	70.3
8	1.44	40.0	1.65 m	39.3
9	1.15	59.4	1.11 m	59.9
10	-	33.8	-	36.5
11	1.44 m; 1.66 m	22.6[d]	1.41 m; 1.72 m	22.3[d]
12	1.22 m; 1.97 m	39.6	1.20 m; 1.98 m	39.5
13	-	42.5	-	42.3
14	1.14 m	51.3	1.12	51.2
15	1.65 m	24.6[d]	1.21 m; 1.67 m	24.7[d]
16	1.31 m; 1.97 m	27.6[d]	1.31 m; 1.98 m	27.5[d]
17	1.53 m	52.5	1.53 m	52.5
18	0.68 s	11.7	0.68 s	11.5
19	0.89 s	17.9	0.90 s	22.1
20	1.44 m	40.2	1.45 m	40.2
21	0.94 d (6.7)	12.3	0.94 d (6.6)	12.3
22	3.65 br.s	72.4	3.66 d (3.4)	72.5
23	3.37 br.s	76.2	3.38 dd (5.2, 5.2)	76.2
24	1.47 m	41.4	1.48 m	41.4
25	1.88 m	26.9	1.88 m	26.9
26	0.84 d (6.8)[e]	17.2[e]	0.85 d (6.8)[e]	17.2
27	0.89 d (6.7)[e]	22.1[e]	0.89 d (6.6)[e]	17.6
28	0.82 d (7.0)	10.8	0.82 d (7.0)	10.8

[a] NMR chemical shifts (δ) are from spectra obtained in $CDCl_3$ solutions.
[b] Assigned by DEPT, COSY, HSQC, and HMBC experiments.
[c] *J* values (in Hz) in parentheses.
[d,e] May be reversed.

References

1. Khripach, V. A.; Zhabinskii, V. N.; Gulyakevich, O. V.; Konstantinova, O. V.; Misharin, A. Y.; Mekhtiev, A. R.; Timofeev, V. P.; Tkachev, Y. V. *Russ. J. Bioorg. Chem.* **2010**, *36*, 746-754. doi: 10.1134/S1068162010060117
2. Voigt, B.; Takatsuto, S.; Yokota, T.; Adam, G. *J. Chem. Soc., Perkin Trans. 1* **1995**, 1495-1498. doi: 10.1039/P19950001495
3. Zhylitskaya, H. A.; Litvinovskaya, R. P.; Zhabinskii, V. N.; Khripach, V. A. *Steroids* **2017**, *117*, 2-10. doi: 10.1016/j.steroids.2016.06.006
4. Voigt, B.; Porzel, A.; Adam, G.; Golsch, D.; Adam, W.; Wagner, C.; Merzweiler, K. *Collect. Czech. Chem. Commun.* **2002**, *67*, 91-102. doi: 10.1135/cccc20020091
5. Levinson, E. E.; Traven, V. F. *J Chem Res (S)* **1996**, 196-197
6. Voigt, B.; Schmidt, J.; Adam, G. *Tetrahedron* **1996**, *52*, 1997-2004. doi: 10.1016/0040-4020(95)01042-4

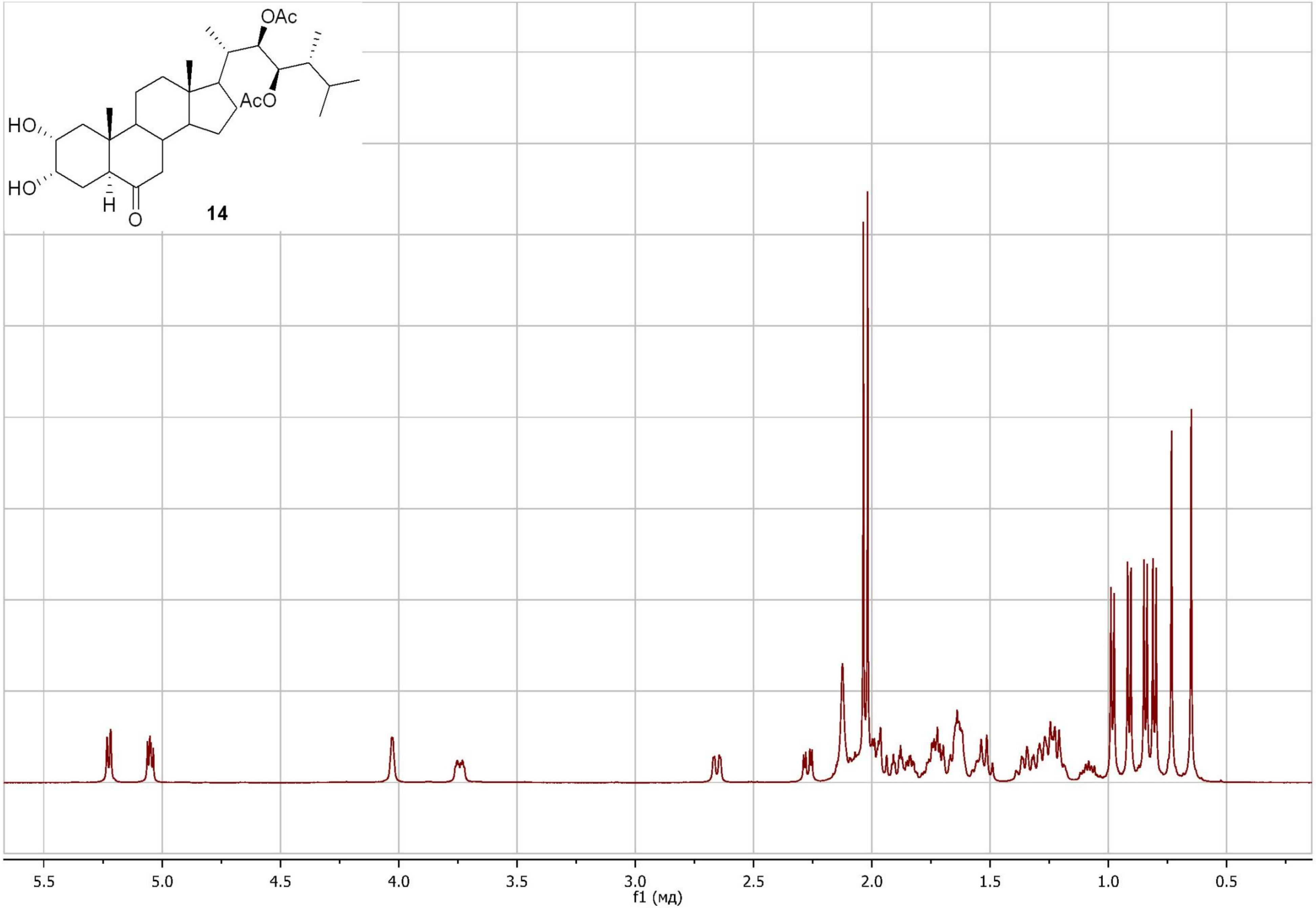
OAc
AcO
HO
HO
H
O
14
5.5
5.0
4.5
4.0
3.5
3.0
2.5
2.0
1.5
1.0
0.5
f1 (мд)

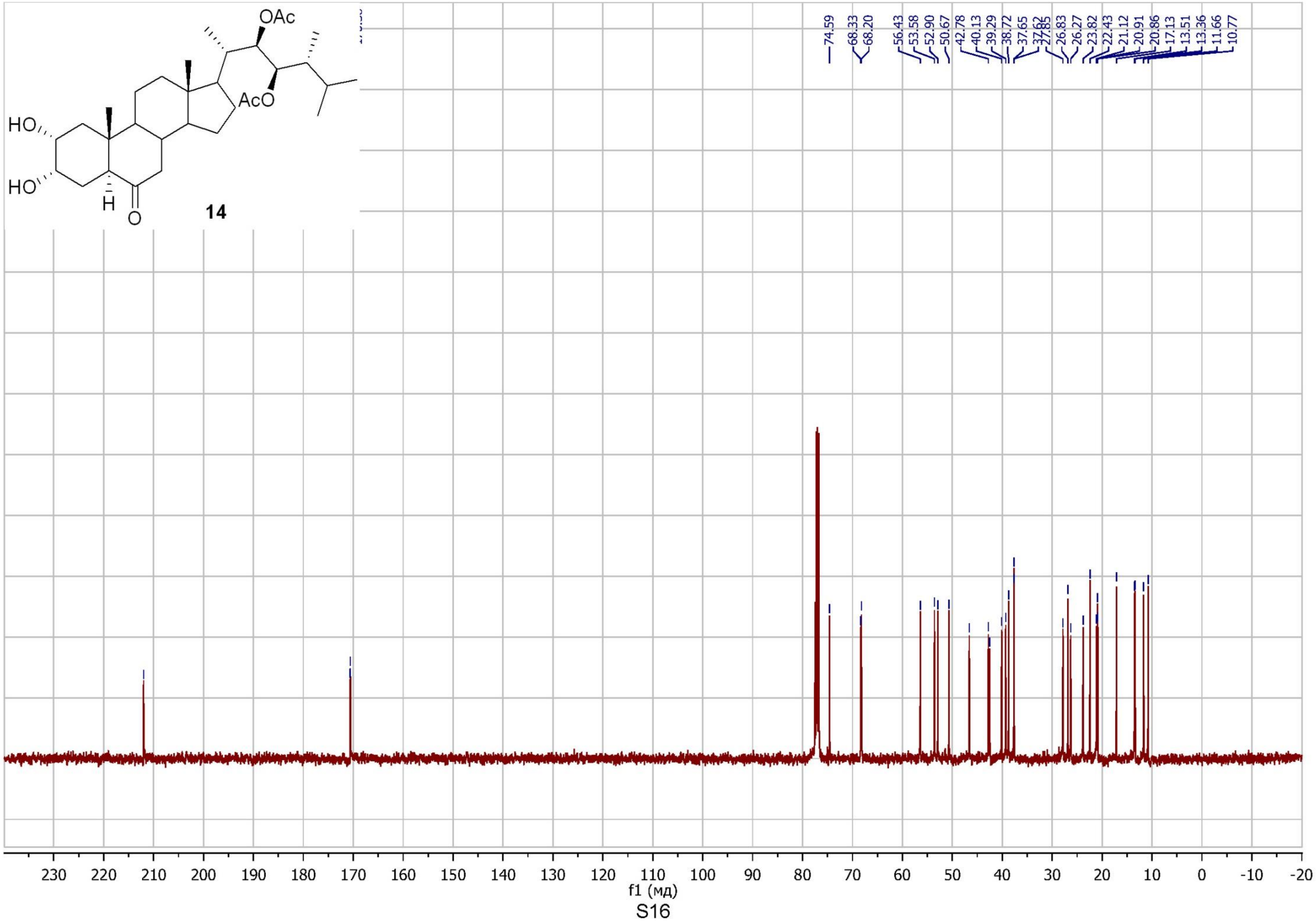

OAc
AcO
HO
HO
H
O
14
74.59
68.33
68.20
56.43
53.58
52.90
50.67
42.78
40.13
39.29
38.72
37.65
37.62
27.85
26.83
26.27
23.82
22.43
21.12
20.91
20.86
17.13
13.51
13.36
11.66
10.77
230 220 210 200 190 180 170 160 150 140 130 120 110 100 90 80 70 60 50 40 30 20 10 0 -10 -20
f1 (мд)

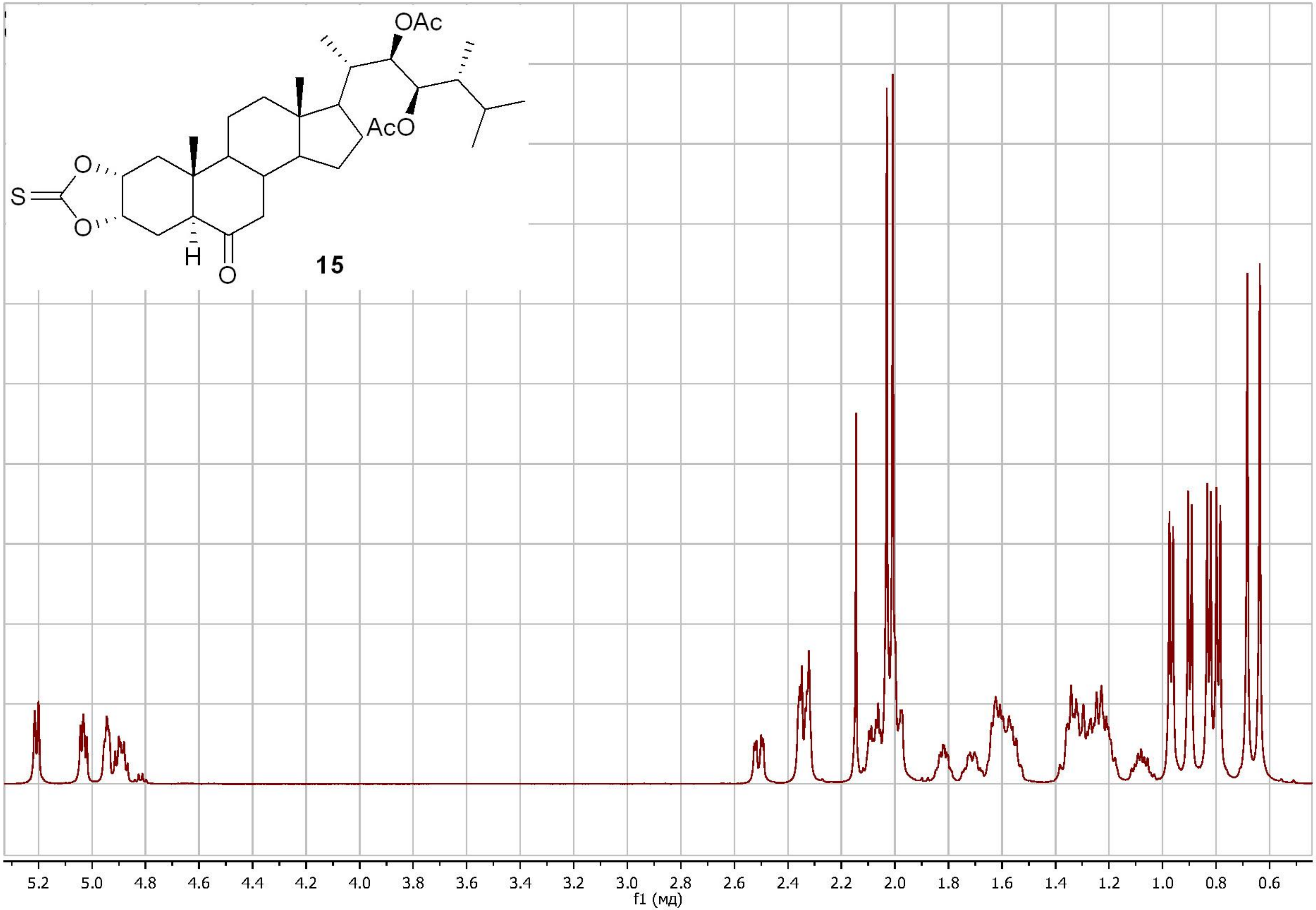
OAc
AcO
S
O
O
H
O
15
5.2 5.0 4.8 4.6 4.4 4.2 4.0 3.8 3.6 3.4 3.2 3.0 2.8 2.6 2.4 2.2 2.0 1.8 1.6 1.4 1.2 1.0 0.8 0.6
f1 (мд)

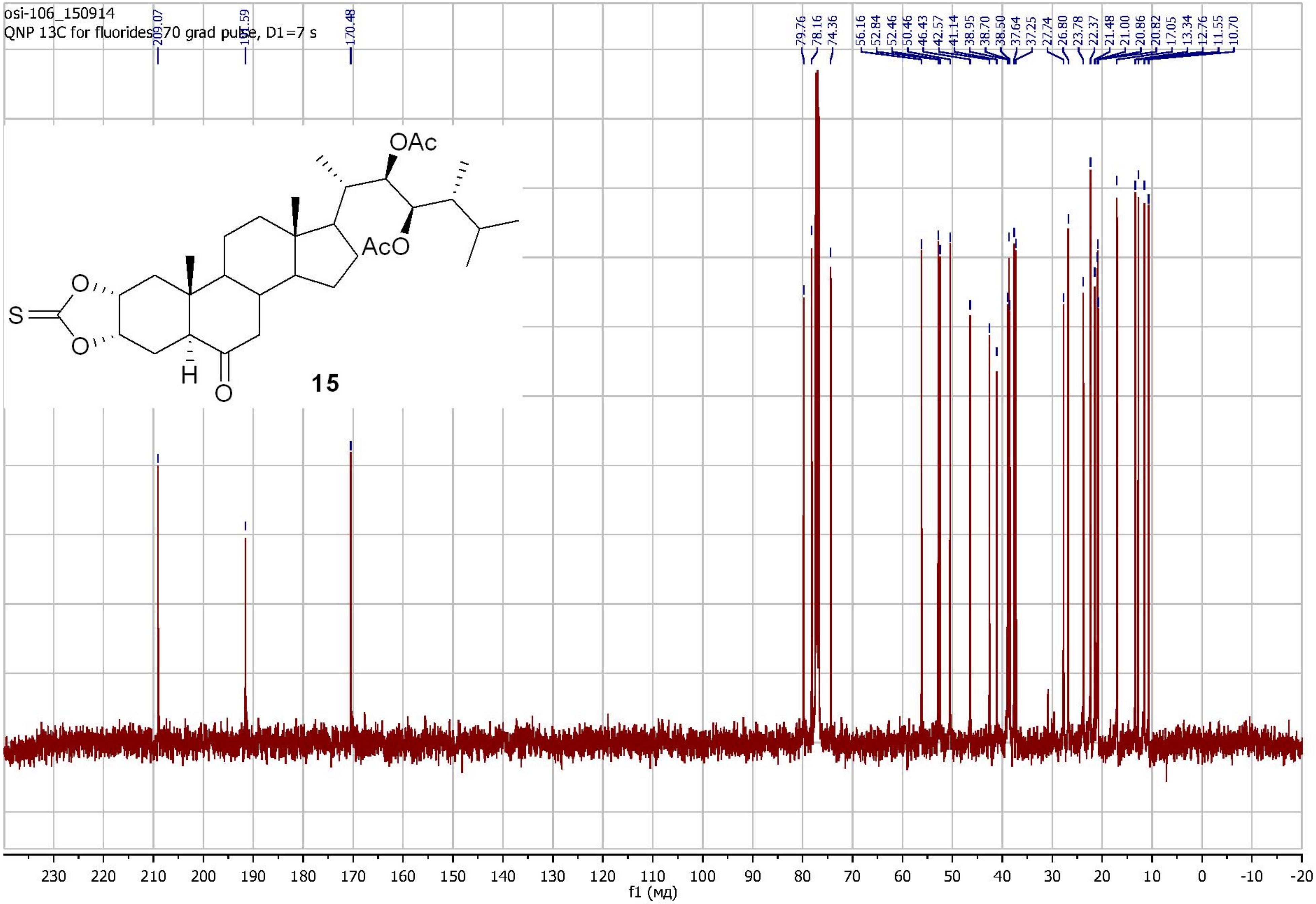

osi-106_150914
QNP 13C for fluorides 70 grad pulse, D1=7 s
209.07
170.48
79.76
78.16
74.36
56.16
52.84
52.46
50.46
46.43
42.57
41.14
38.95
38.70
38.50
37.64
37.25
27.74
26.80
23.78
22.37
21.48
21.00
20.86
20.82
17.05
13.34
12.76
11.55
10.70
OAc
AcO
S
O
O
H
O
15
230 220 210 200 190 180 170 160 150 140 130 120 110 100 90 80 70 60 50 40 30 20 10 0 -10 -20
f1 (мд)

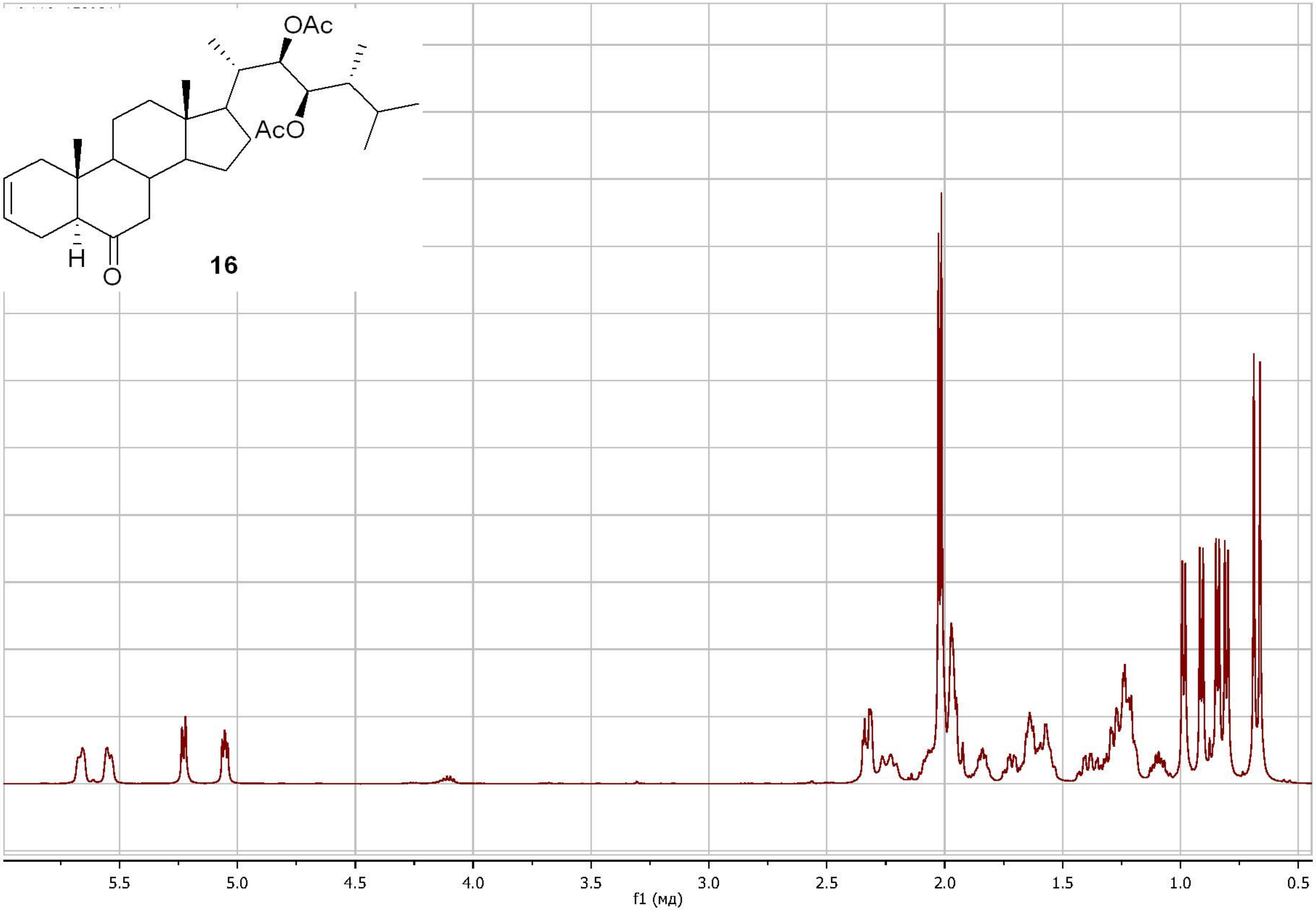
OAc
AcO
H
O
16
5.5
5.0
4.5
4.0
3.5
3.0
2.5
2.0
1.5
1.0
0.5
f1 (мд)

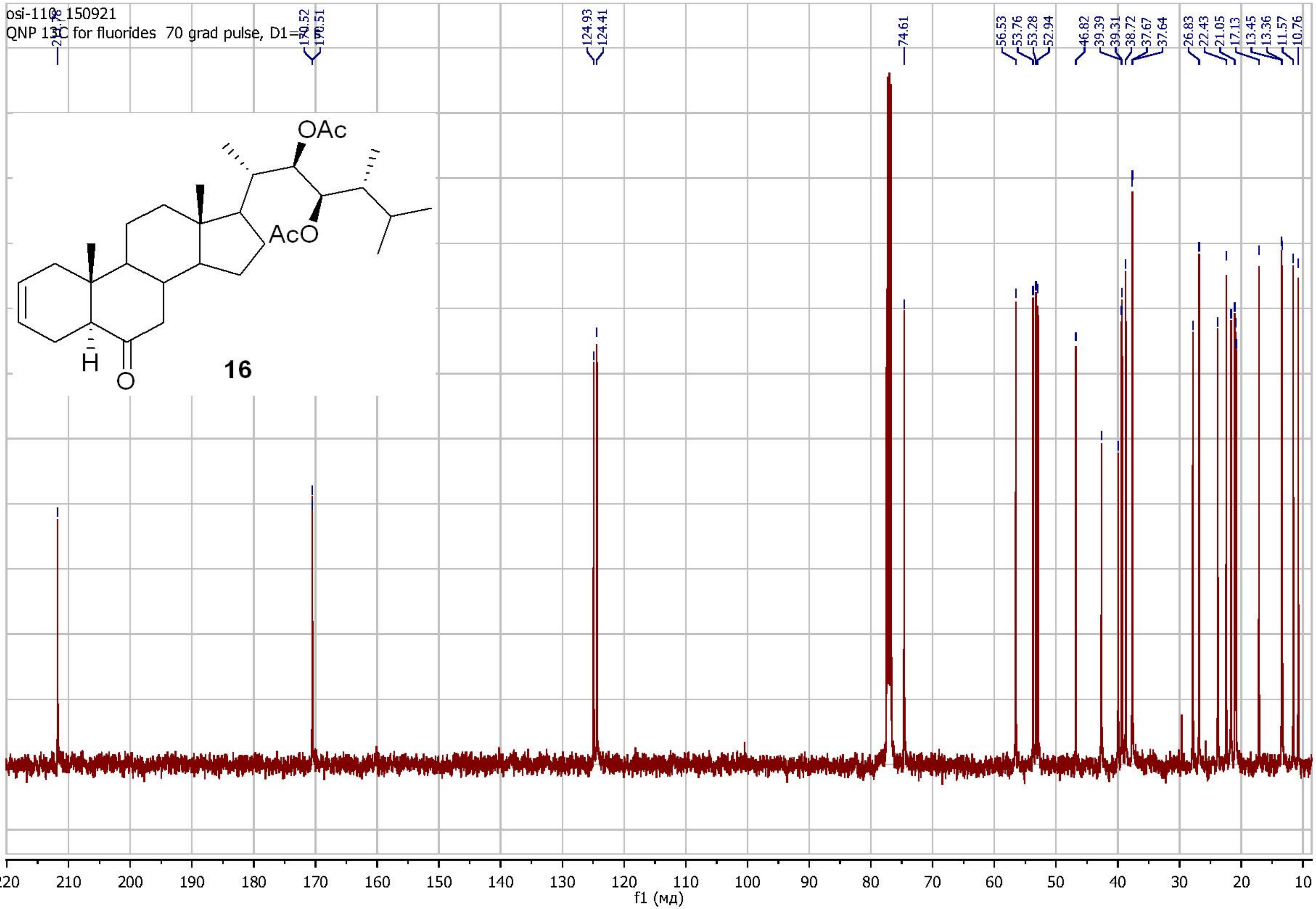

osi-110 150921
QNP 13C for fluorides 70 grad pulse, D1=
170.52
170.51
124.93
124.41
74.61
56.53
53.76
53.28
52.94
46.82
39.39
39.31
38.72
37.67
37.64
26.83
22.43
21.05
17.13
13.45
13.36
11.57
10.76
OAc
AcO
H
O
16
220 210 200 190 180 170 160 150 140 130 120 110 100 90 80 70 60 50 40 30 20 10
f1 (мд)

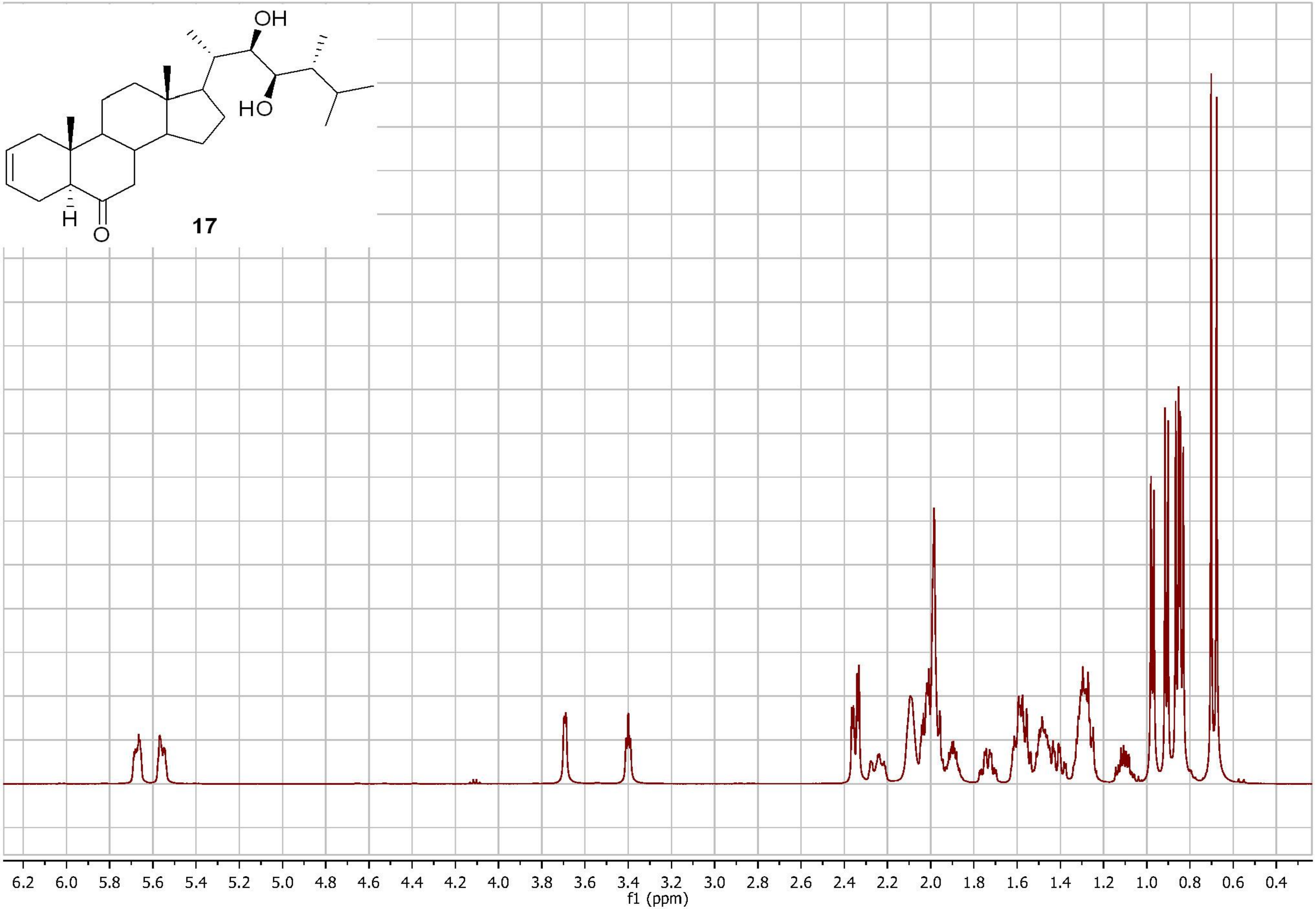
OH
HO
H
O
17
6.2 6.0 5.8 5.6 5.4 5.2 5.0 4.8 4.6 4.4 4.2 4.0 3.8 3.6 3.4 3.2 3.0 2.8 2.6 2.4 2.2 2.0 1.8 1.6 1.4 1.2 1.0 0.8 0.6 0.4
f1 (ppm)

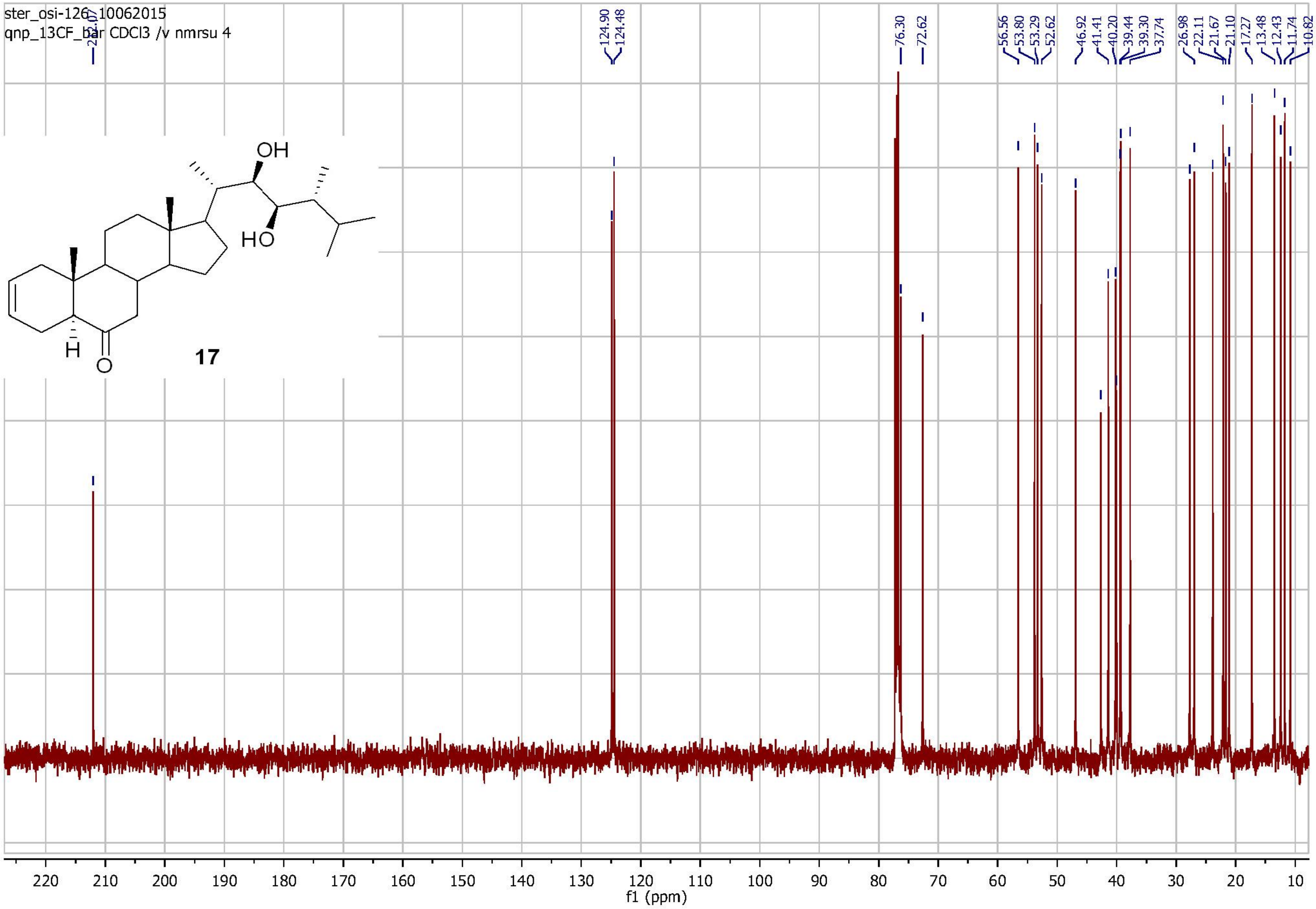
ster_osi-126_10062015
qnp_13CF_bar CDCl3 /v nmrsu 4
124.90
124.48
76.30
72.62
56.56
53.80
53.29
52.62
46.92
41.41
40.20
39.44
39.30
37.74
26.98
22.11
21.67
21.10
17.27
13.48
12.43
11.74
10.82
OH
HO
H
O
17
220
210
200
190
180
170
160
150
140
130
120
110
100
90
80
70
60
50
40
30
20
10
f1 (ppm)

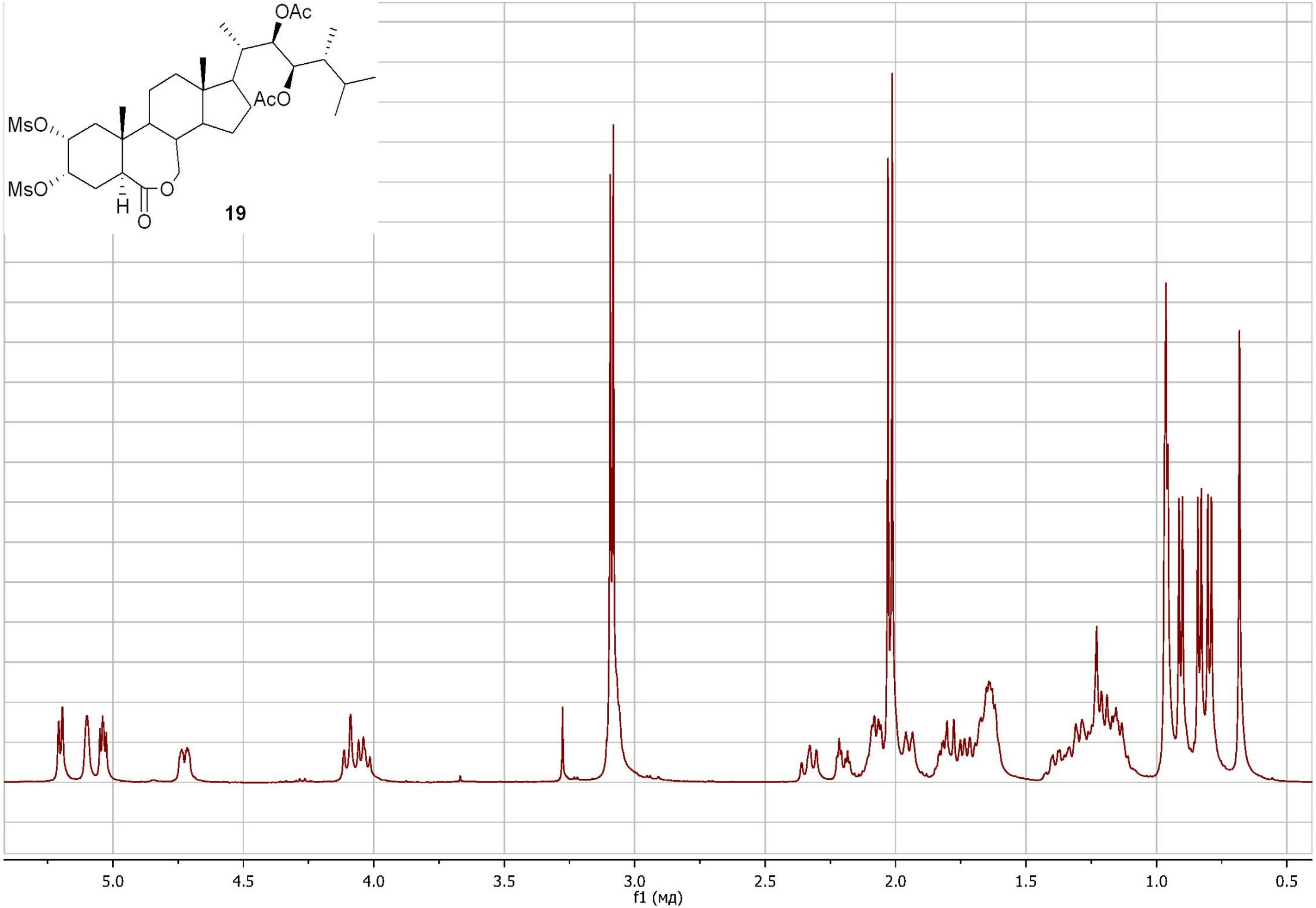
OAc
AcO
MsO
MsO
H
O
O
19
5.0
4.5
4.0
3.5
3.0
2.5
2.0
1.5
1.0
0.5
f1 (мд)

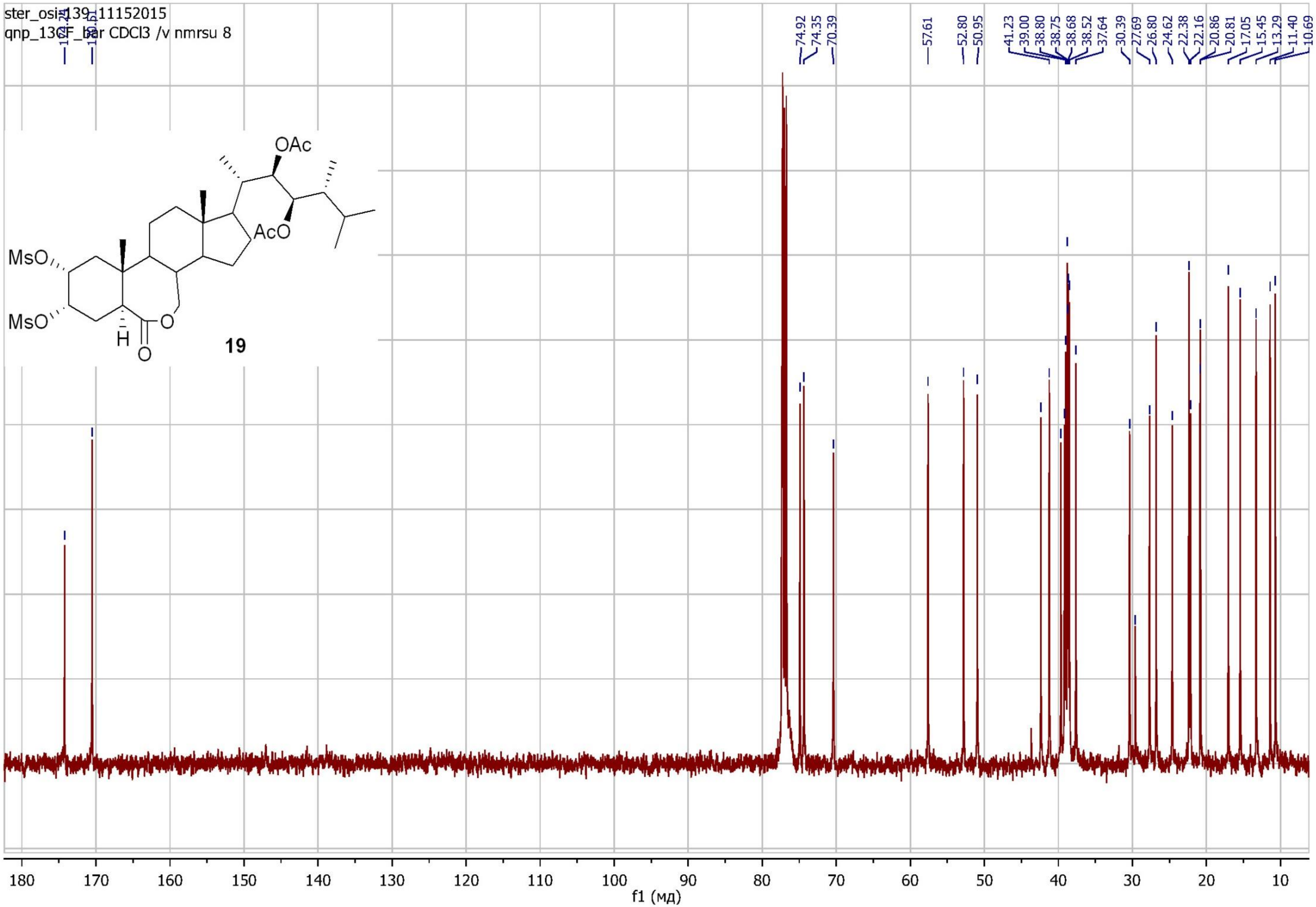
ster_osi-139_11152015
qnp_13CF_bar CDCl3 /v nmrsu 8
74.92
74.35
70.39
57.61
52.80
50.95
41.23
39.00
38.80
38.75
38.68
38.52
37.64
30.39
27.69
26.80
24.62
22.38
22.16
20.86
20.81
17.05
15.45
13.29
11.40
10.69
OAc
AcO
MsO
MsO
H
O
O
19
180 170 160 150 140 130 120 110 100 90 80 70 60 50 40 30 20 10
f1 (мд)

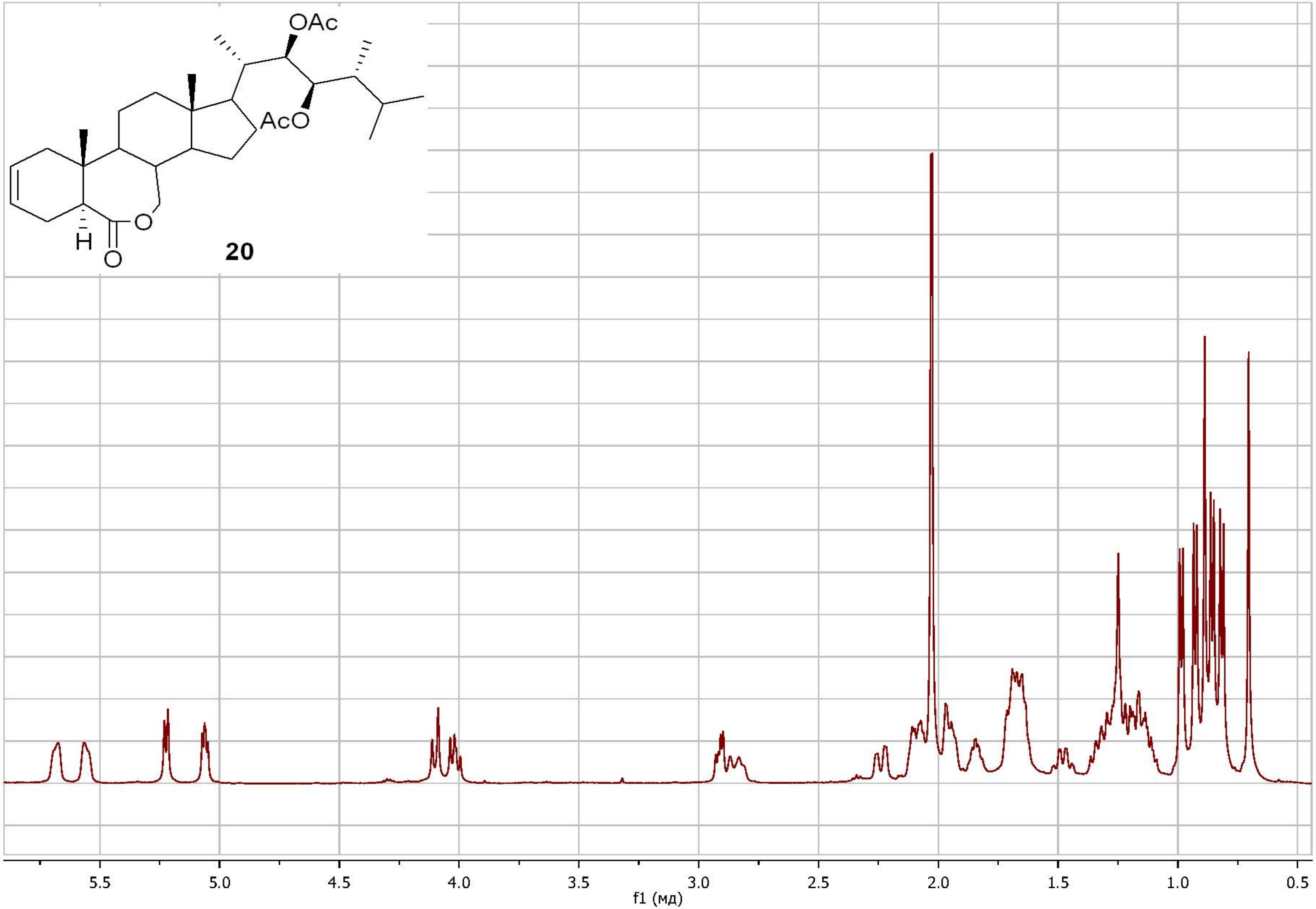
OAc
AcO
H
O
20
5.5
5.0
4.5
4.0
3.5
3.0
2.5
2.0
1.5
1.0
0.5
f1 (мд)

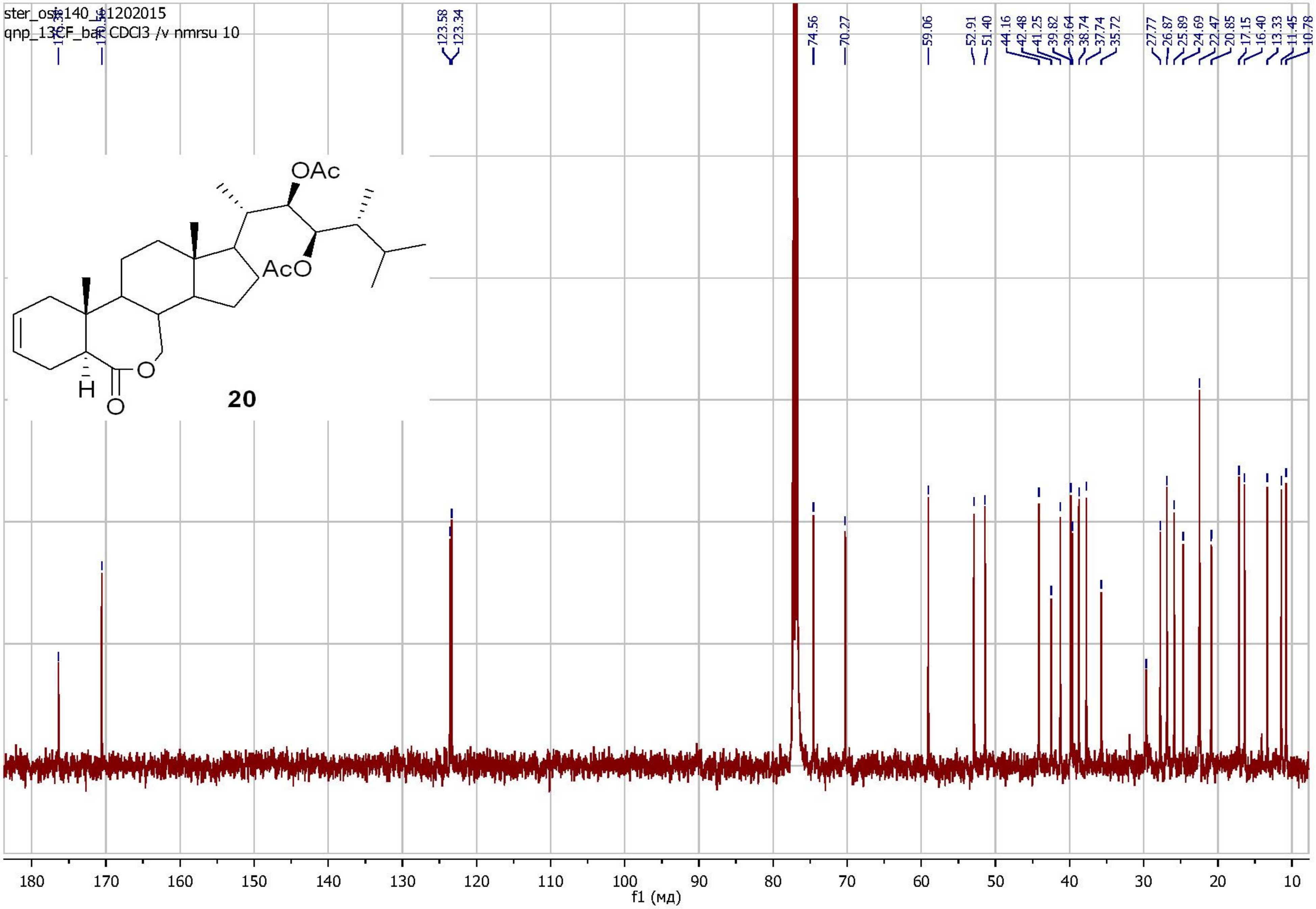

ster_osi-140_s1202015
qnp_13CF_bar CDCl3 /v nmrsu 10
123.58
123.34
74.56
70.27
59.06
52.91
51.40
44.16
42.48
41.25
39.82
39.64
38.74
37.74
35.72
27.77
26.87
25.89
24.69
22.47
20.85
17.15
16.40
13.33
11.45
10.78
OAc
AcO
H
O
20
180
170
160
150
140
130
120
110
100
90
80
70
60
50
40
30
20
10
f1 (мд)

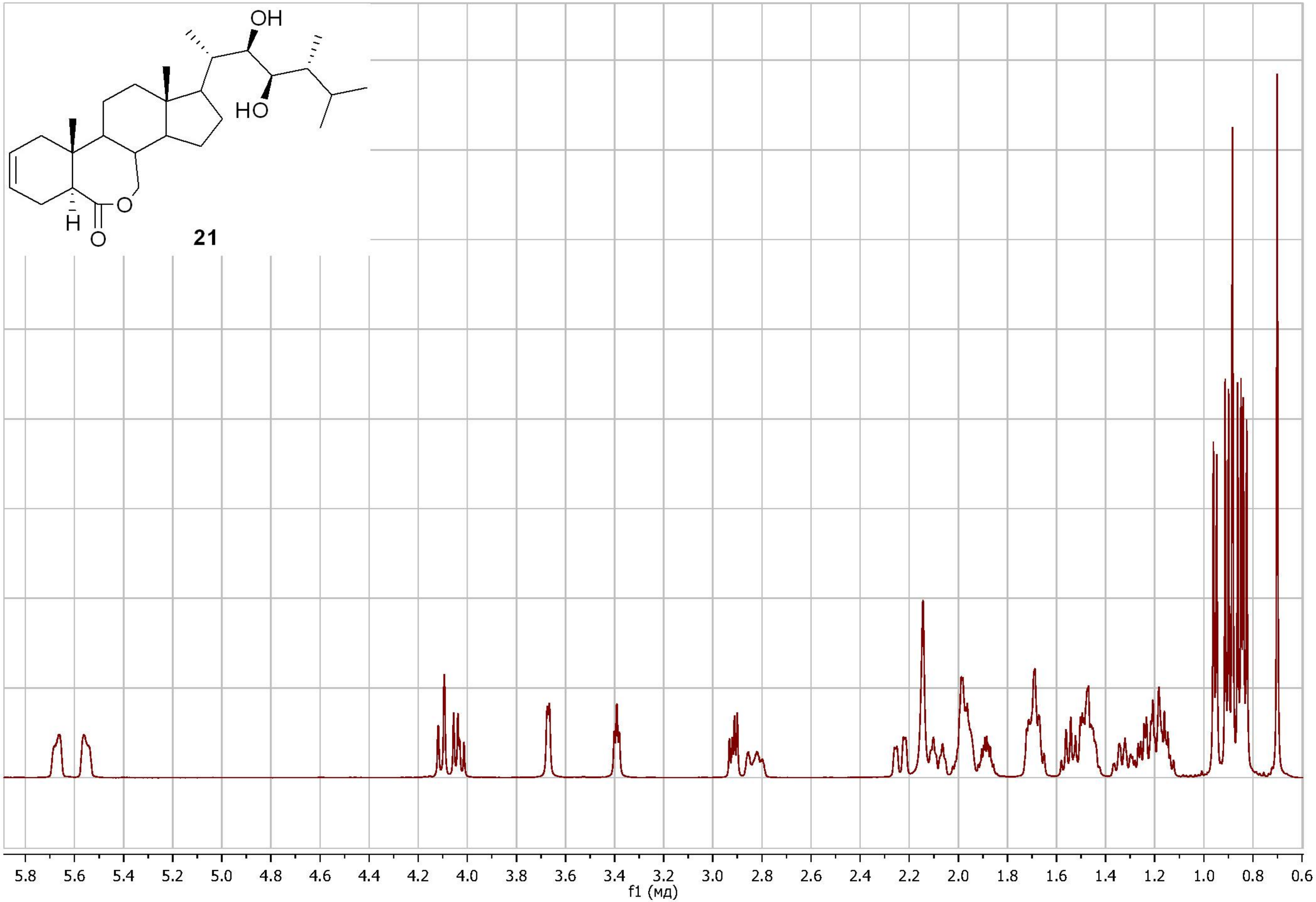
OH
HO
H
O
O
21
5.8 5.6 5.4 5.2 5.0 4.8 4.6 4.4 4.2 4.0 3.8 3.6 3.4 3.2 3.0 2.8 2.6 2.4 2.2 2.0 1.8 1.6 1.4 1.2 1.0 0.8 0.6
f1 (мд)

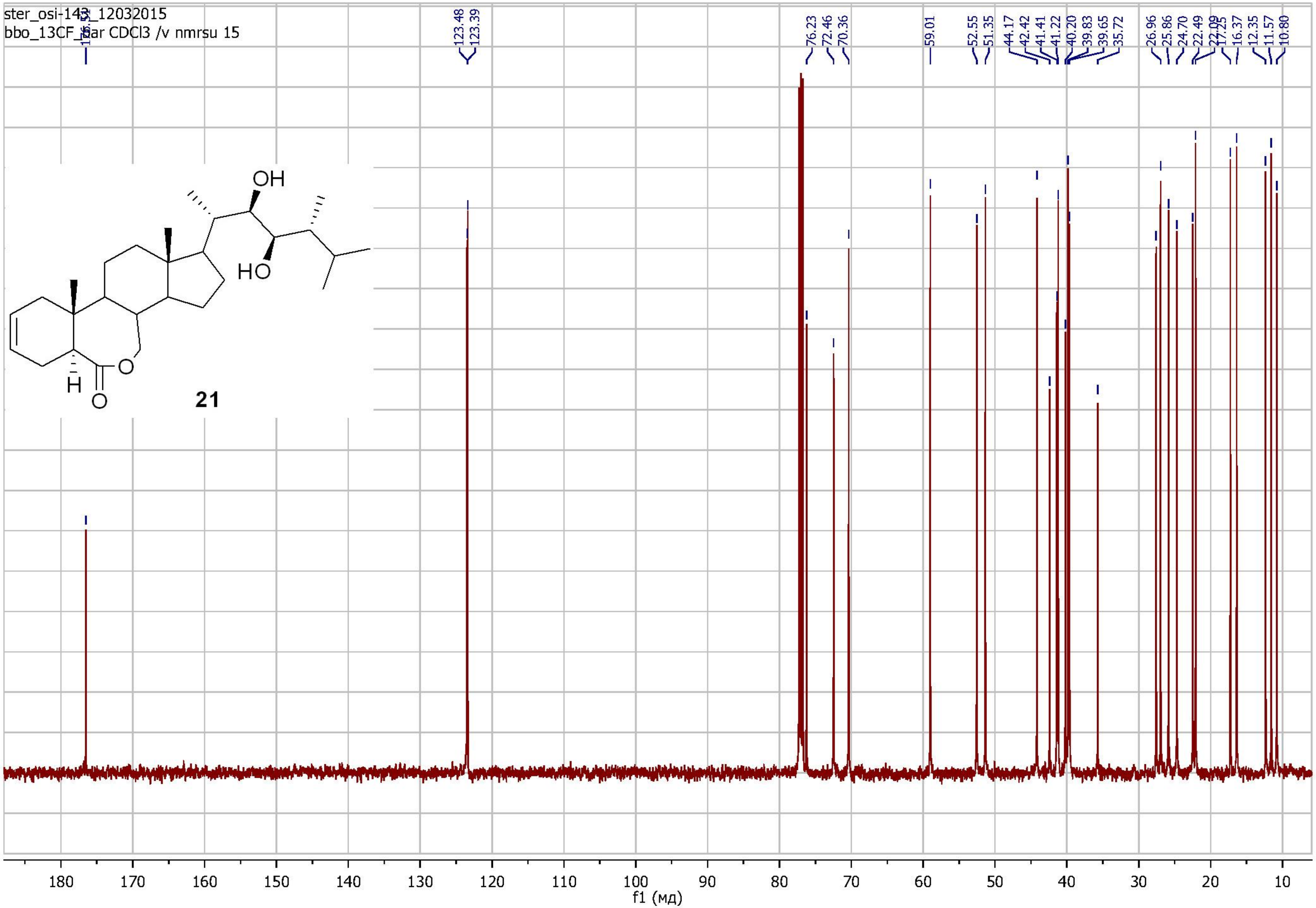
ster_osi-143_12032015
bbo_13CF_bar CDCl3 /v nmrsu 15
123.48
123.39
76.23
72.46
70.36
59.01
52.55
51.35
44.17
42.42
41.41
41.22
40.20
39.83
39.65
35.72
26.96
25.86
24.70
22.49
17.25
16.37
12.35
11.57
10.80
OH
HO
H
O
21
180 170 160 150 140 130 120 110 100 90 80 70 60 50 40 30 20 10
f1 (мд)

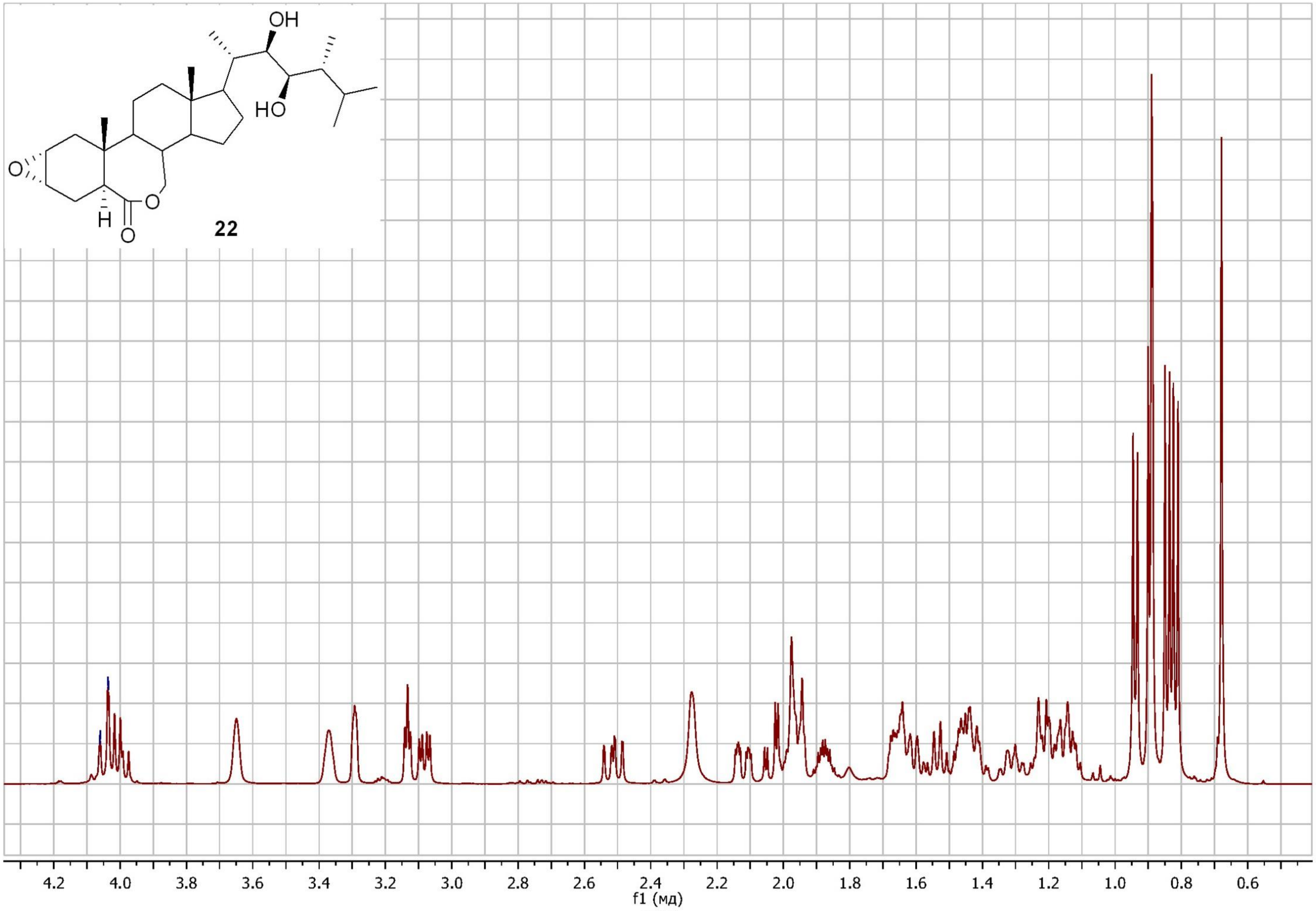
OH
HO
O
H
O
O
22
4.2
4.0
3.8
3.6
3.4
3.2
3.0
2.8
2.6
2.4
2.2
2.0
1.8
1.6
1.4
1.2
1.0
0.8
0.6
f1 (мд)

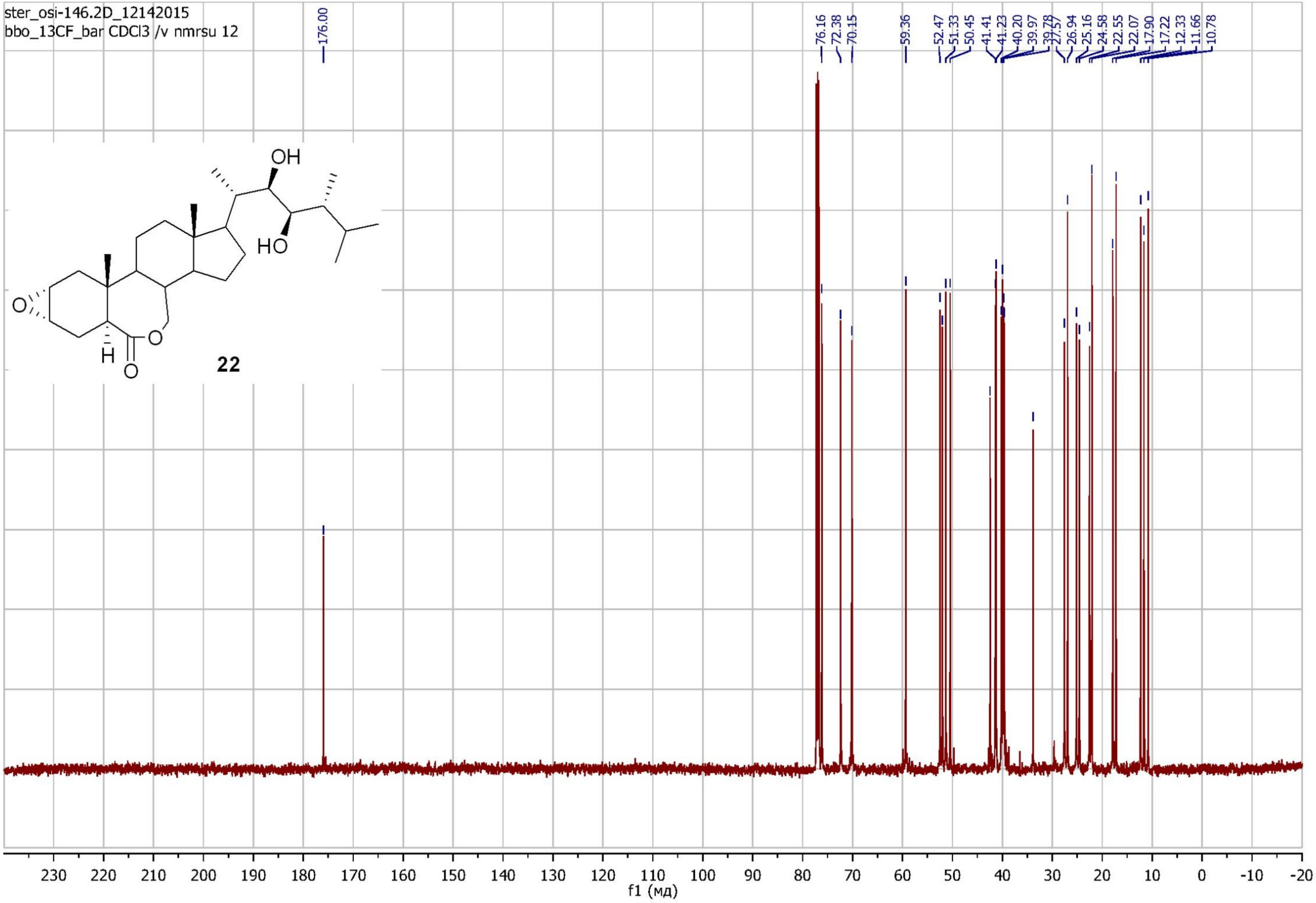
ster_osi-146.2D_12142015
bbo_13CF_bar CDCl3 /v nmrsu 12
176.00
76.16
72.38
70.15
59.36
52.47
51.33
50.45
41.41
41.23
40.20
39.97
39.78
27.57
26.94
25.16
24.58
22.55
22.07
17.90
17.22
12.33
11.66
10.78
OH
HO
O
H
O
O
22
230 220 210 200 190 180 170 160 150 140 130 120 110 100 90 80 70 60 50 40 30 20 10 0 -10 -20
f1 (мд)

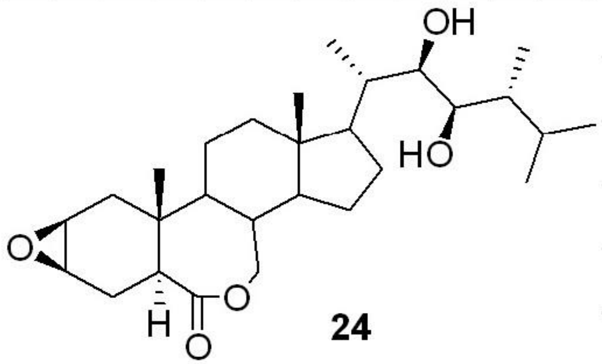

24

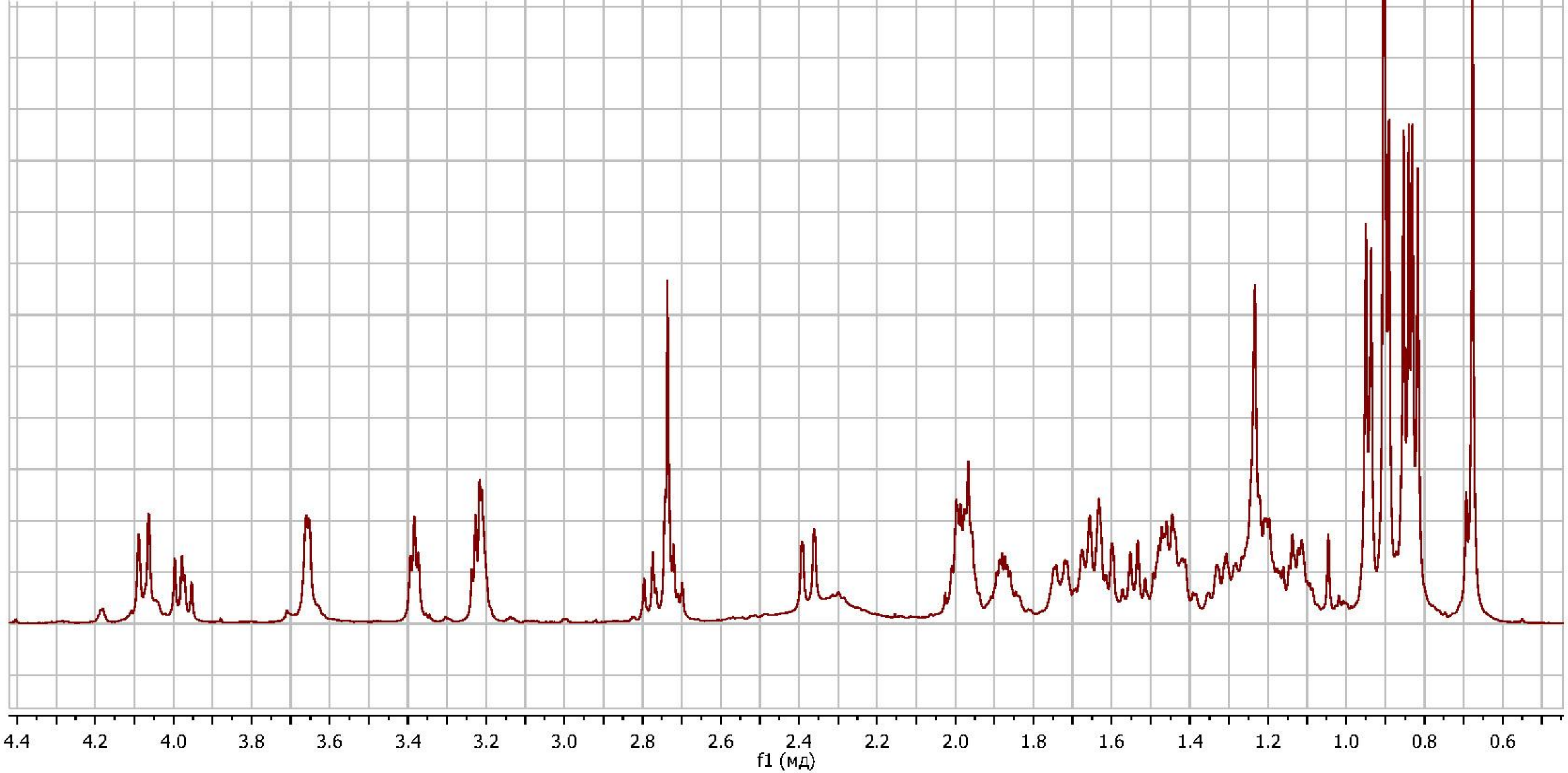

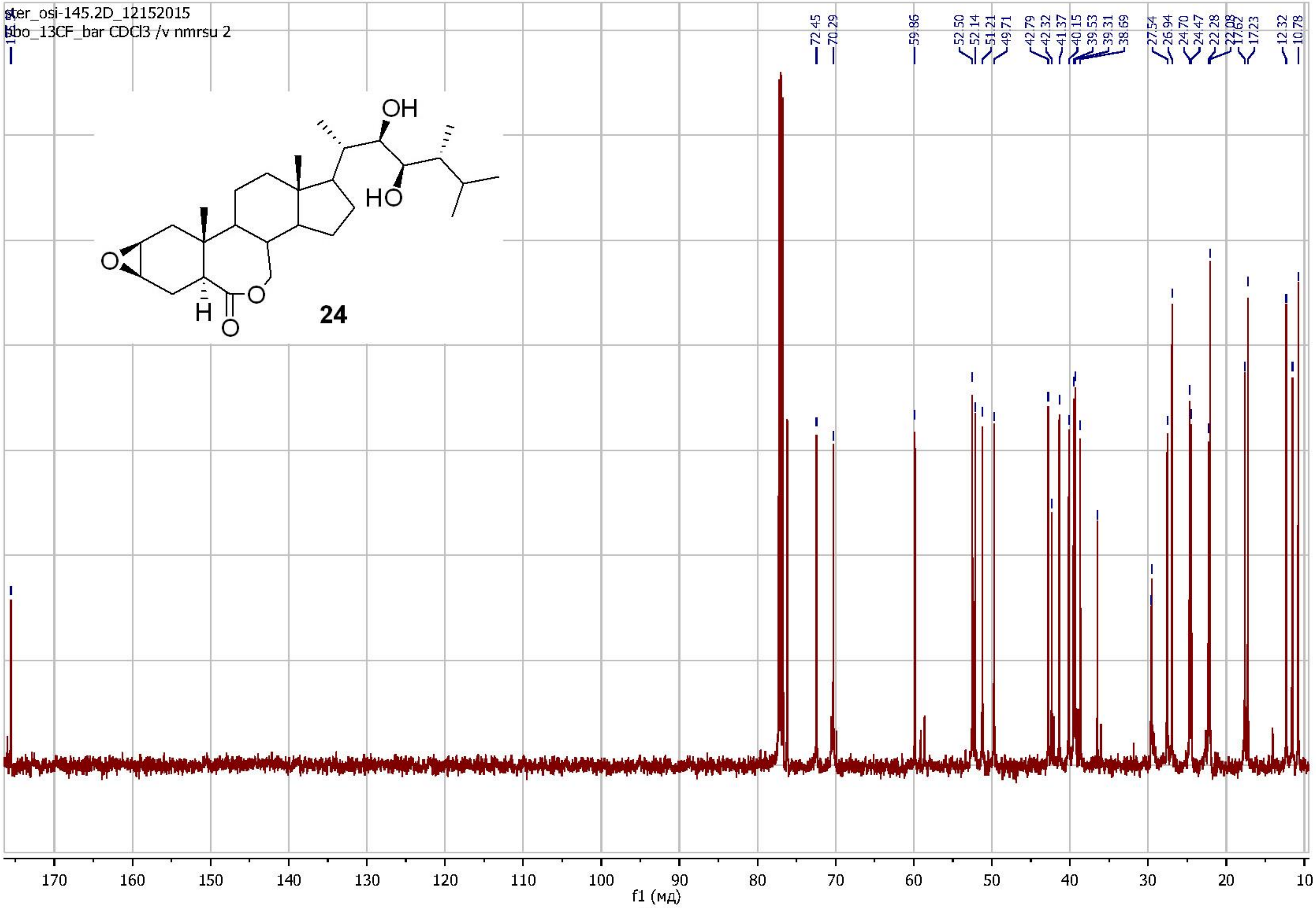
ster_osi-145.2D_12152015
bbo_13CF_bar CDCl3 /v nmrsu 2
72.45
70.29
59.86
52.50
52.14
51.21
49.71
42.79
42.32
41.37
40.15
39.53
39.31
38.69
27.54
26.94
24.70
24.47
22.28
22.08
17.62
17.23
12.32
10.78
OH
HO
O
H
O
O
24
170
160
150
140
130
120
110
100
90
80
70
60
50
40
30
20
10
f1 (мд)

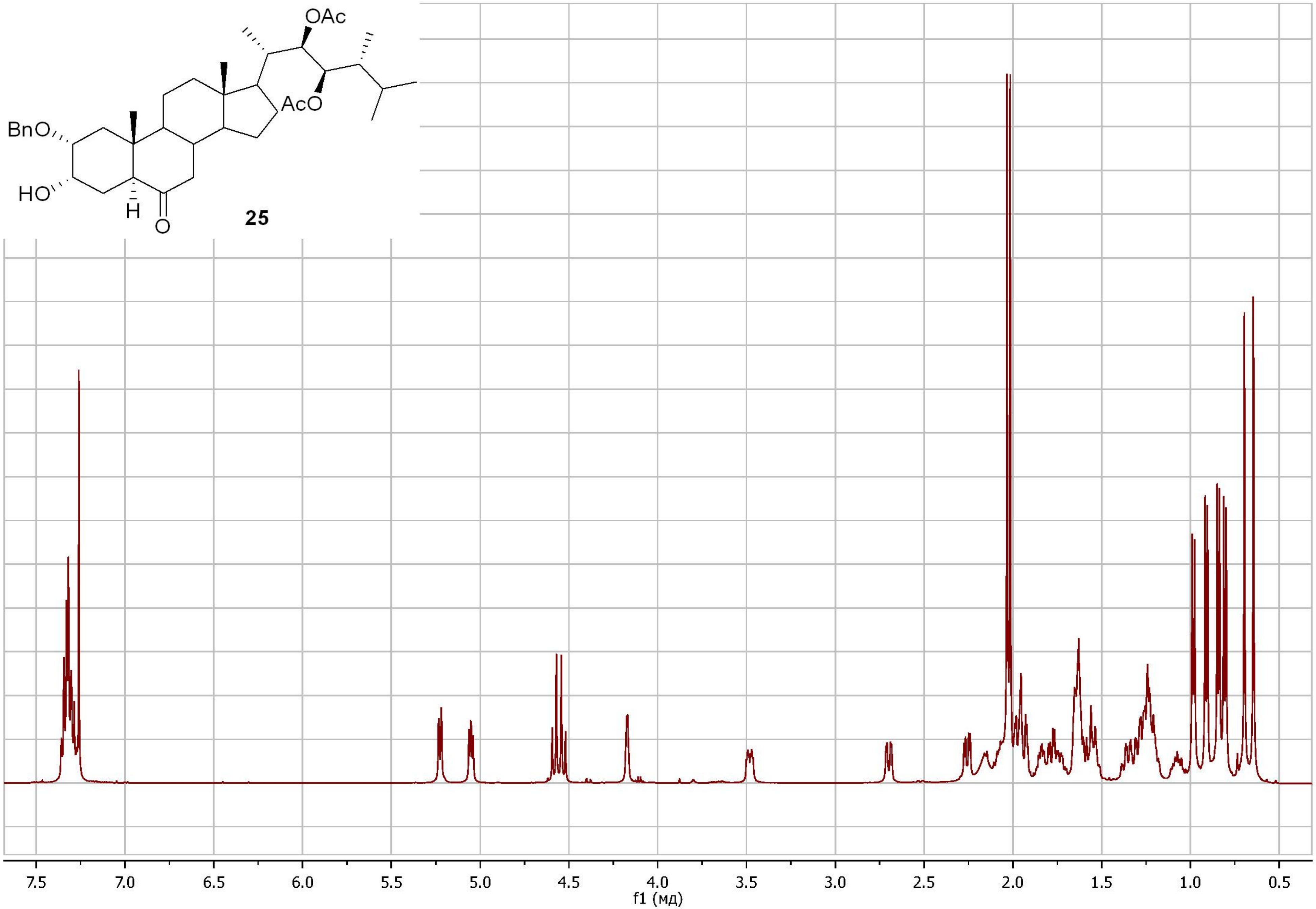
OAc
AcO
BnO
HO
H
O
25
7.5
7.0
6.5
6.0
5.5
5.0
4.5
4.0
3.5
3.0
2.5
2.0
1.5
1.0
0.5
f1 (мд)

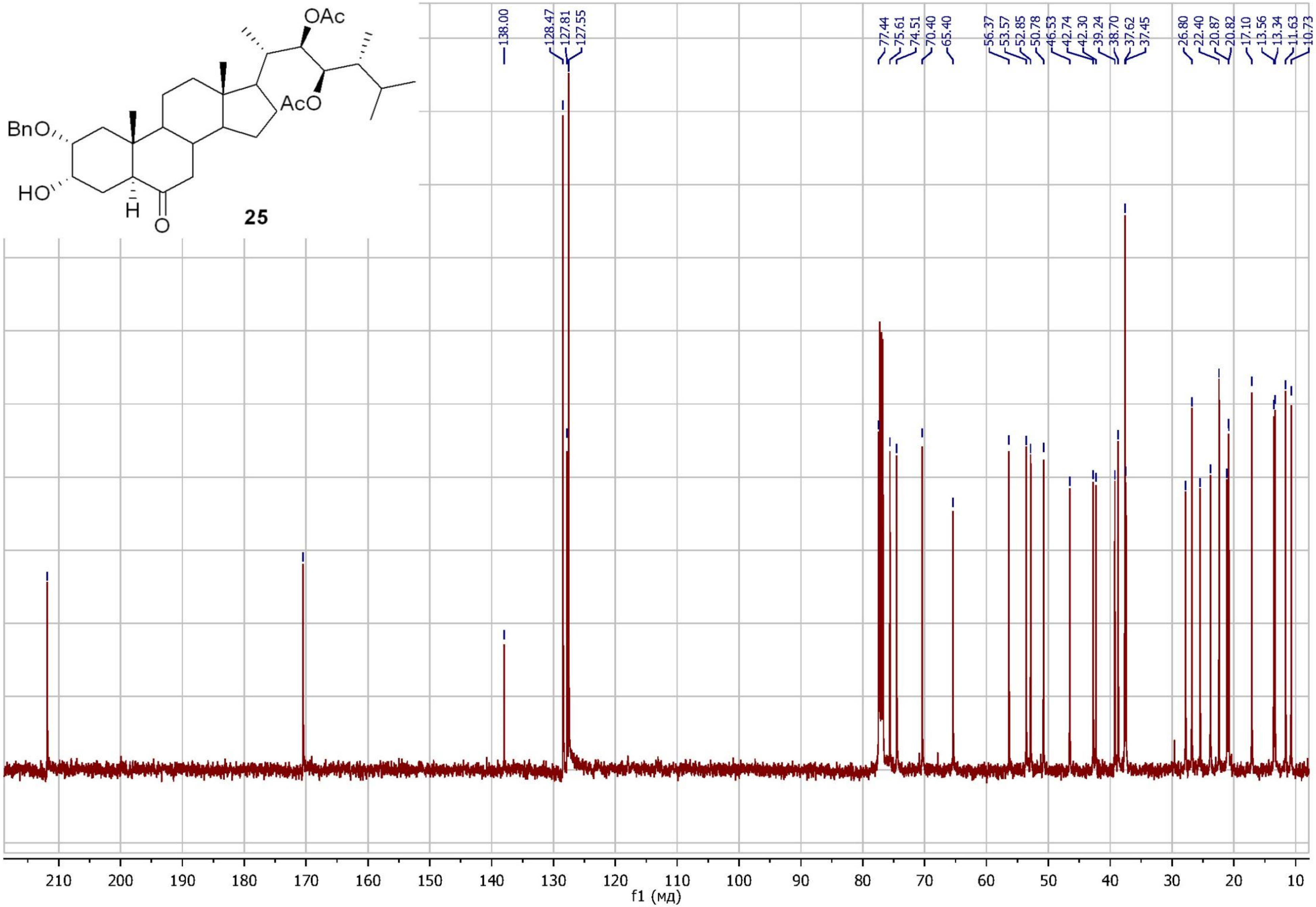
OAc
BnO
AcO
HO
H
O
25
138.00
128.47
127.81
127.55
77.44
75.61
74.51
70.40
65.40
56.37
53.57
52.85
50.78
46.53
42.74
42.30
39.24
38.70
37.62
37.45
26.80
22.40
20.87
20.82
17.10
13.56
13.34
11.63
10.73
210 200 190 180 170 160 150 140 130 120 110 100 90 80 70 60 50 40 30 20 10
f1 (мд)

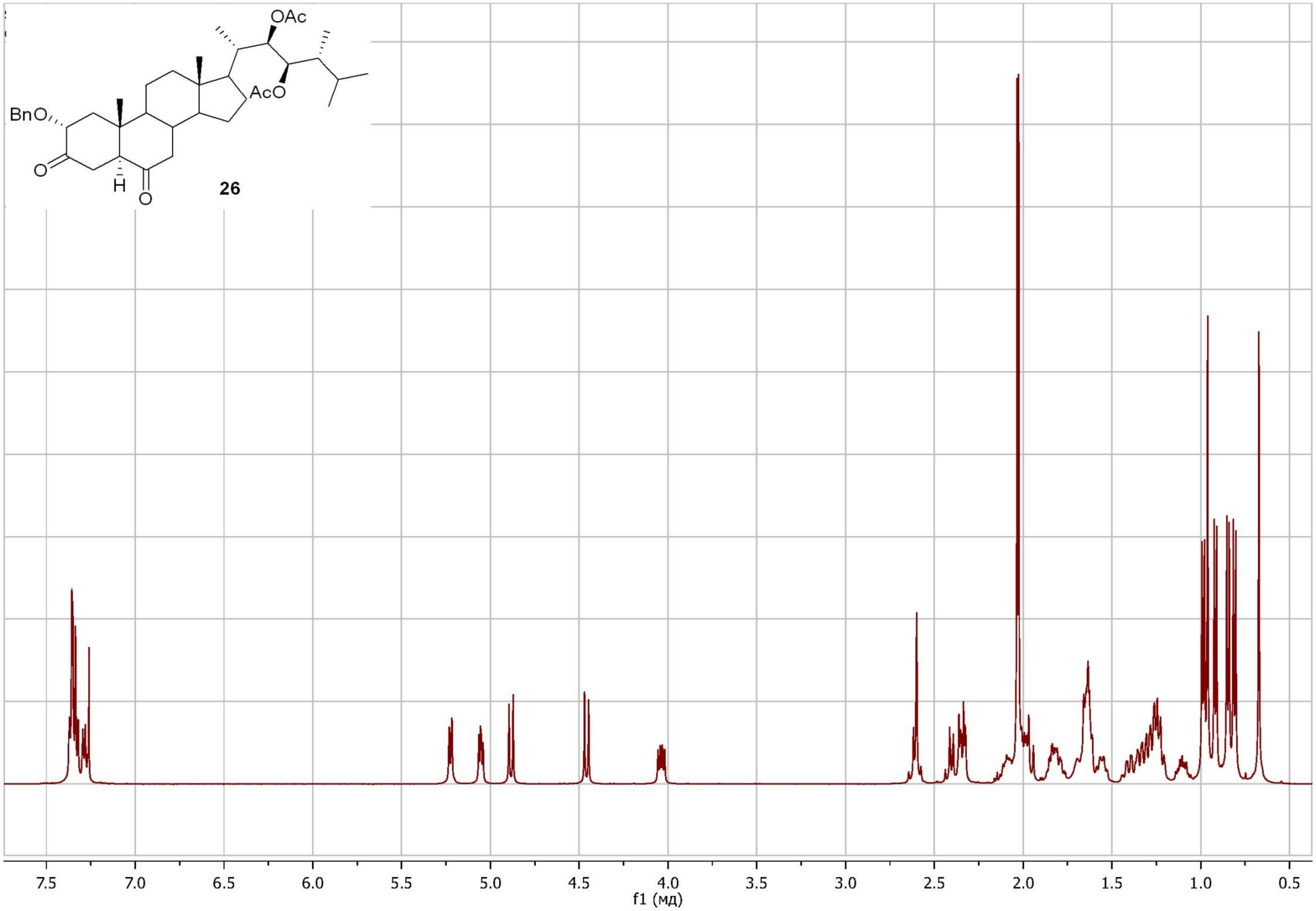
OAc
AcO
BnO
O
H
O
26
7.5
7.0
6.5
6.0
5.5
5.0
4.5
4.0
3.5
3.0
2.5
2.0
1.5
1.0
0.5
f1 (мд)

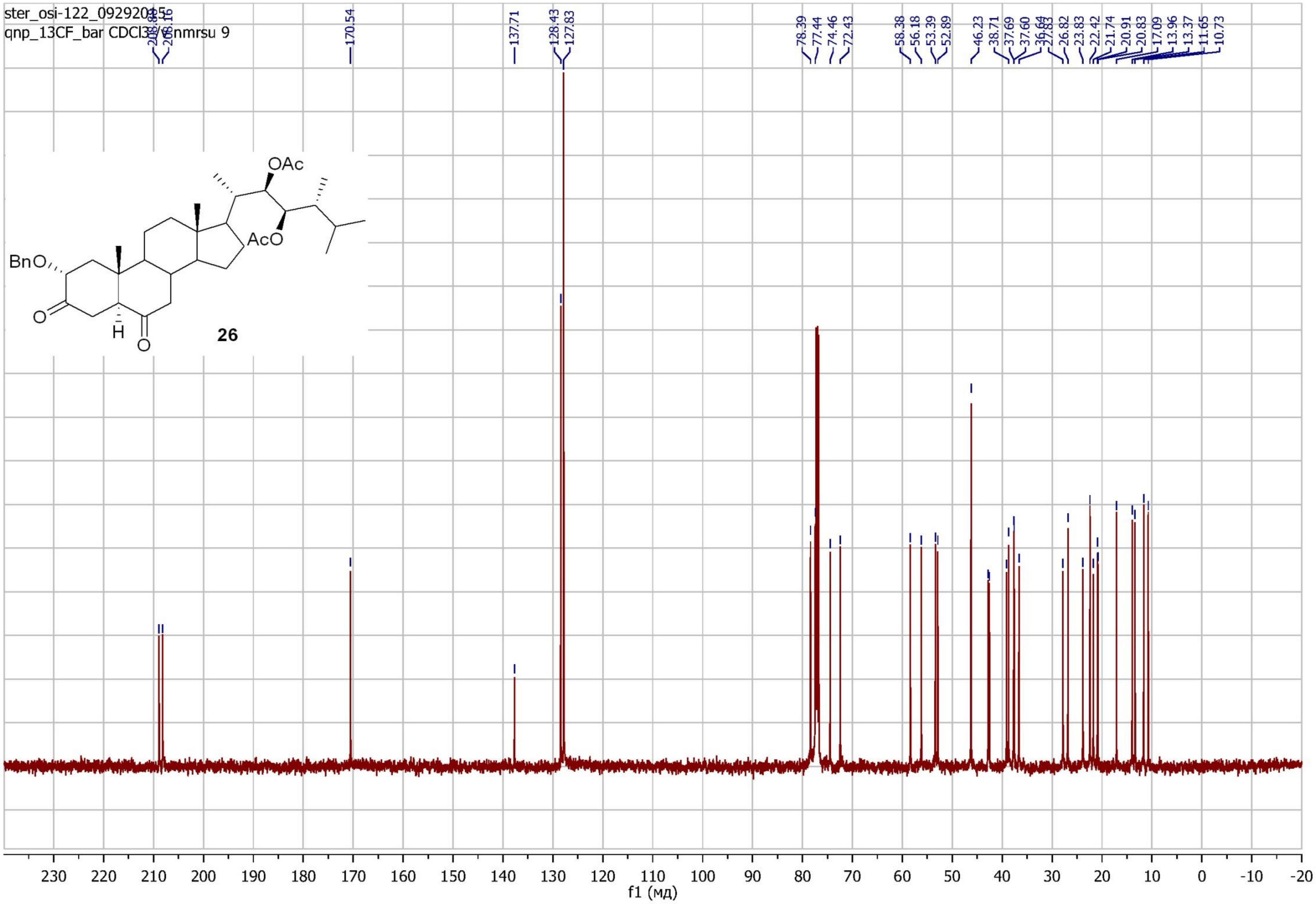
ster_osi-122_09292015
qnp_13CF_bar CDCl3/vnmrsu 9
208.16
170.54
137.71
128.43
127.83
78.39
77.44
74.46
72.43
58.38
56.18
53.39
52.89
46.23
38.71
37.69
37.60
27.83
26.82
23.83
22.42
21.74
20.91
20.83
17.09
13.96
13.37
11.65
10.73
OAc
AcO
BnO
O
H
O
26
230
220
210
200
190
180
170
160
150
140
130
120
110
100
90
80
70
60
50
40
30
20
10
0
-10
-20
f1 (мд)

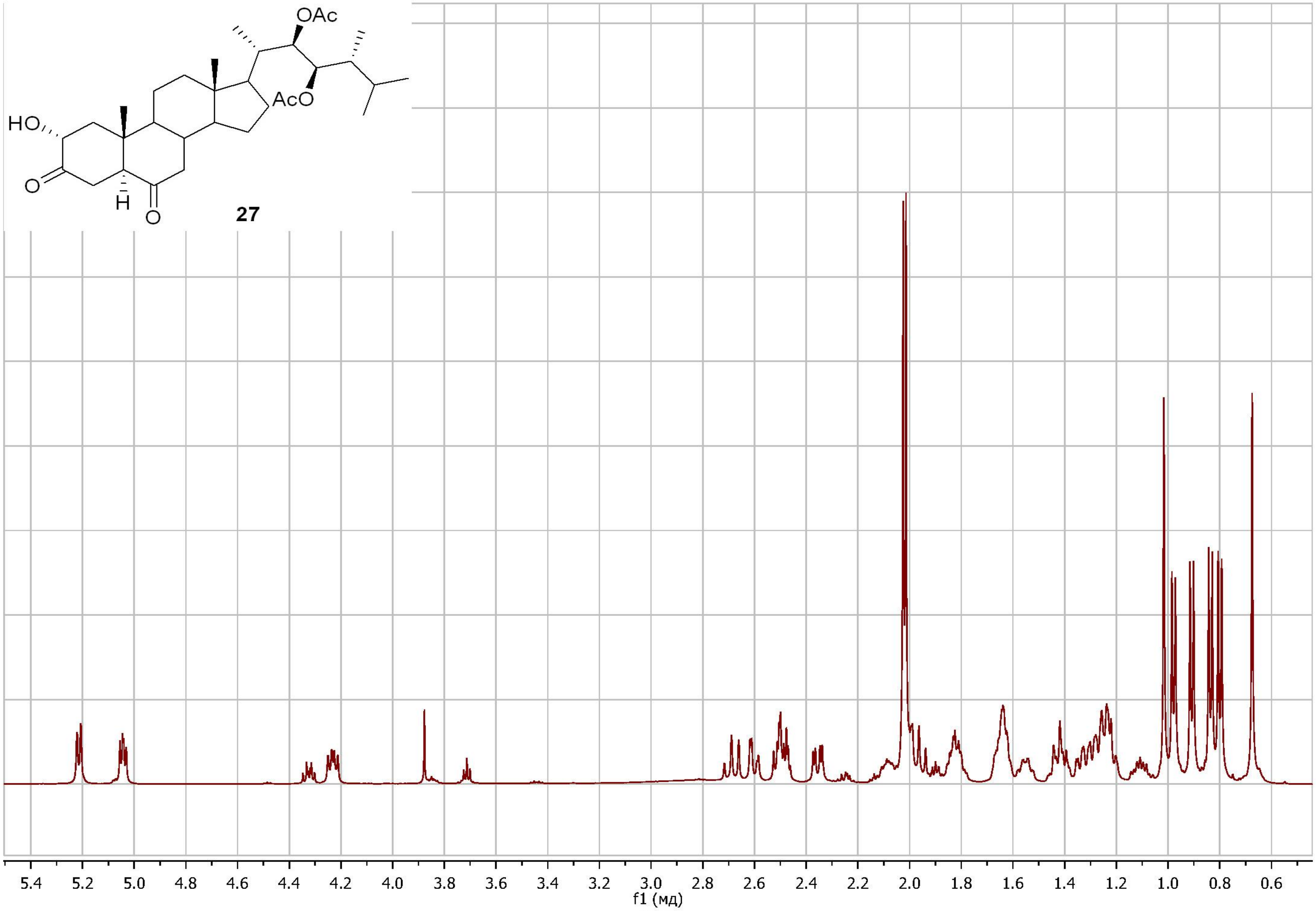
OAc
AcO
HO
O
H
O
27
5.4
5.2
5.0
4.8
4.6
4.4
4.2
4.0
3.8
3.6
3.4
3.2
3.0
2.8
2.6
2.4
2.2
2.0
1.8
1.6
1.4
1.2
1.0
0.8
0.6
f1 (мд)

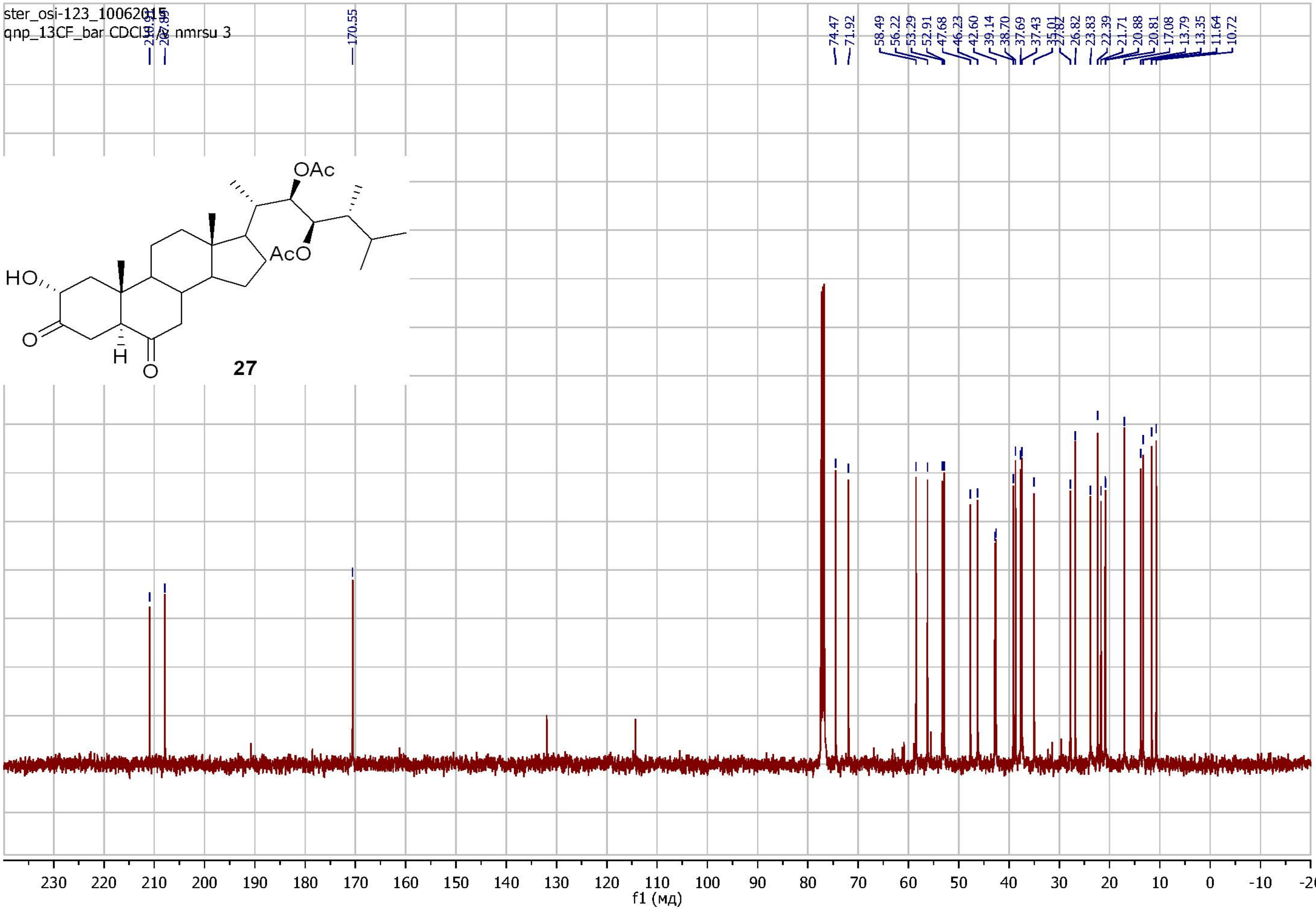
ster_osi-123_10062015
qnp_13CF_bar CDCl3 nmrsu 3
170.55
74.47
71.92
58.49
56.22
53.29
52.91
47.68
46.23
42.60
39.14
38.70
37.69
37.43
35.01
27.82
26.82
23.83
22.39
21.71
20.88
20.81
17.08
13.79
13.35
11.64
10.72
OAc
AcO
HO
O
H
O
27
230 220 210 200 190 180 170 160 150 140 130 120 110 100 90 80 70 60 50 40 30 20 10 0 -10 -20
f1 (мд)

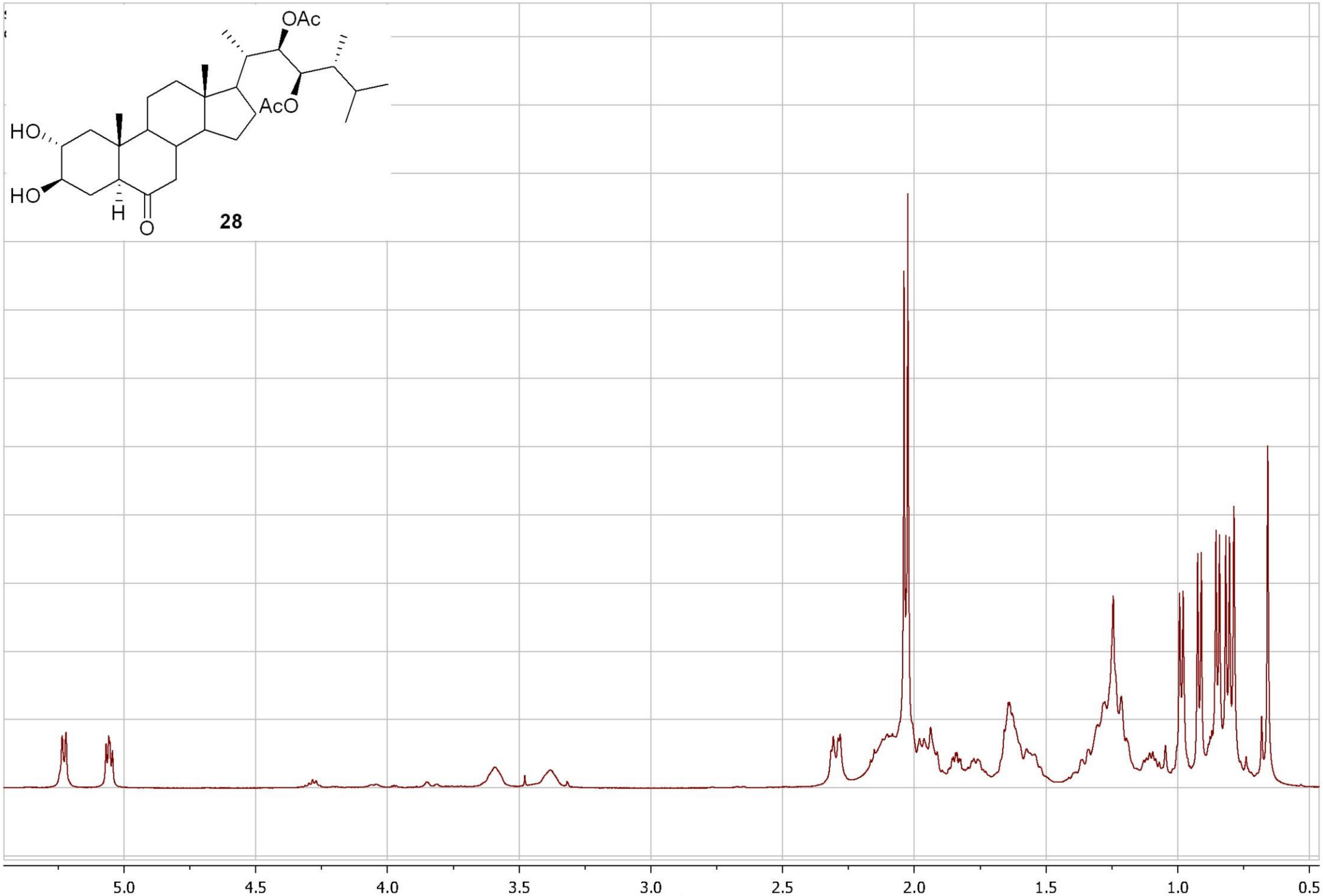
OAc
AcO
HO
HO
H
O
28
5.0
4.5
4.0
3.5
3.0
2.5
2.0
1.5
1.0
0.5
f1 (мд)

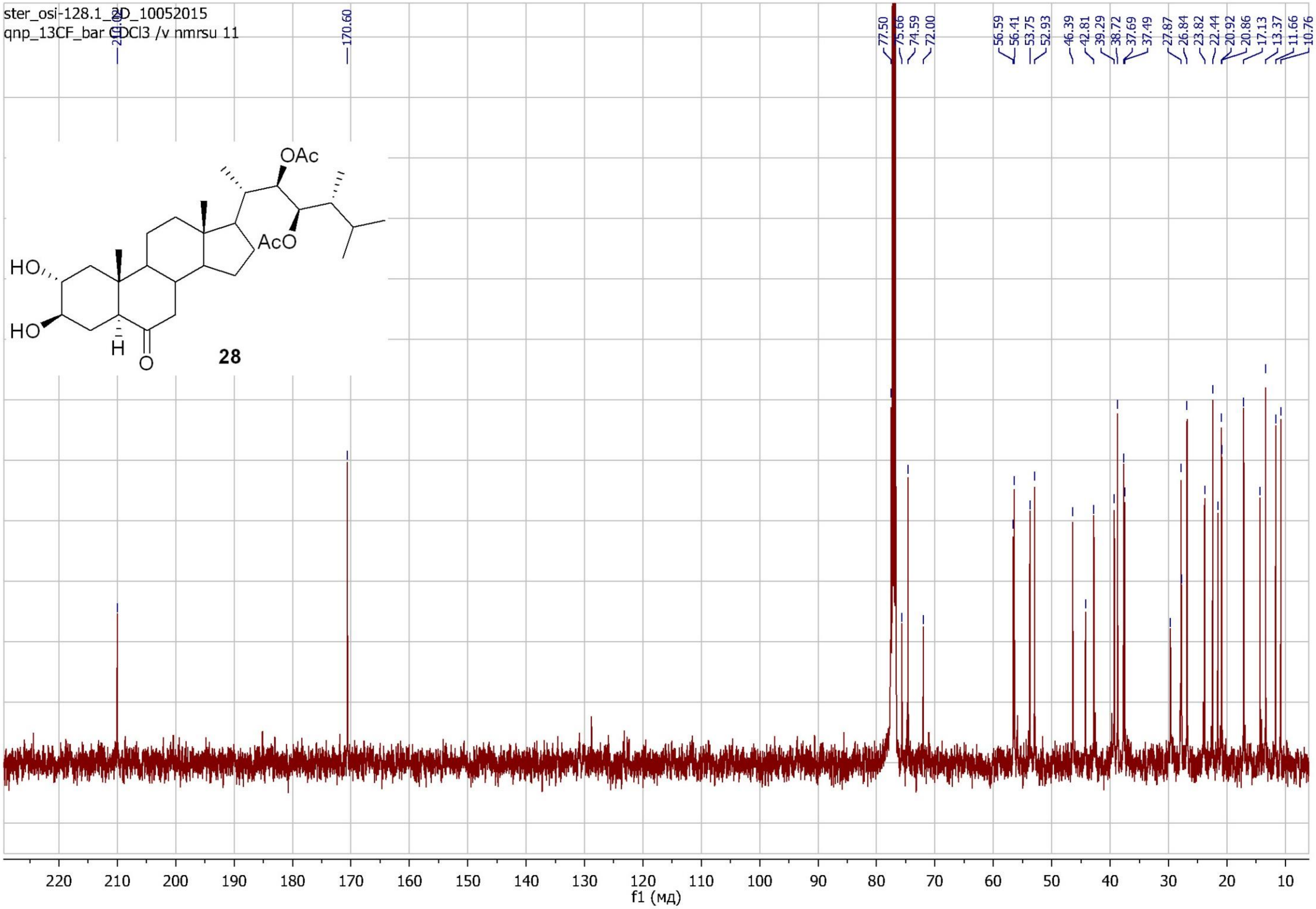
ster_osi-128.1_2D_10052015
qnp_13CF_bar CDCl3 /v nmrsu 11
170.60
77.50
75.66
74.59
72.00
56.59
56.41
53.75
52.93
46.39
42.81
39.29
38.72
37.69
37.49
27.87
26.84
23.82
22.44
20.92
20.86
17.13
13.37
11.66
10.76
OAc
AcO
HO
HO
H
O
28
220
210
200
190
180
170
160
150
140
130
120
110
100
90
80
70
60
50
40
30
20
10
f1 (мд)

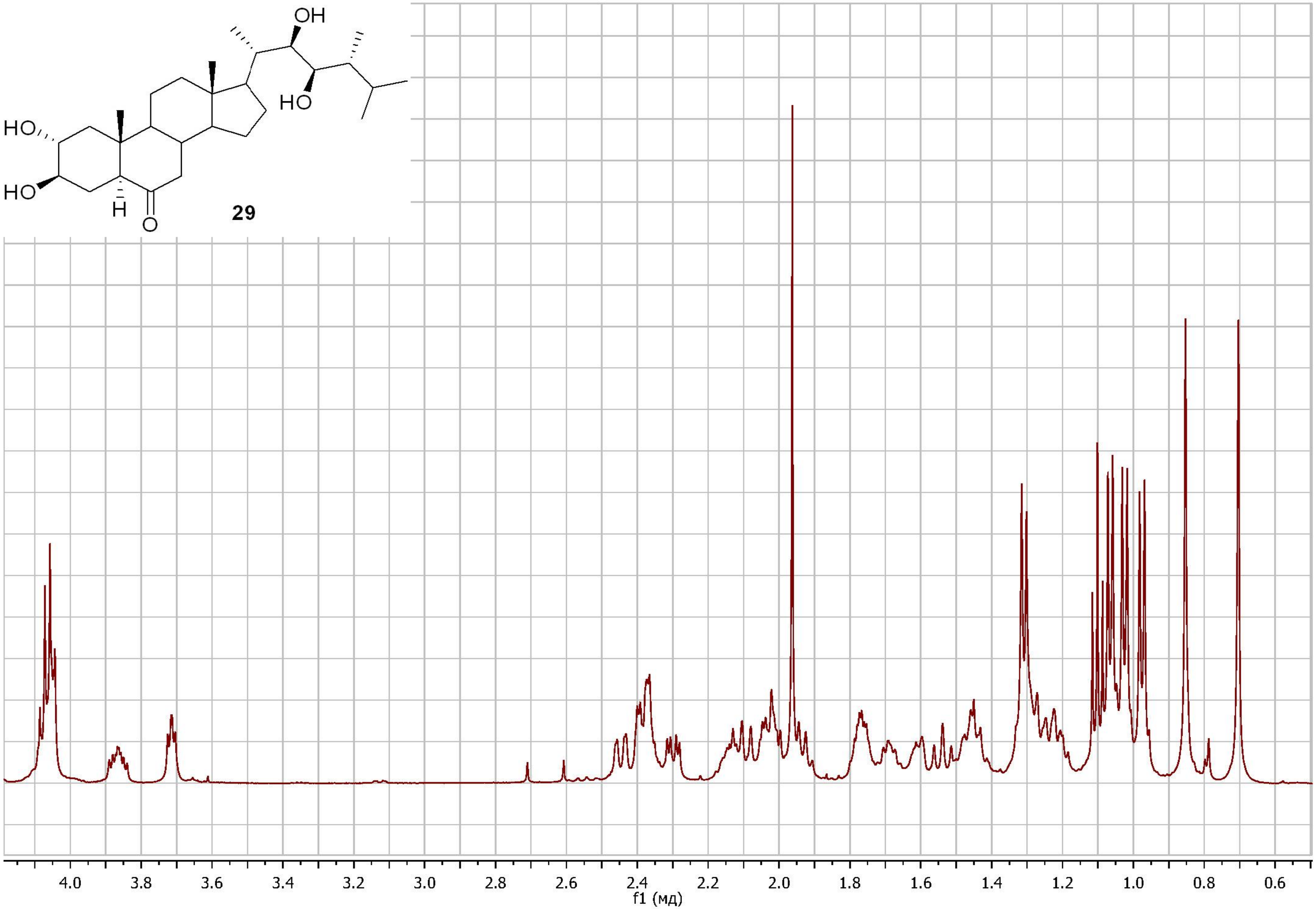
OH
HO
HO
HO
H
O
29
4.0
3.8
3.6
3.4
3.2
3.0
2.8
2.6
2.4
2.2
2.0
1.8
1.6
1.4
1.2
1.0
0.8
0.6
f1 (мд)

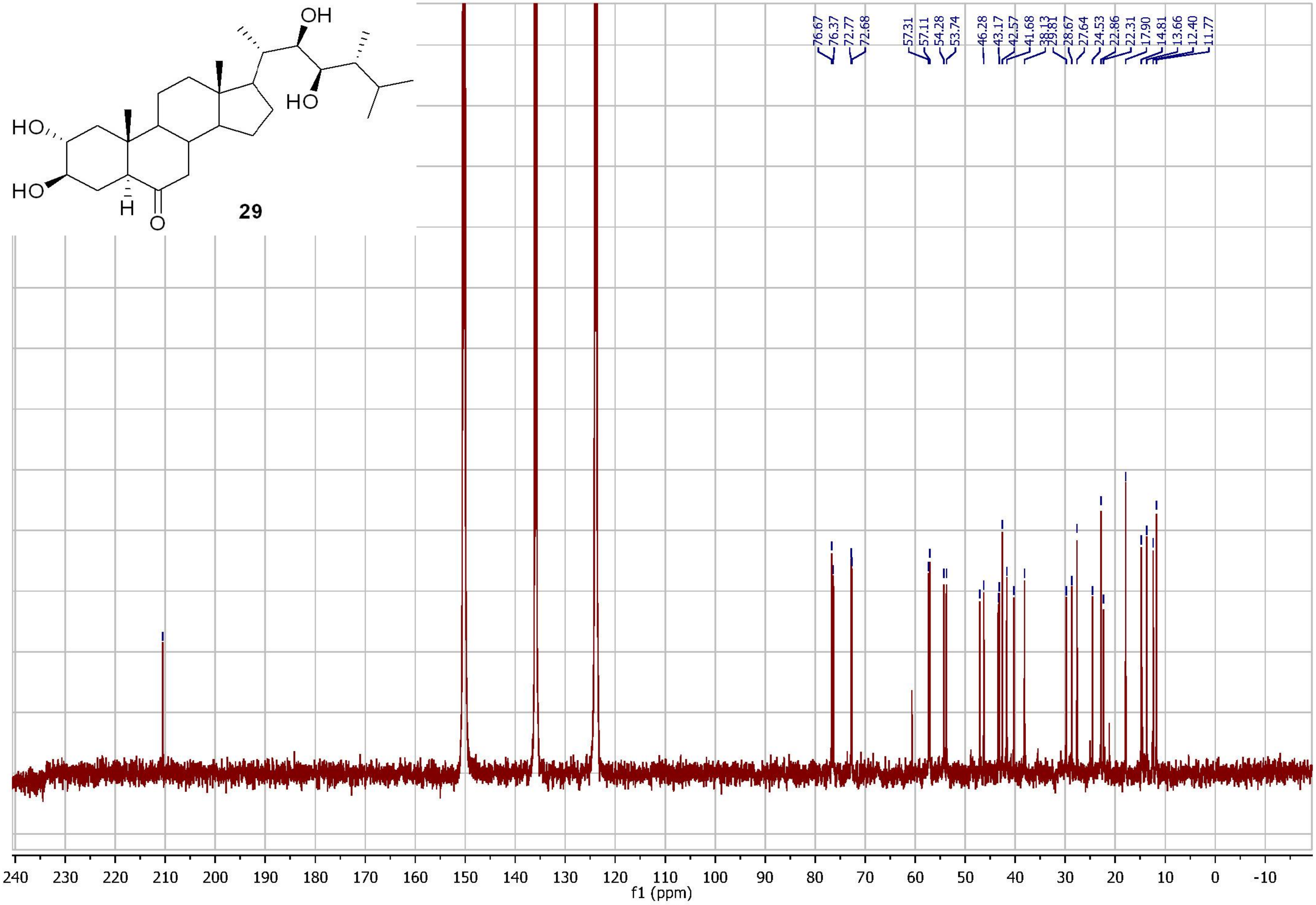
OH
HO
HO
HO
H
O
29
76.67
76.37
72.77
72.68
57.31
57.11
54.28
53.74
46.28
43.17
42.57
41.68
38.13
29.81
28.67
27.64
24.53
22.86
22.31
17.90
14.81
13.66
12.40
11.77
240 230 220 210 200 190 180 170 160 150 140 130 120 110 100 90 80 70 60 50 40 30 20 10 0 -10
f1 (ppm)

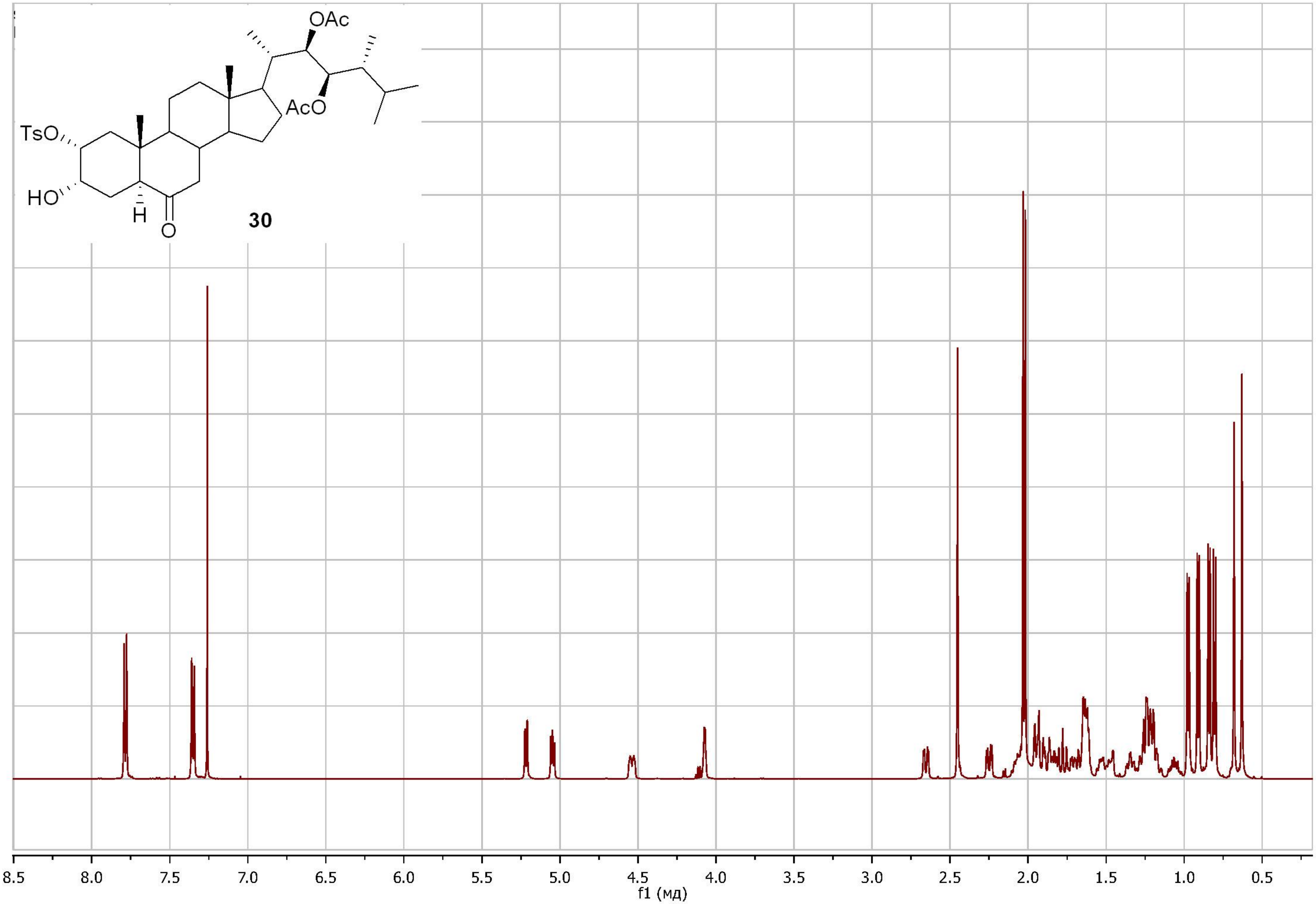
OAc
AcO
TsO
HO
H
O
30
8.5
8.0
7.5
7.0
6.5
6.0
5.5
5.0
4.5
4.0
3.5
3.0
2.5
2.0
1.5
1.0
0.5
f1 (мд)

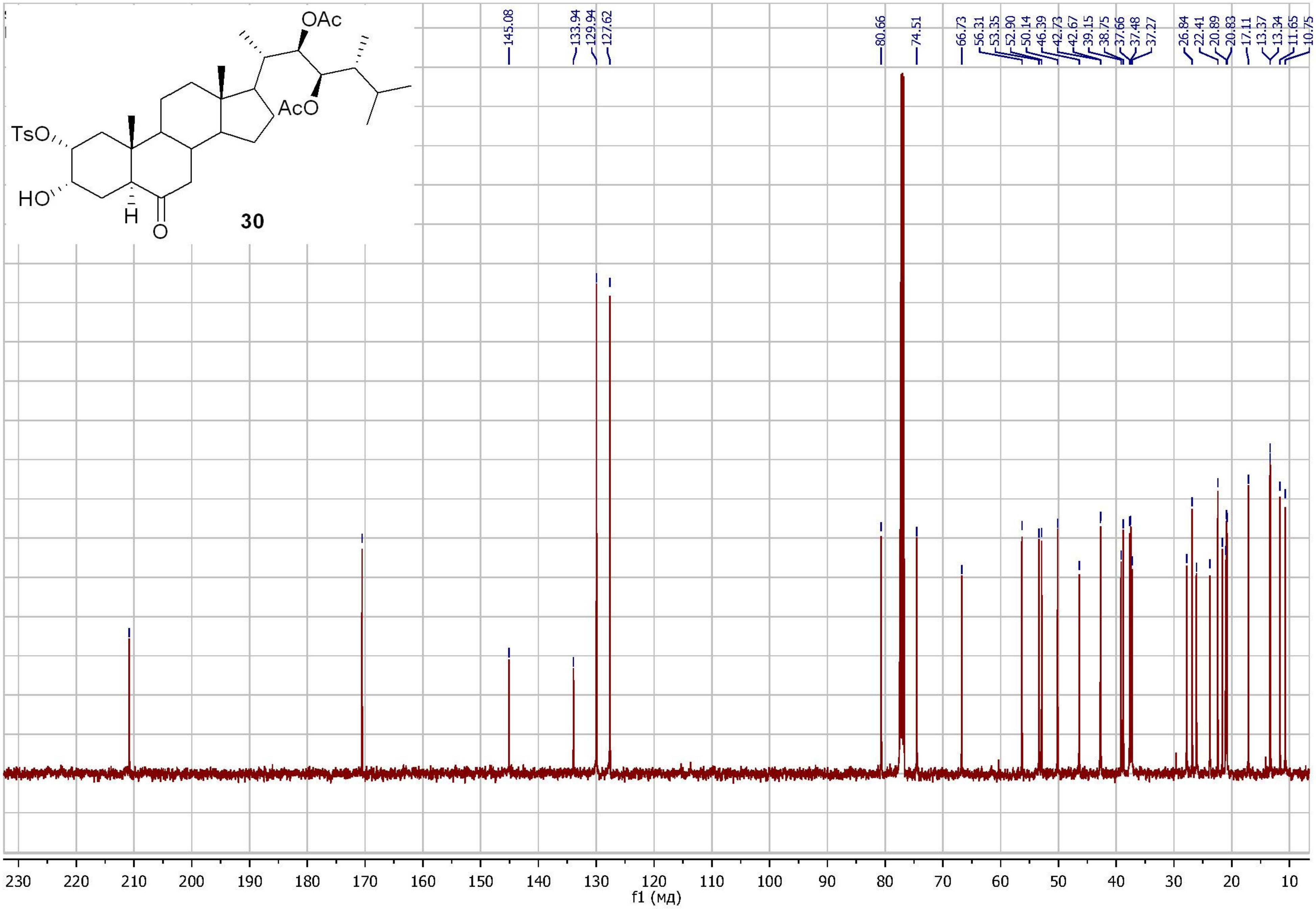
OAc
TsO
HO
H
O
AcO
30
145.08
133.94
129.94
127.62
80.66
74.51
66.73
56.31
53.35
52.90
50.14
46.39
42.73
42.67
39.15
38.75
37.66
37.48
37.27
26.84
22.41
20.89
20.83
17.11
13.37
13.34
11.65
10.75
230 220 210 200 190 180 170 160 150 140 130 120 110 100 90 80 70 60 50 40 30 20 10
f1 (мд)

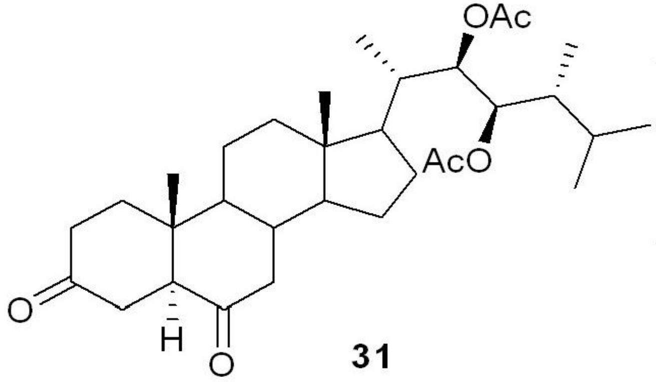
OAc
AcO
O
H
O

31

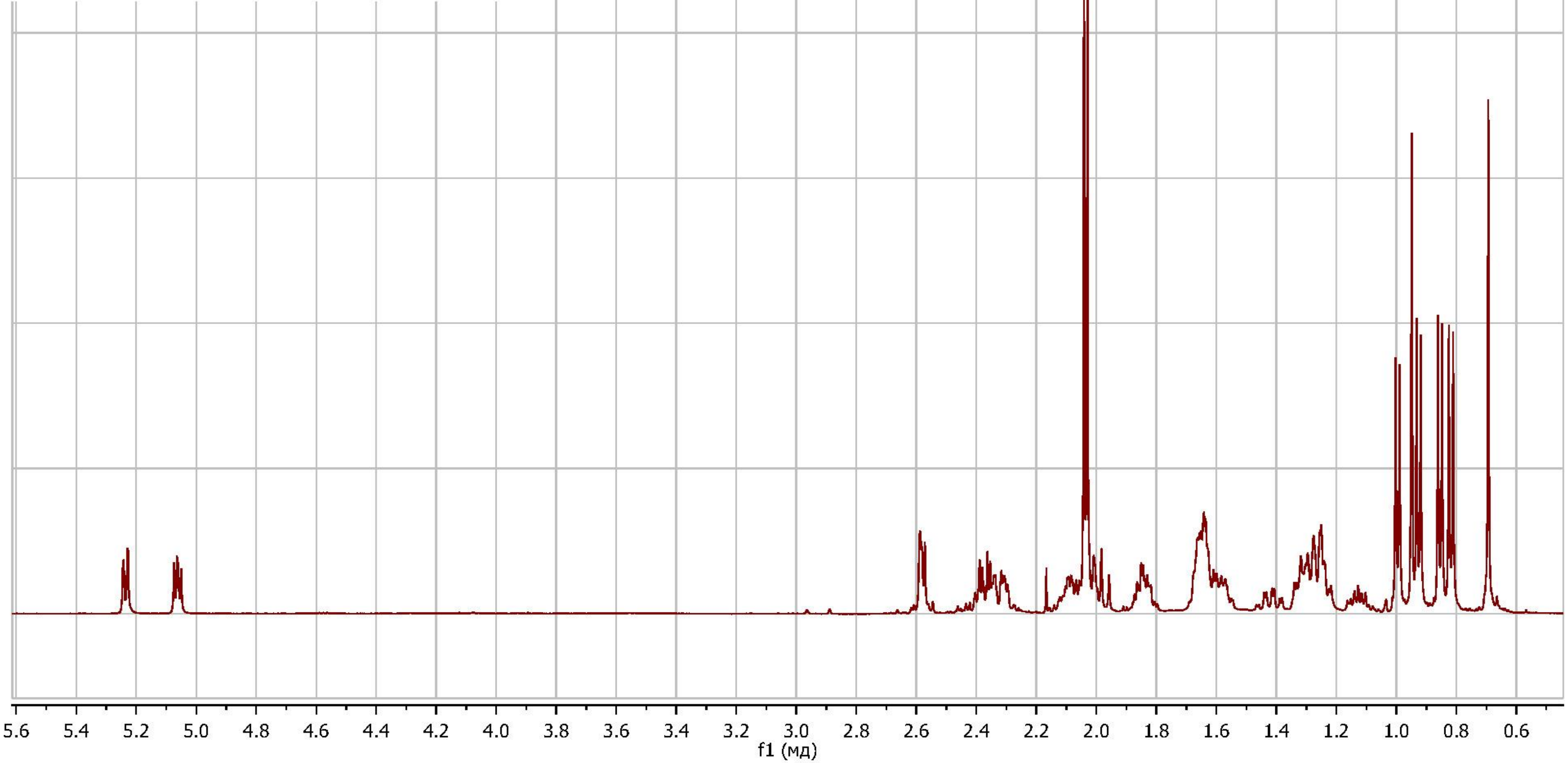
5.6 5.4 5.2 5.0 4.8 4.6 4.4 4.2 4.0 3.8 3.6 3.4 3.2 3.0 2.8 2.6 2.4 2.2 2.0 1.8 1.6 1.4 1.2 1.0 0.8 0.6
f1 (мд)

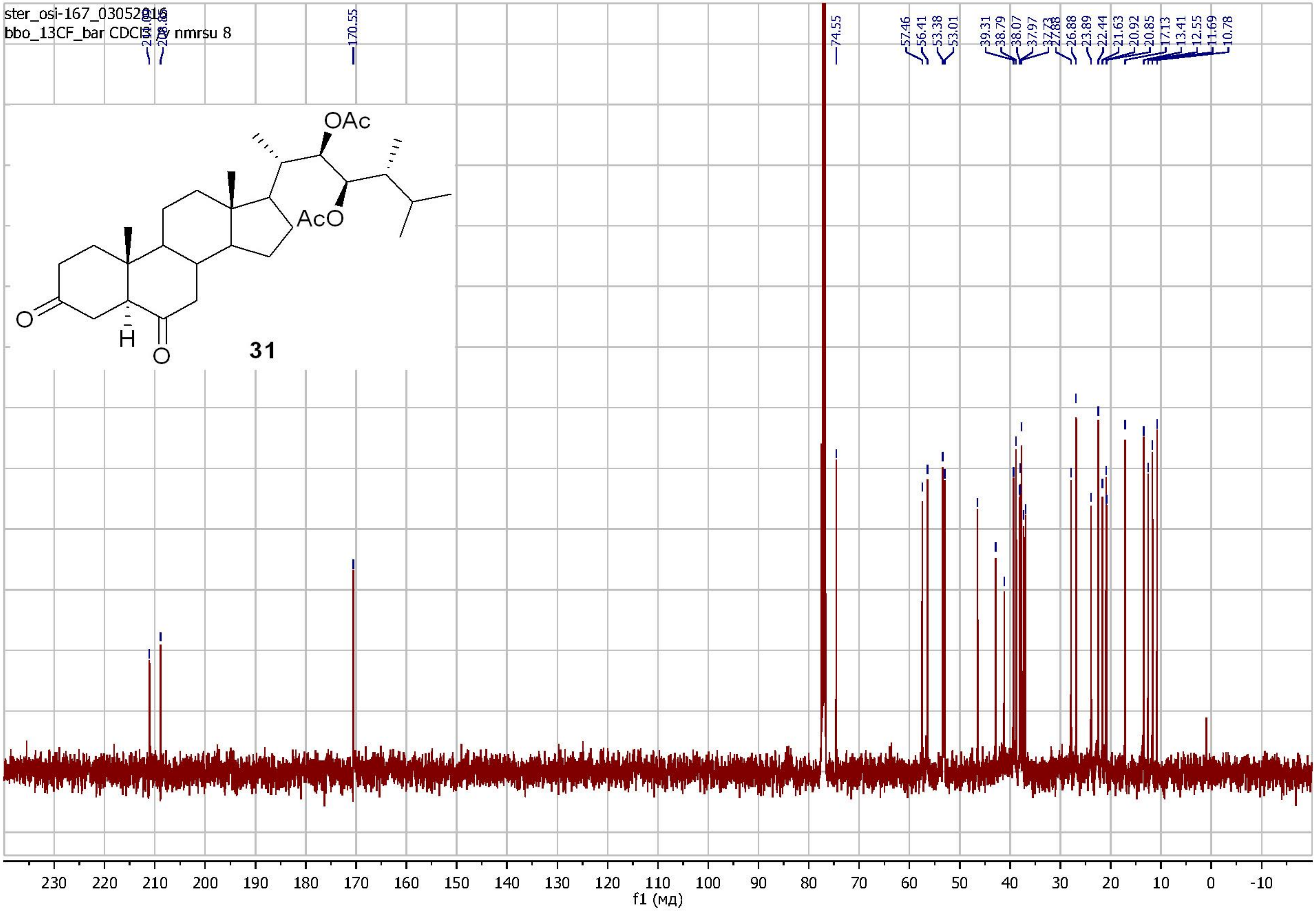
170.55
74.55
57.46
56.41
53.38
53.01
39.31
38.79
38.07
37.97
37.73
27.88
26.88
23.89
22.44
21.63
20.92
20.85
17.13
13.41
12.55
11.69
10.78
OAc
AcO
H
O
31
230
220
210
200
190
180
170
160
150
140
130
120
110
100
90
80
70
60
50
40
30
20
10
0
-10
f1 (мд)

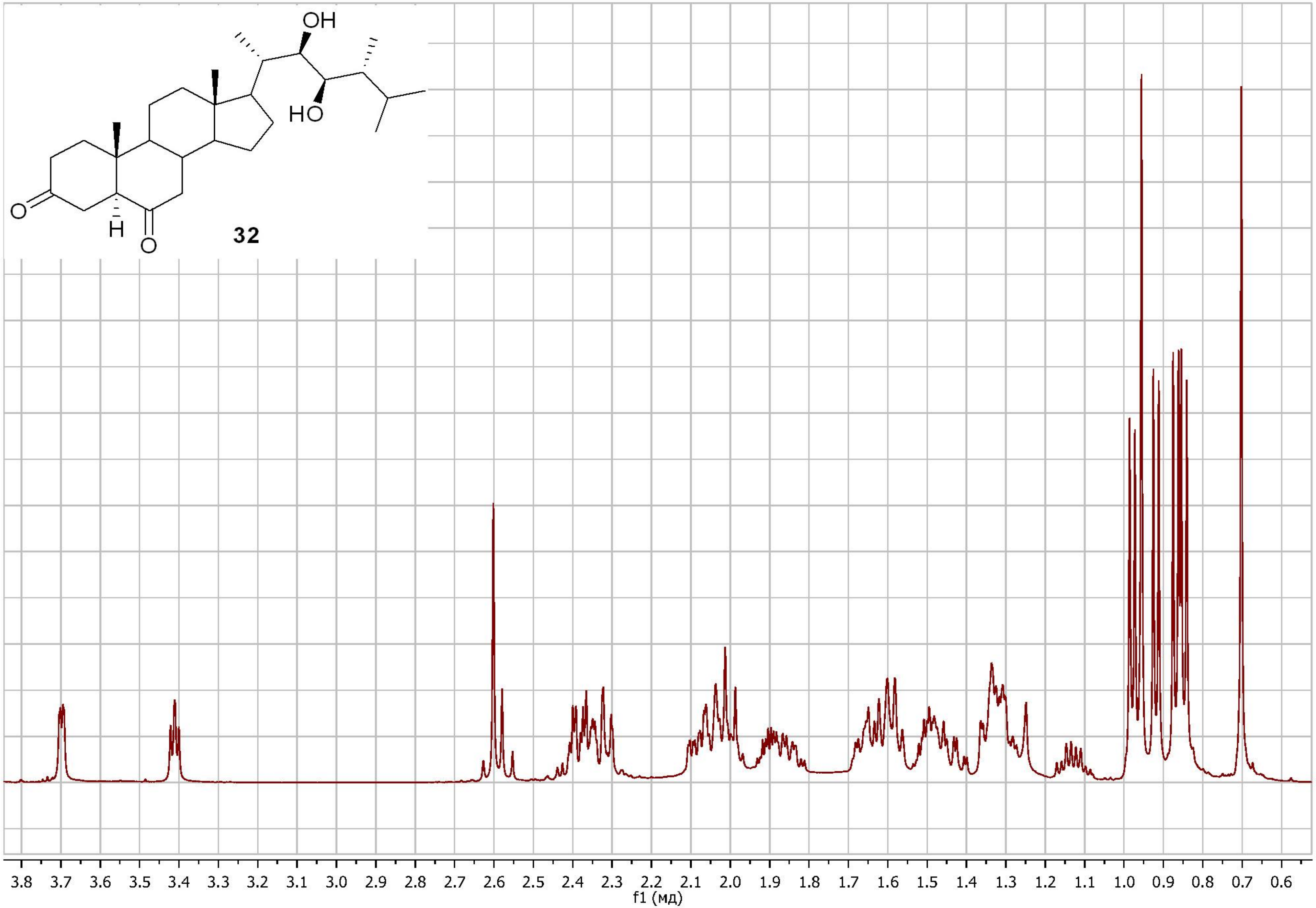
OH
HO
O
H
O
32
3.8 3.7 3.6 3.5 3.4 3.3 3.2 3.1 3.0 2.9 2.8 2.7 2.6 2.5 2.4 2.3 2.2 2.1 2.0 1.9 1.8 1.7 1.6 1.5 1.4 1.3 1.2 1.1 1.0 0.9 0.8 0.7 0.6
f1 (мд)

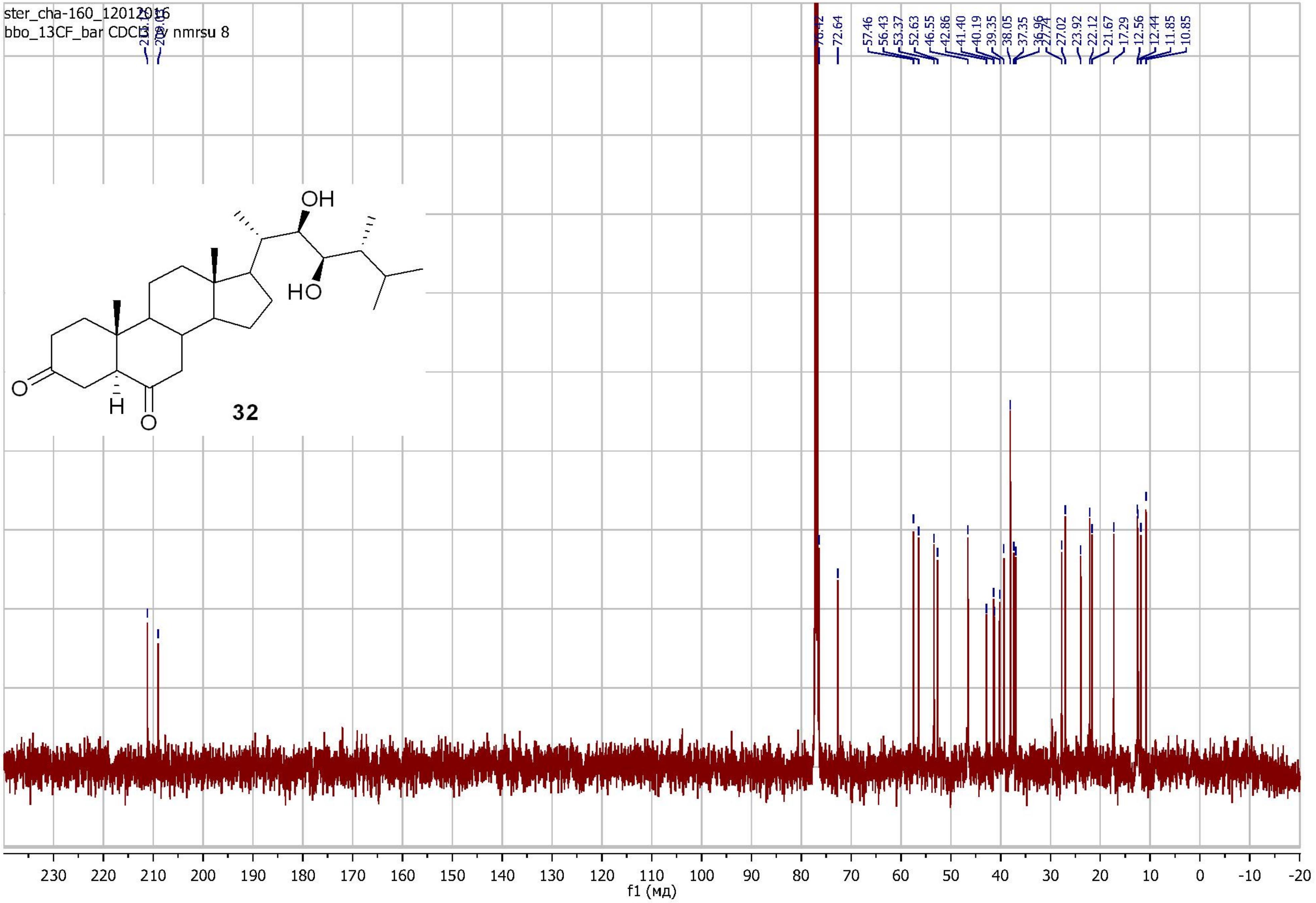

ster_cha-160_12012016
bbo_13CF_bar CDCl3 by nmrsu 8
76.42
72.64
57.46
56.43
53.37
52.63
46.55
42.86
41.40
40.19
39.35
38.05
37.35
36.96
27.74
27.02
23.92
22.12
21.67
17.29
12.56
12.44
11.85
10.85
OH
HO
O
H
O
32
230 220 210 200 190 180 170 160 150 140 130 120 110 100 90 80 70 60 50 40 30 20 10 0 -10 -20
f1 (мд)

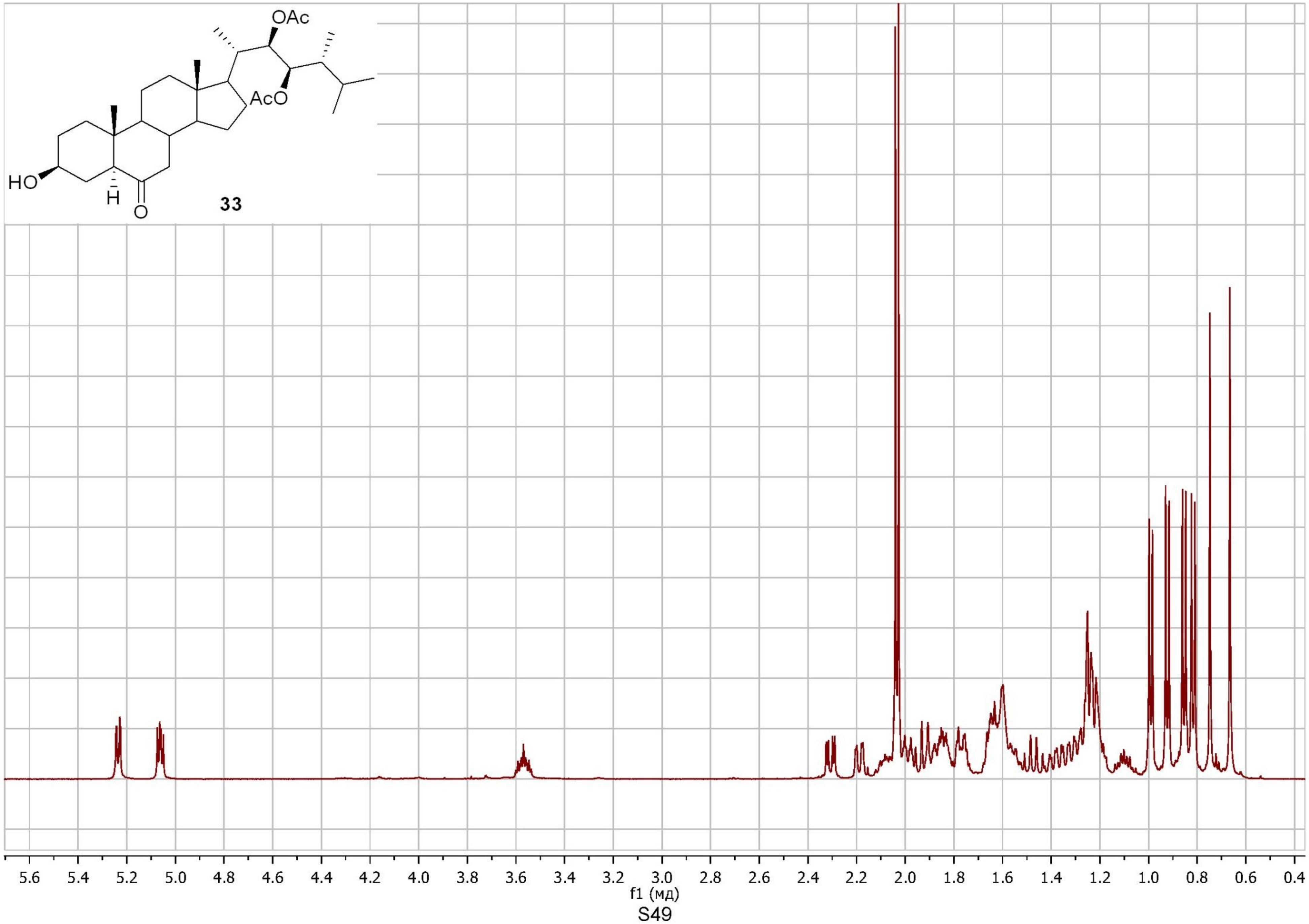

OAc
AcO
HO
H
O
33
5.6 5.4 5.2 5.0 4.8 4.6 4.4 4.2 4.0 3.8 3.6 3.4 3.2 3.0 2.8 2.6 2.4 2.2 2.0 1.8 1.6 1.4 1.2 1.0 0.8 0.6 0.4
f1 (мд)

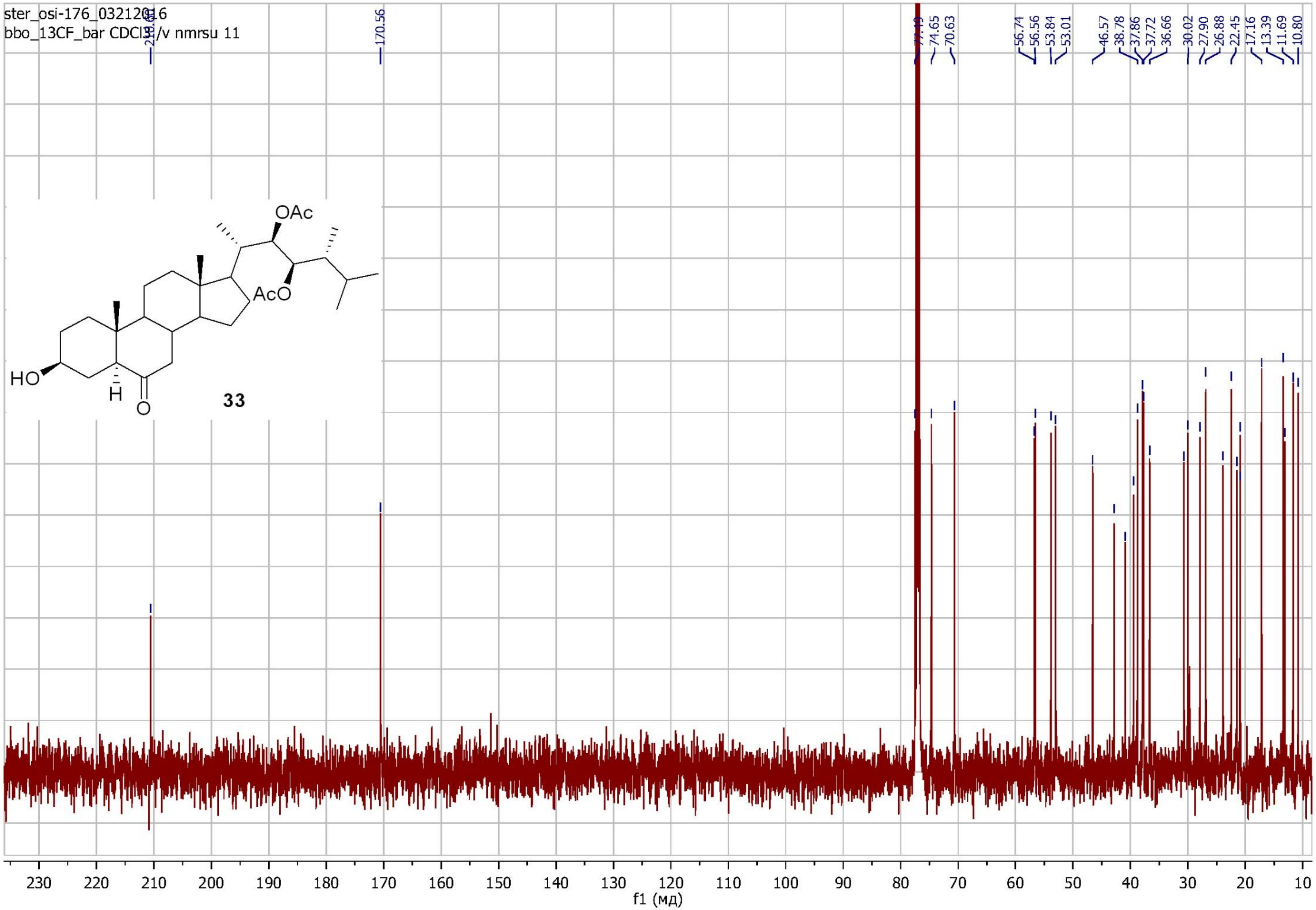
ster_osi-176_03212016
bbo_13CF_bar CDCl3 /v nmrsu 11
170.56
77.19
74.65
70.63
56.74
56.56
53.84
53.01
46.57
38.78
37.86
37.72
36.66
30.02
27.90
26.88
22.45
17.16
13.39
11.69
10.80
OAc
AcO
HO
H
O
33
f1 (мд)

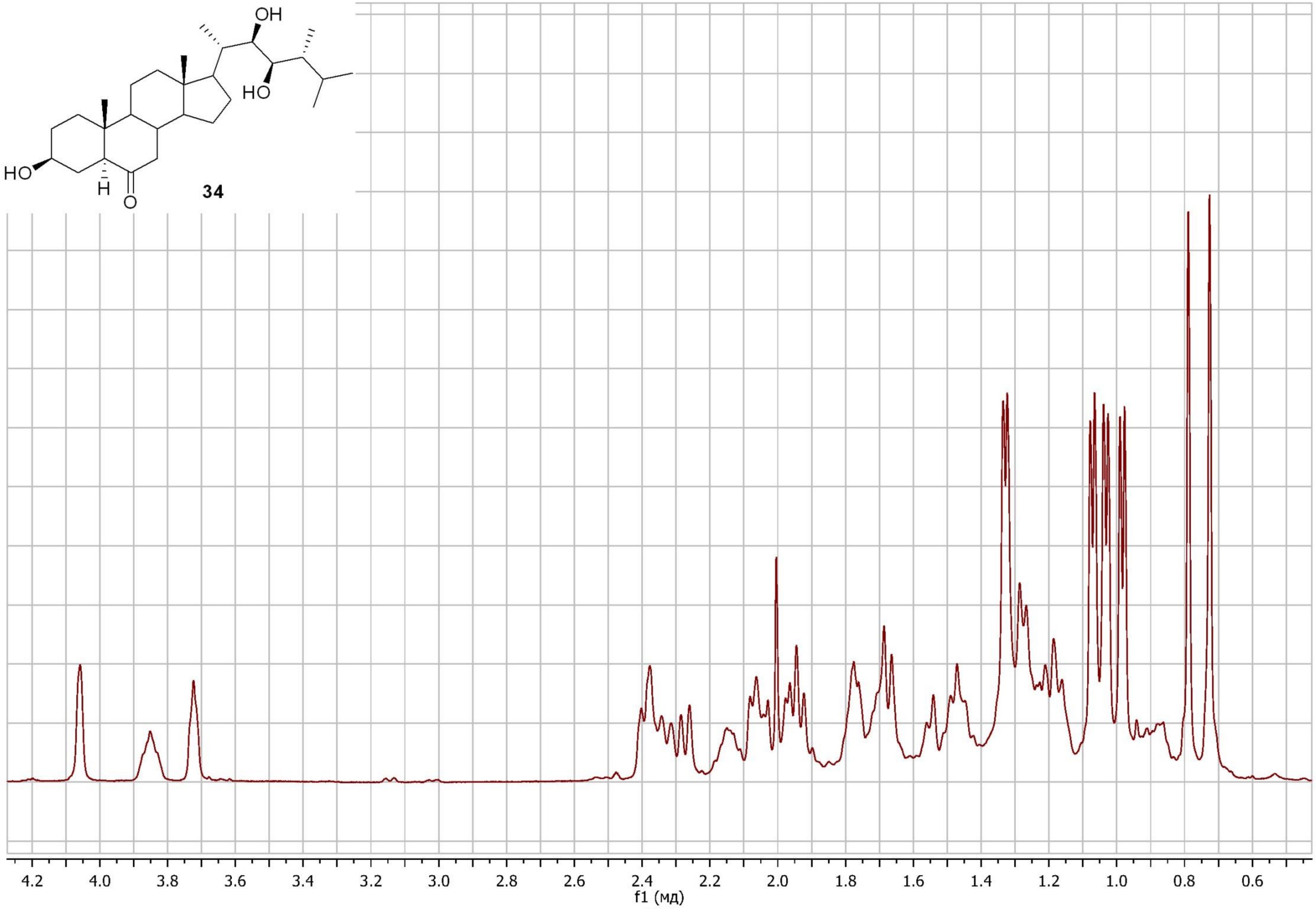
OH
HO
HO
H
O
34
4.2
4.0
3.8
3.6
3.4
3.2
3.0
2.8
2.6
2.4
2.2
2.0
1.8
1.6
1.4
1.2
1.0
0.8
0.6
f1 (мд)

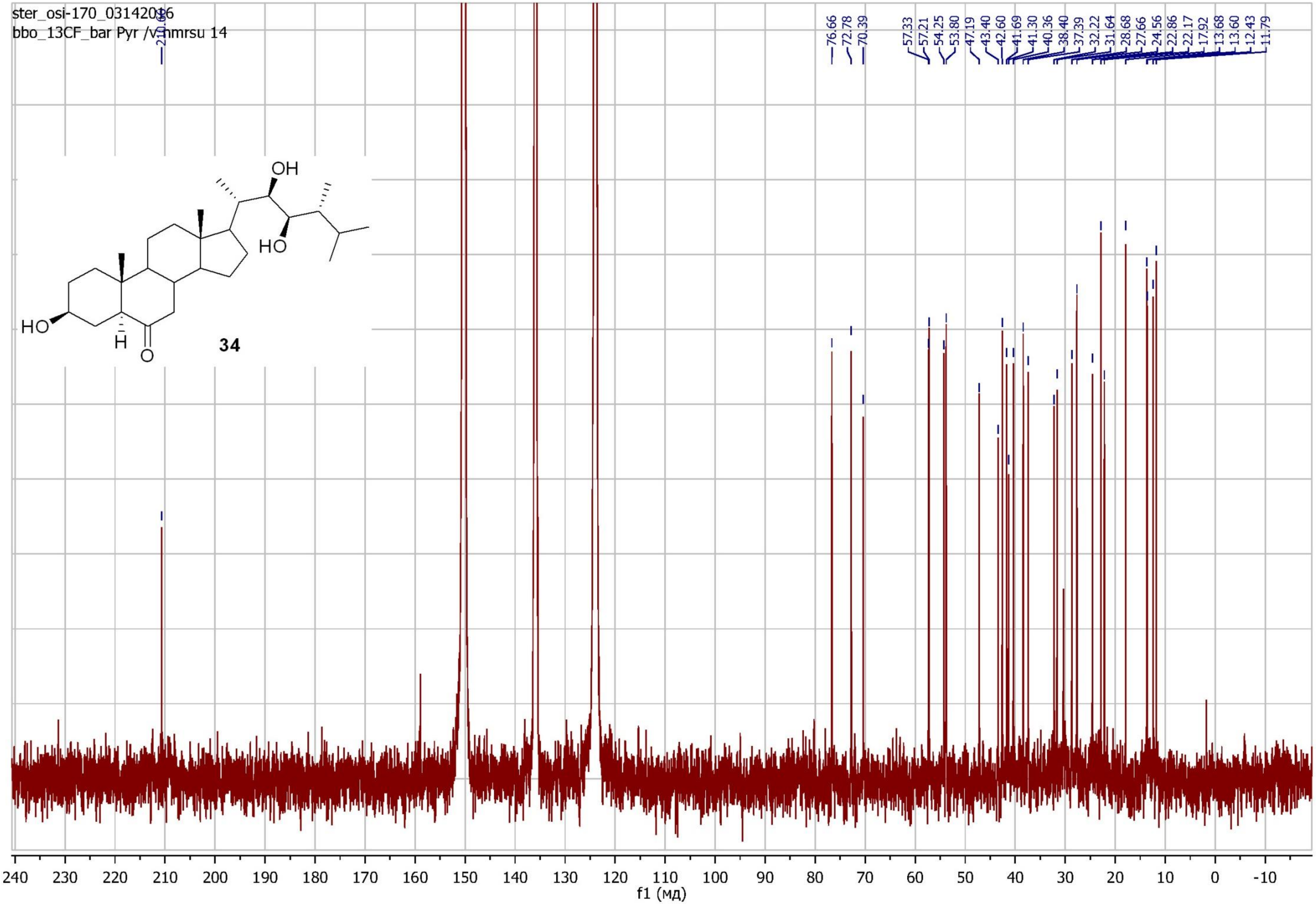

ster_osi-170_03142016
bbo_13CF_bar Pyr /v.nmrsu 14
76.66
72.78
70.39
57.33
57.21
54.25
53.80
47.19
43.40
42.60
41.69
41.30
40.36
38.40
37.39
32.22
31.64
28.68
27.66
24.56
22.86
22.17
17.92
13.68
13.60
12.43
11.79
OH
HO
HO
H
O
34
240 230 220 210 200 190 180 170 160 150 140 130 120 110 100 90 80 70 60 50 40 30 20 10 0 -10
f1 (мд)

ster_osi-121_09292015
qnp_1H_bar CDCl3 /v nmrsu 8

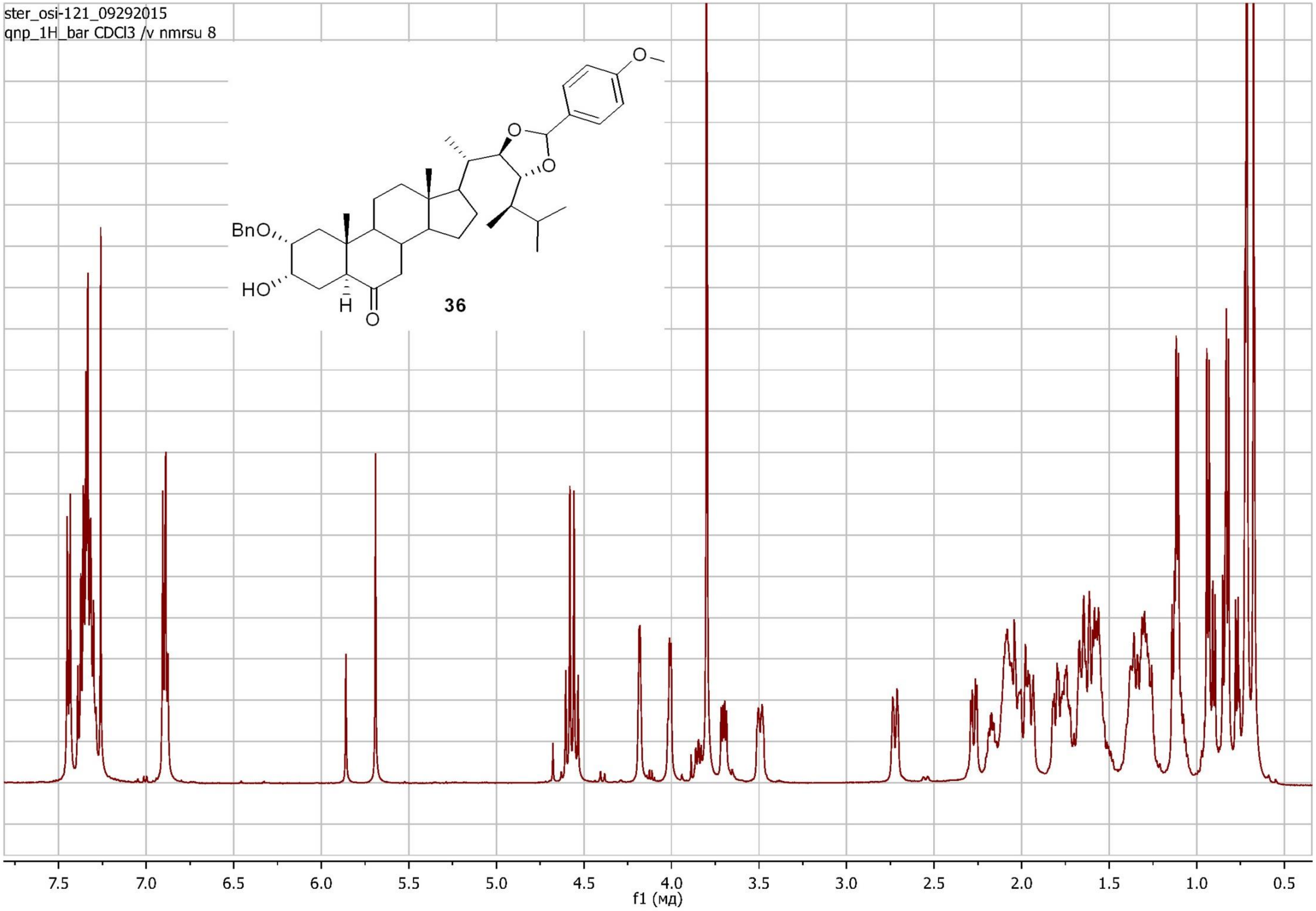

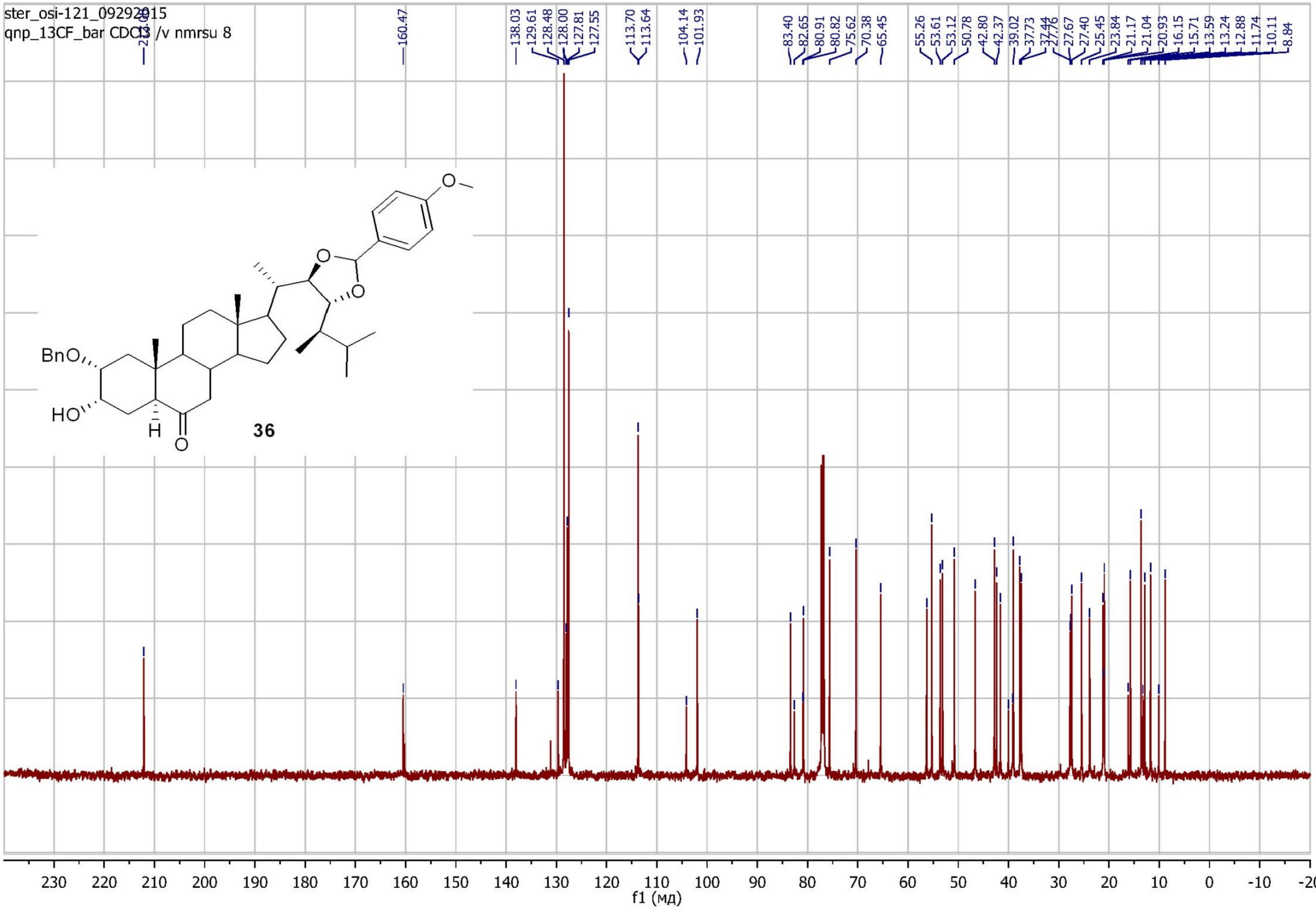

ster_osi-121_09292015
qnp_13CF_bar CDCl3 /v nmrsu 8
160.47
138.03
129.61
128.48
128.00
127.81
127.55
113.70
113.64
104.14
101.93
83.40
82.65
80.91
80.82
75.62
70.38
65.45
55.26
53.61
53.12
50.78
42.80
42.37
39.02
37.73
27.67
27.40
25.45
23.84
21.17
21.04
20.93
16.15
15.71
13.59
13.24
12.88
11.74
10.11
8.84
BnO
HO
H
O
36
230 220 210 200 190 180 170 160 150 140 130 120 110 100 90 80 70 60 50 40 30 20 10 0 -10 -20
f1 (мд)

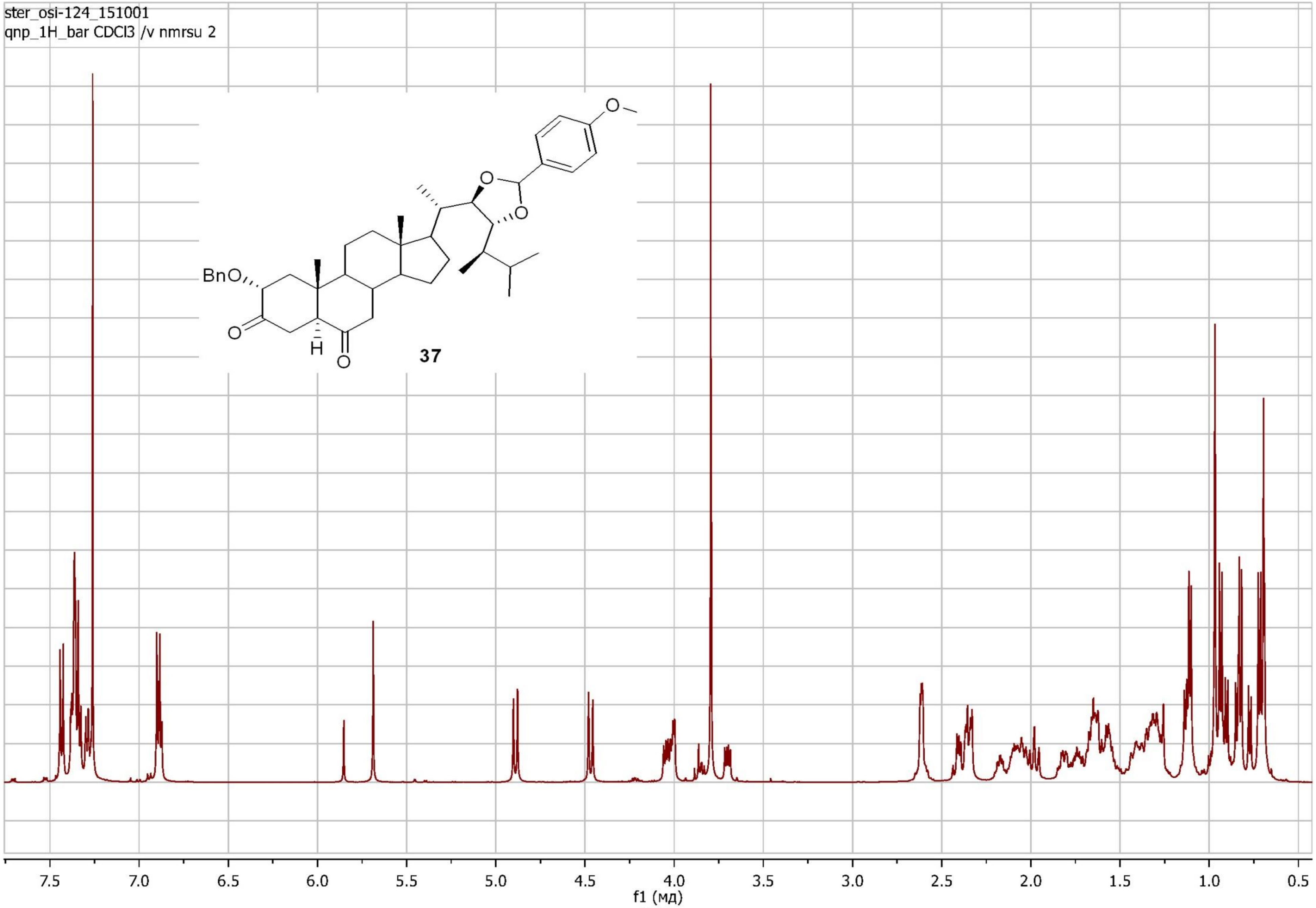
ster_osi-124_151001
qnp_1H_bar CDCl3 /v nmrsu 2
BnO
O
H
O
O
O
O
37
7.5
7.0
6.5
6.0
5.5
5.0
4.5
4.0
3.5
3.0
2.5
2.0
1.5
1.0
0.5
f1 (мд)

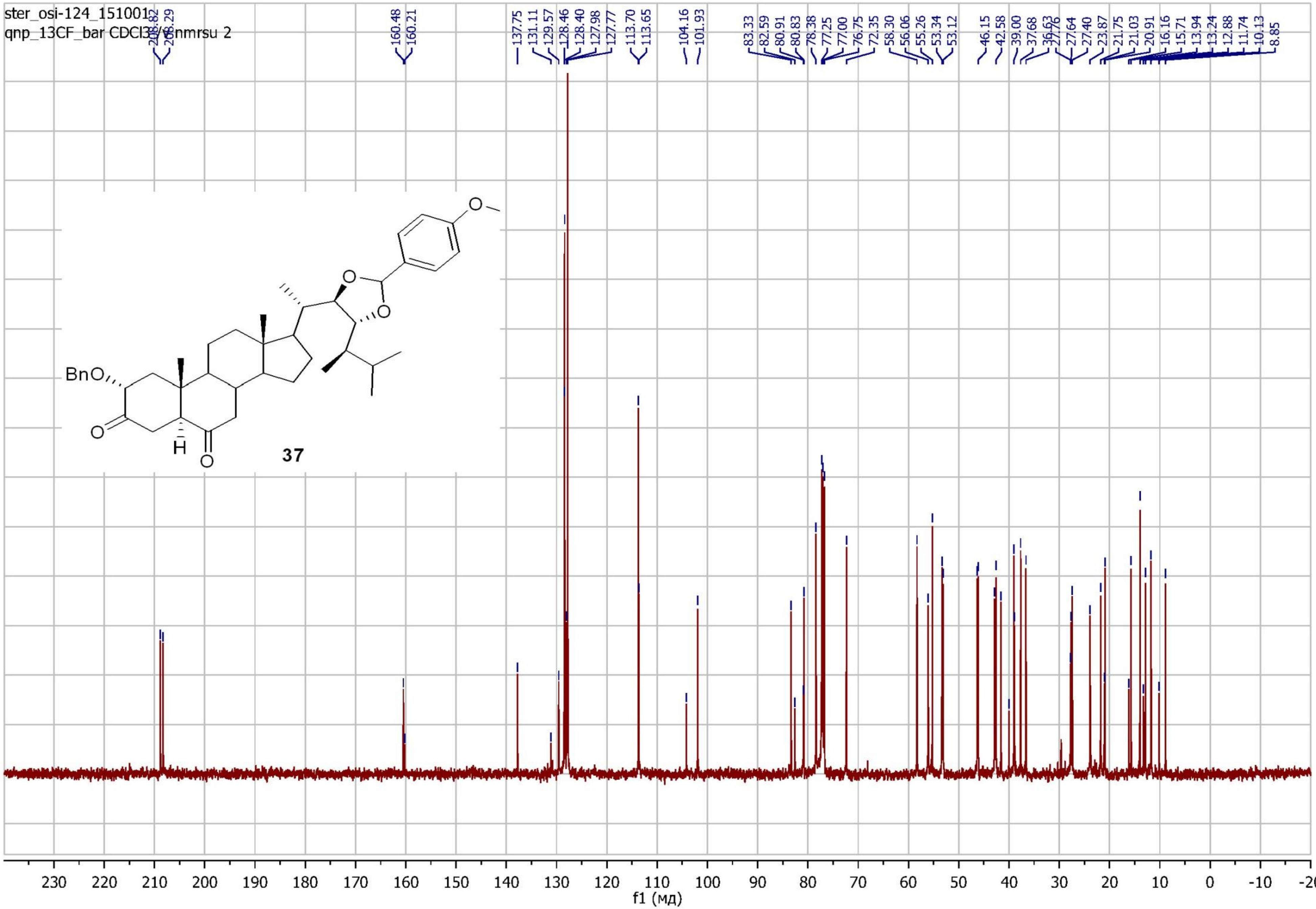
ster_osi-124_151001
qnp_13CF_bar CDCl3
160.48
160.21
137.75
131.11
129.57
128.46
128.40
127.98
127.77
113.70
113.65
104.16
101.93
83.33
82.59
80.91
80.83
78.38
77.25
77.00
76.75
72.35
58.30
56.06
55.26
53.34
53.12
46.15
42.58
39.00
37.68
36.63
27.76
27.64
27.40
23.87
21.75
21.03
20.91
16.16
15.71
13.94
13.24
12.88
11.74
10.13
8.85
BnO
O
H
37
f1 (мд)

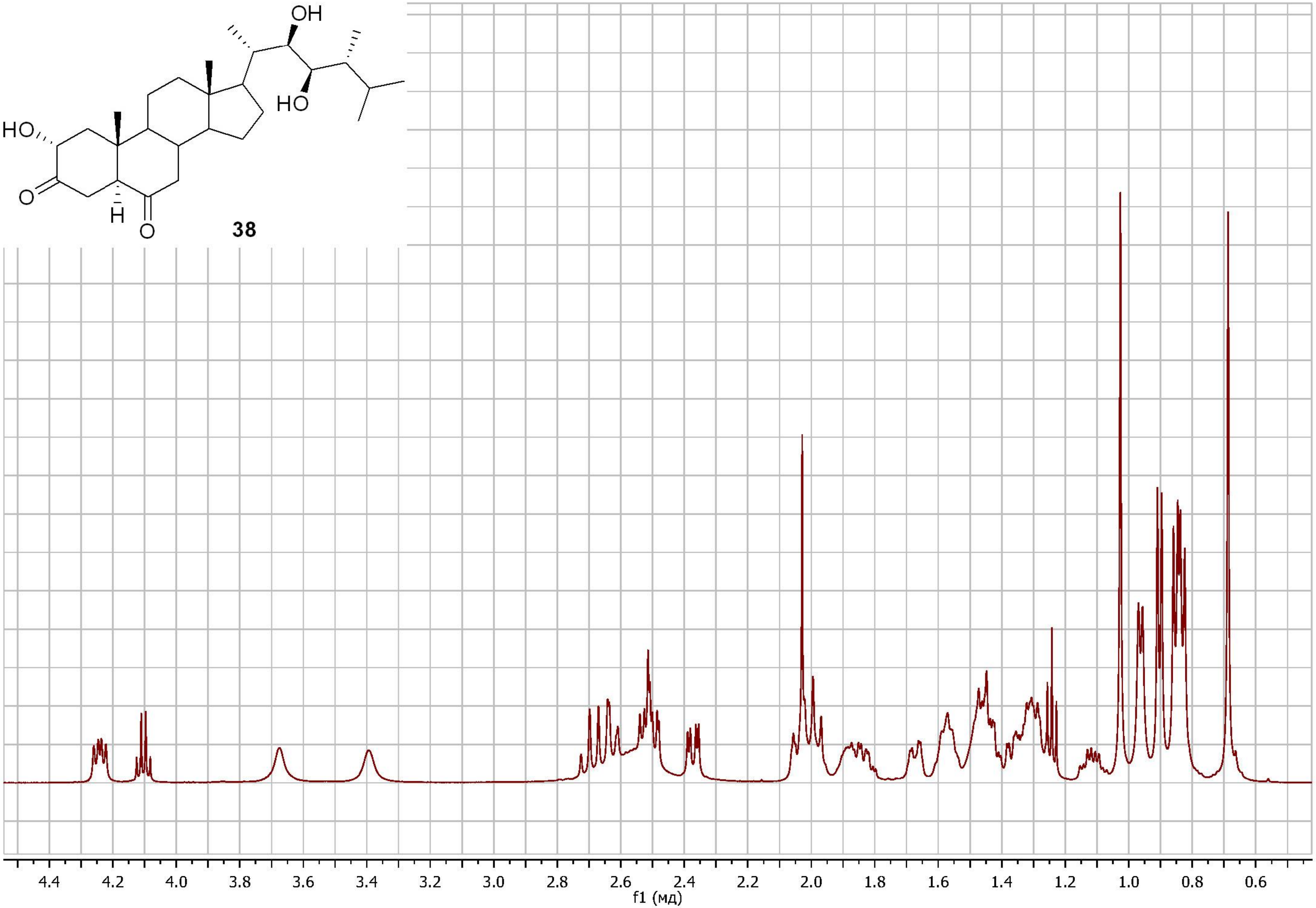
OH
HO
HO
O
H
O
38
4.4
4.2
4.0
3.8
3.6
3.4
3.2
3.0
2.8
2.6
2.4
2.2
2.0
1.8
1.6
1.4
1.2
1.0
0.8
0.6
f1 (мд)

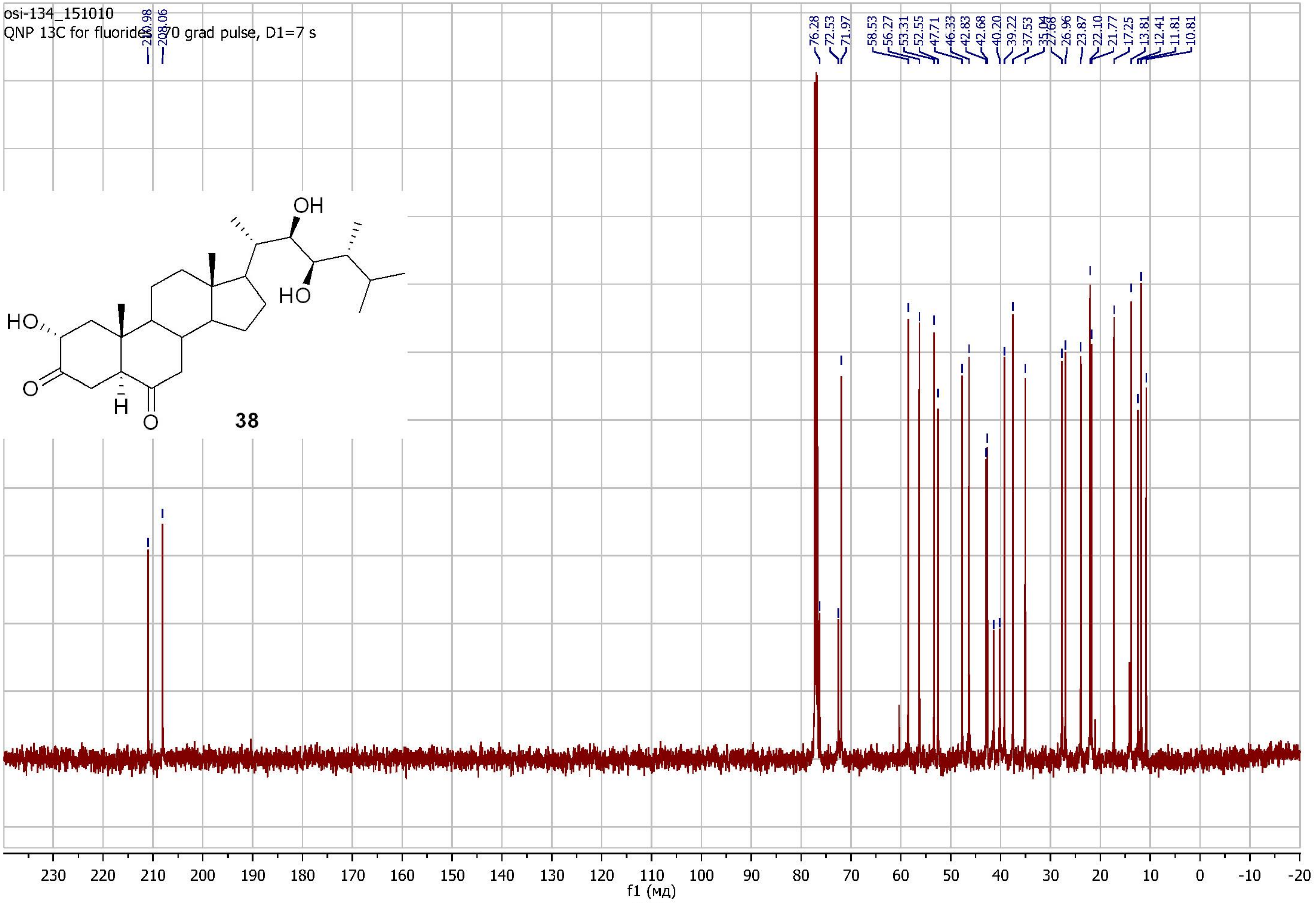
osi-134_151010
QNP 13C for fluorides 70 grad pulse, D1=7 s
210.98
208.06
76.28
72.53
71.97
58.53
56.27
53.31
52.55
47.71
46.33
42.83
42.68
40.20
39.22
37.53
35.04
27.68
26.96
23.87
22.10
21.77
17.25
13.81
12.41
11.81
10.81
OH
HO
HO
O
H
O
38
230 220 210 200 190 180 170 160 150 140 130 120 110 100 90 80 70 60 50 40 30 20 10 0 -10 -20
f1 (мд)
